# Efficient synthesis of β’-amino-α,β-unsaturated ketones

**DOI:** 10.3762/bjoc.9.52

**Published:** 2013-03-06

**Authors:** Isabelle Abrunhosa-Thomas, Aurélie Plas, Nishanth Kandepedu, Pierre Chalard, Yves Troin

**Affiliations:** 1 Clermont Université, ENSCCF, Institut de Chimie de Clermont-Ferrand (ICCF), BP 10448, F-63000 Clermont-Ferrand, France; 2 CNRS, UMR 6296, Institut de Chimie de Clermont-Ferrand (ICCF), BP 80026, F-63171 Aubière, France; 3 Clermont Université, Institut de Chimie de Clermont-Ferrand, BP 10448, F-63000 Clermont-Ferrand, France

**Keywords:** β’-amino-α,β-unsaturated ketones, Horner–Wadsworth–Emmons reaction, stereoselective synthesis

## Abstract

A general and simple procedure to access chiral β'-amino-α,β-enones, in seven steps, from an α,β unsaturated ester has been described. The use of a Horner–Wadsworth–Emmons reaction as a key step for generating the β'-amino-α,β-enones, permits access to a range of substrates under mild conditions and in moderate to high yield.

## Introduction

**Scheme 1:**
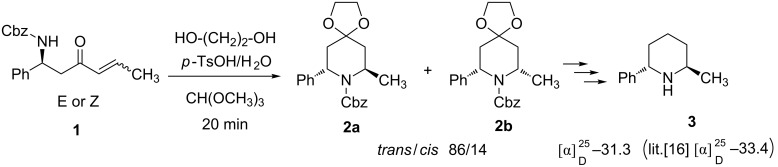
Asymmetric synthesis of 2-methyl-6-phenyl piperidine.

Compounds incorporating β-amino ketone functionality are prevalent in many natural products of biological importance [1]. This versatile synthon has been extensively used in the construction of β-amino acids [2], β-amino alcohols [3], and homoallylic amines [4–5], and can serve as building blocks for the preparation of nitrogen-containing molecules often found in medicinal chemistry [6–10]. Thus, the development of efficient and stereoselective reactions for a useful approach to chiral β-amino ketones is still of importance. One of the most powerful approaches is the Mannich reaction, which can be conducted under different protocols in which the stereoselectivity of the reaction can be introduced through the use of a chiral catalyst [9–10] (Lewis acid, Brønsted acids, L-proline, *Cinchona* alkaloids derivatives, thioureas, etc.), or by the addition of chiral amines to α,β-unsaturated esters [11–12] or the reaction of chiral imines with enolates derived from Weinreb amides [13–14]. In previous work on the asymmetric synthesis of 2,6-disubstituted piperidines by C–N bond formation, we demonstrated that intramolecular aza-Michael ”type” cyclisation [15] using a β'-carbamate-α,β-unsaturated ketone predominantly induces the formation of a piperidine ring with the 2,6-*trans* configuration (Scheme 1).

The relative stereochemistry of piperidine **2a** was confirmed by further transformation to the known compound **3** [16–17] with 94% ee. In order to establish this new approach as a general method for the preparation of chiral 2,6-disubstituted piperidines, we wish to report here a facile synthetic route to various β’-carbamate-α,β-unsaturated ketones in good overall yields and good enantioselectivities.

## Results and Discussion

**Scheme 2:**
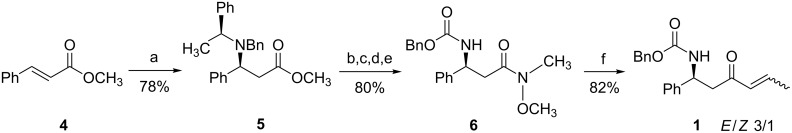
(a) Davies amine, BuLi, THF, −78 °C; dr ≥ 94% ; (b) H_2_, Pd(OH)_2_, MeOH; (c) Na_2_CO_3_, PhCH_2_CO_2_Cl, CH_2_Cl_2_/H_2_O; (d) NaOH 1 N, MeOH; (e) CDI, *N,O*-dimethylhydroxylamine·HCl, (f) Mg, 1-bromo-2-propene, THF.

In a preliminary approach, preparation of β-amino ketones was envisaged through a nucleophilic addition reaction of Grignard reagents to *N*-carbamoyl β-amino Weinreb amides (Scheme 2) [18].

**Scheme 3:**
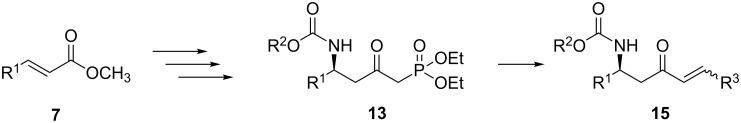
Modified synthetic route to**15**.

Conjugate addition of (*R*)-*N*-benzyl-*N*-methylbenzylamide to methyl cinnamate under basic conditions led to β–aminoester **5** with high diastereoselectivity (dr >94%) [11–12]. Subsequent transformation of the ester moiety to a Weinreb amide [18] followed by changing the nitrogen protecting group to a carbamate furnished the key intermediate **6**, which could be further alkylated with Grignard reagents to give β’-amino protected α,β-enone **1** in good overall yield and high enantiomeric excess. As Grignard reagents did not allow the use of a wide range of functional groups and sometimes gave bad overall yields, we devised a general and simple method to easily access a variety of β’-amino-α,β-unsaturated ketones by a more convenient route using the Horner–Wadsworth–Emmons reaction [19–20] as the key step, as described in Scheme 3.

**Scheme 4:**
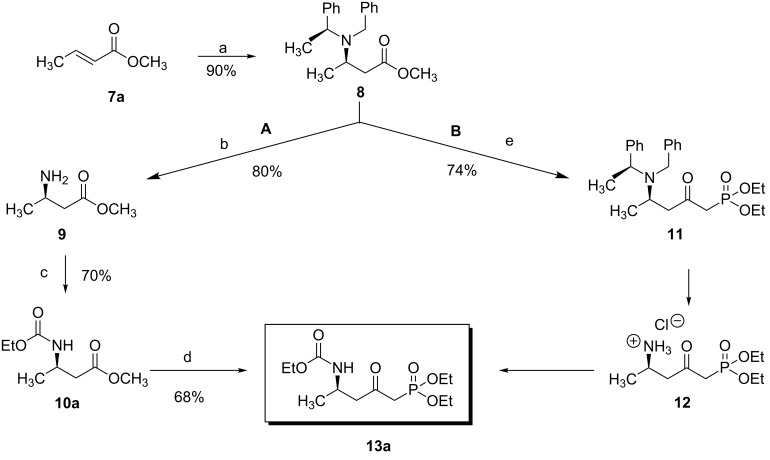
Possible pathways to obtain phosphonate **13** (a) Davies amine, BuLi, THF, −78 °C; dr ≥ 95%; (b) H_2_, Pd(OH)_2_/C, MeOH, 60 psi; (c) Na_2_CO_3_, R^2^CO_2_Cl, CH_2_Cl_2_/H_2_O; (d) and (e) BuLi, (EtO)_2_P(O)Me, THF, −78 °C.

In order to gain access to phosphonates **13**, in a general and convergent process, two ways were investigated (Scheme 4). In route **A**, we planned to obtain the desired compound by using a similar strategy to that which we have described previously (see above): chiral induction was obtained through the addition of Davies amine, furnishing **8**. Hydrogenation of compound **8** followed by *N*-protection as a carbamate would furnish the β-amino ester precursor of the phosphonate **13**. In route **B**, the most convergent, the preparation of the phosphonate was envisaged in the first step; then, hydrogenation would furnish amino phosphonate. In method **A** the last step to synthesize **13** would be a nucleophilic addition of diethyl methylphosphonate under basic conditions, whereas for method **B**, the final step would be the *N*-protection of the amino phosphonate as a carbamate.

**Scheme 5:**
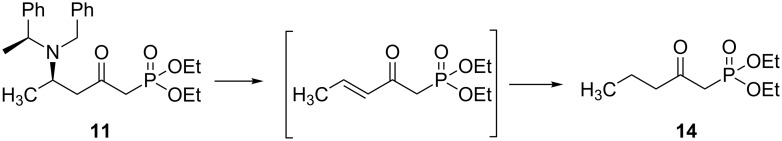
Synthesis of compound **14**.

The two routes were then tested, starting from methyl crotonate (**7a**, R^1^ = Me) as a model substrate (Scheme 4). While compounds **9** and **10a** were obtained in reasonable yields (80 and 70%, respectively) in route **A**, the hydrogenation of **11** (obtained in a 74% yield from **8**) to **12** did not proceed to provide the expected compound under various conditions (methanol in acid conditions, using either Pd(OH)_2_/C under H_2_ pressure (60 psi) or Pd/C under reflux in the presence of ammonium formate). Instead, the formation of **14** [21] was observed, resulting from β-elimination and reduction of the transient double bond (Scheme 5).

**Scheme 6:**
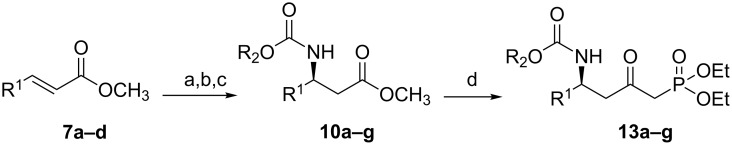
General synthesis of compound **13** (a) Davies amine, BuLi, THF, −78 °C; (b) H_2_, Pd(OH)_2_/C, MeOH; (c) Na_2_CO_3_, R^2^CO_2_Cl, CH_2_Cl_2_/H_2_O; (d) BuLi, (EtO)_2_P(O)Me, THF, −78 °C.

Thus, we focused on route **A**, and after optimization of the reaction conditions, we found that the transformation of **7a** to **10a** could be done without purification. Hence, the addition of enantiopure lithium *N*-benzyl-*N*-α-methylbenzylamide to α,β-unsaturated ester **7** followed by hydrogenation to the corresponding primary amine and further protection as a carbamate gave the β-amino methylester **10**. At this stage, the ester function was transformed into the ketophosphonate **13** by treatment with 2.5 equivalents of the lithium anion of diethyl methylphosphonate [22–28] in THF at −78 °C, in moderate to good yields (Scheme 6, Table 1).

**Table 1:** Formation of the ketophosphonates **13**.

entry	ester **7**	amino ester **10**	yield (%)	phosphonate **13**	yield (%)

1	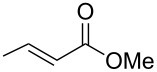 **7a**	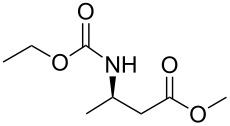 **10a**	57	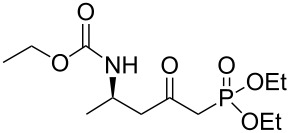 **13a**	68
2	**7a**	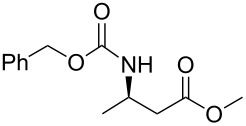 **10b**	62	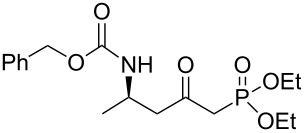 **13b**	57

3	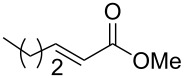 **7b**	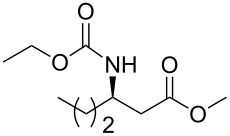 **10c**	77	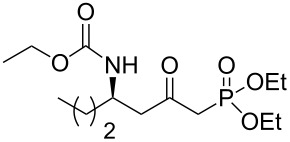 **13c**	65

4	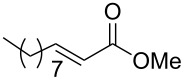 **7c**	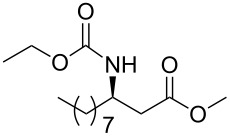 **10d**	73	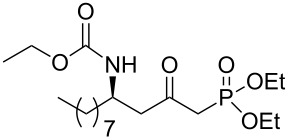 **13d**	66

5	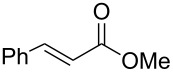 **7d**	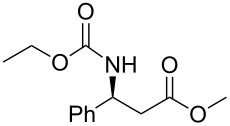 **10e**	72	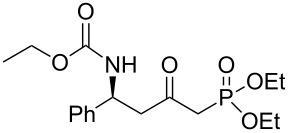 **13e**	58
6	**7d**	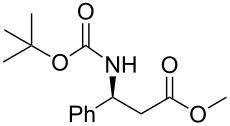 **10f**	76	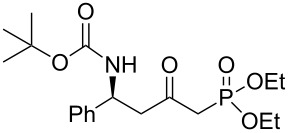 **13f**	62
7	**7d**	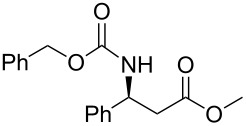 **10g**	66	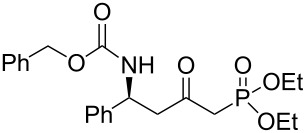 **13g**	66

**Scheme 7:**
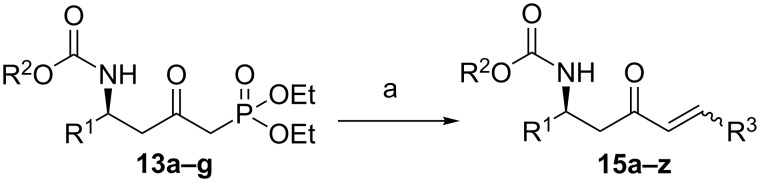
Optimization of conditions for the Horner–Wadsworth–Emmons reaction.

Over the years, many examples of base-promoted Horner–Wadsworth–Emmons (HWE) reactions have been reported in the literature, and various combinations of bases and solvents (K_2_CO_3_/CH_3_CN[23], DBU/THF[25], NaH/THF[29], Et_3_N/LiCl/CH_3_CN[30] or Ba(OH)_2_/(THF/H_2_O)[31]) have been used. We subjected our substrate **13a** to three of those mild sets of conditions (Scheme 7, Table 2), which, after reaction with the benzaldehyde, will furnish the chiral amino ketone **15a**.

**Table 2:** Horner–Wadsworth–Emmons optimal conditions for **15a**.

phosphonate **13a**	aldehyde	conditions (a)	amino ketone **15a**	yield (%)

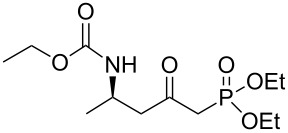	benzaldehyde	1 equiv Et_3_N/LiCl/CH_3_CN, 3 h	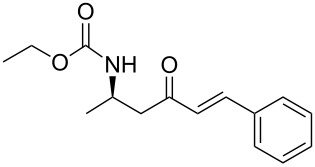	75
		1 equiv DBU/THF, 2 h		80
		1.3 equiv Ba(OH)_2_/(THF/H_2_O), 1 h		95

As illustrated in Table 2, we found that the use of 1.3 equiv of Ba(OH)_2_ THF/H_2_O (40/1) furnished the optimal yield of 95% with our model substrate. Those conditions were then applied to a wide range of functionalized aldehydes with phosphonate **13a–g**, giving amino ketone **15a–z** in good to excellent yields and high *E*/*Z* ratio (≥ 95%). The results are presented in Table 3.

**Table 3:** Formation of the β’-amino-α,β-unsaturated ketones **15** under Ba(OH)_2_ conditions.

entry	phosphonate **13**	aldehyde	amino ketone **15**	yield (%)

1	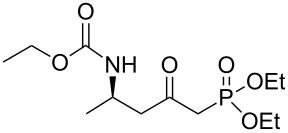 **13a**	benzaldehyde	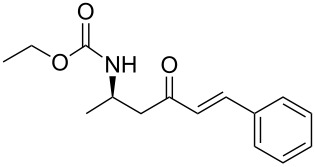 **15a**	95
2	**13a**	*o*-nitrobenzaldehyde	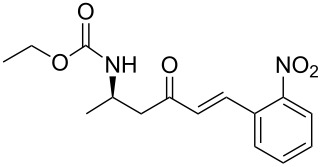 **15b**	89
3	**13a**	*m*-nitrobenzaldehyde	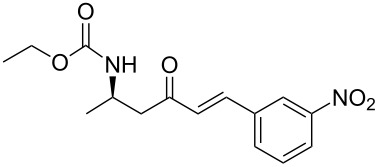 **15c**	86
4	**13a**	*p*-nitrobenzaldehyde	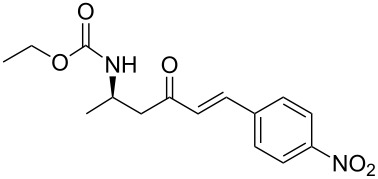 **15d**	91
5	**13a**	*p*-methoxybenzaldehyde	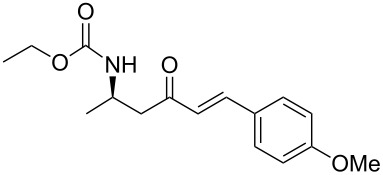 **15e**	79
6	**13a**	*o*-bromobenzaldehyde	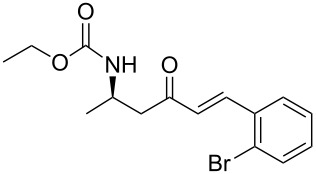 **15f**	91
7	**13a**	*p*-bromobenzaldehyde	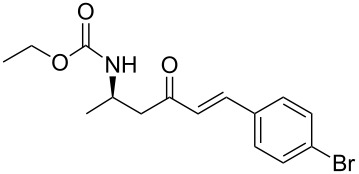 **15g**	89
8	**13a**	*2*-chloro-5-nitrobenzaldehyde	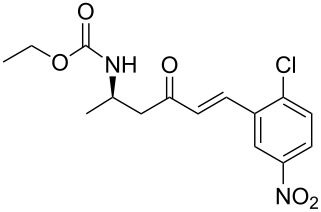 **15h**	95
9	**13a**	pyridine-3-carboxaldehyde	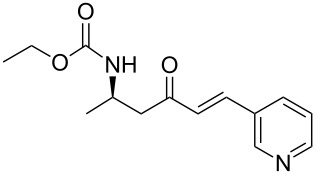 **15i**	87
10	**13a**	(*E*)-ethyl-4-oxo-2-butenoate	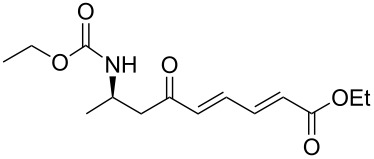 **15j**	85
11	**13a**	ethylglyoxylate	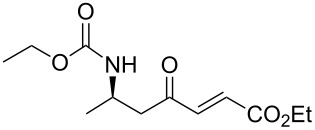 **15k**	53
12	**13a**	butanal	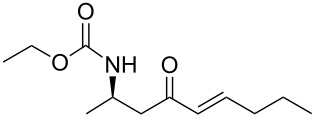 **15l**	95
13	**13a**	nonanal	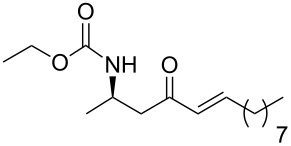 **15m**	88

14	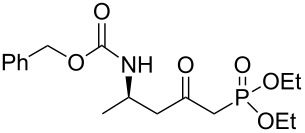 **13b**	benzaldehyde	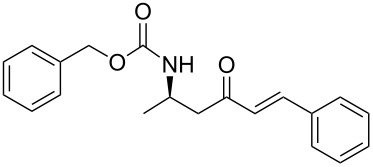 **15n**	96
15	**13b**	decanal	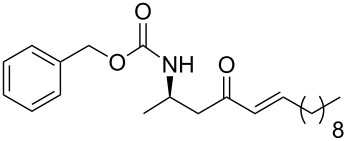 **15o**	78
16	**13b**	dodecanal	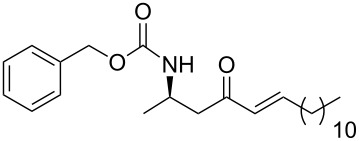 **15p**	91

17	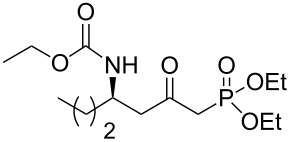 **13c**	benzaldehyde	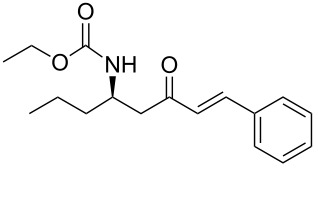 **15q**	91
18	**13c**	*p*-nitrobenzaldehyde	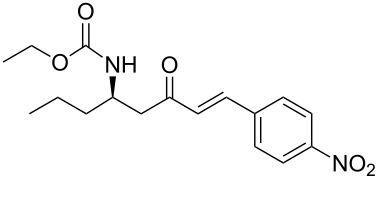 **15r**	84

19	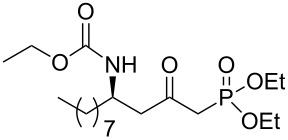 **13d**	benzaldehyde	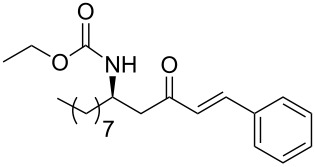 **15s**	94
20	**13d**	ethanal	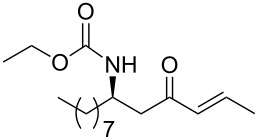 **15t**	78
21	**13d**	butanal	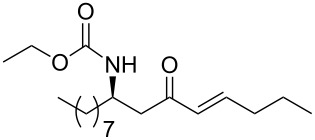 **15u**	95

22	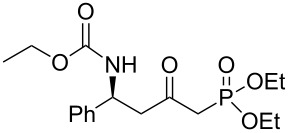 **13e**	benzaldehyde	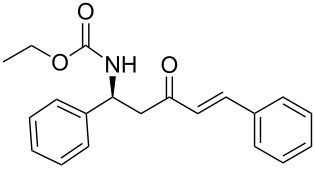 **15v**	84
23	**13e**	ethanal	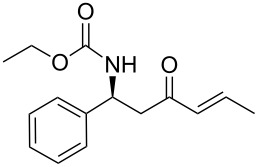 **15w**	80
24	**13e**	butanal	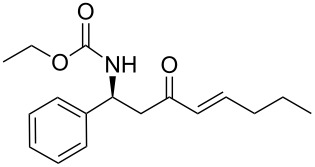 **15x**	95
25	**13e**	nonanal	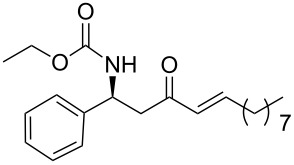 **15y**	89

26	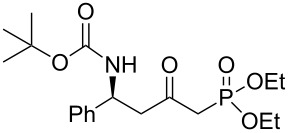 **13f**	ethanal	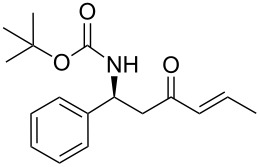 **15z**	81

27	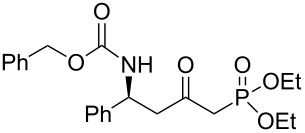 **13g**	ethanal	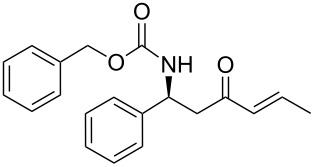 **1**	76

## Conclusion

In summary, a general methodology has been devised for the asymmetric synthesis of β’-amino-protected-α,β enones, a valuable intermediate for the synthesis of *trans* 2,6-disubstituted piperidines. The scope and limitation of the aza-Michael reaction were studied with a range of substrates. We are currently working on the application of this synthetic method to the preparation of piperidine natural products.

## Experimental

Organic solutions were dried over MgSO_4_ or Na_2_SO_4_, and filtered. When anhydrous solvents were used, they were prepared as follows: tetrahydrofuran (THF) was distilled under N_2_ from sodium benzophenone ketyl and used immediately; anhydrous acetonitrile was freshly distilled from CaH_2_. All ^1^H and ^13^C NMR spectra were measured in CDCl_3_ or C_6_D_6_ and recorded on a Bruker 400 MHz (101 MHz for ^13^C) spectrometer with TMS as the internal standard. Chemical shifts are expressed in parts per million (ppm) and *J*-values are given in hertz. The following abbreviations are used: singlet (s), doublet (d), doublet of doublets (dd), triplet (t), multiplet (m). High-resolution mass spectroscopy (HRMS) was carried out in electrospray mode and was performed by CRMP (Clermont-Ferrand, France). Monitoring of the reactions was performed by using silica-gel TLC plates (silica Merck 60 F254). Spots were visualized by UV light at 254 nm. Flash chromatography was performed by using silica gel 60 (70–230 mesh) or RP18 (25–40 lM) from Merck Chimie SAS (France) on a Flash II apparatus (Armen Instrument, France).

### General procedure for the synthesis of **10**

**(*R*)-Methyl 3-(ethoxycarbonylamino)butanoate 10a:** To a cold solution (0 °C) of (+)-(*R*)-*N*-benzyl-*N*-α-methylbenzylamine (23.0 mL, 110 mmol, 1.1 equiv) in dry THF (280 mL) was added *n*-butyllithium (75.0 mL, 1.6 M in hexane, 120 mmol, 1.2 equiv) slowly under argon. The resultant pink solution of lithium amide was stirred for 30 min then cooled to −78 °C before dropwise addition of a solution of methyl crotonate (10.0 mL, 100 mmol, 1 equiv) in dry THF (100 mL). The mixture was stirred at –78 °C for 90 min. Then, a saturated aqueous solution of NH_4_Cl (100 mL) was added slowly, and the resulting solution was allowed to warm to room temperature. Then, the solution was extracted twice with ethyl acetate. The combined organic extracts were dried over Na_2_SO_4_, filtered and evaporated. The crude product was added to a suspension of 10% Pd/C (5.00 g) in methanol (200 mL). The mixture was placed on a Parr apparatus and stirred under a hydrogen atmosphere (60 psi) for 4 days. The catalyst was removed by filtration on Celite^®^. The residue was concentrated in vacuum and dissolved in dichloromethane (200 mL) and water (200 mL). Then, sodium carbonate (42.4 g, 400 mmol, 4.0 equiv) and ethyl chloroformate (28.5 mL, 200 mmol, 2 equiv) were added dropwise. The resulting solution was stirred at room temperature for 3 h. The aqueous material was extracted with dichloromethane and the combined organic extracts were dried over Na_2_SO_4_, filtered and concentrated in vacuo. Purification by chromatography on silica gel (cyclohexane/EtOAc 9/1 to 5/5) afforded **10a** as a yellow oil (21.4 g, 57% in three steps): [α]_D_^25^ −35.6 (*c* 0.99, CHCl_3_), lit.[32] [α]_D_^25^ −37.07 (*c* 1, CHCl_3_); ^1^H NMR (400 MHz, CDCl_3_) δ 5.03 (br s, 1H, NH), 4.03 (m, 3H), 3.62 (s, 3H), 2.46 (d, *J* = 6.9 Hz, 2H), 1.16 (t, *J* = 6.9 Hz, 3H), 1.15 (d, *J* = 6.6 Hz, 3H). Spectral data are identical to those reported in [32].

### General procedure for the synthesis of **13**

**(*R*)-Ethyl [5-(diethoxyphosphoryl)-4-oxopentan-2-yl]carbamate 13a:** To a solution of diethyl methylphosphonate (5.8 mL, 39.7 mmol, 2.5 equiv) in anhydrous THF (15 mL) kept at −78 °C, was added dropwise *n*-butyl lithium (24.8 mL, 1.6 M in hexane, 39.7 mmol, 2.5 equiv). After 20 min at −78 °C, a solution of **10a** (3 g, 15.9 mmol, 1 equiv) in anhydrous THF (15 mL) was added dropwise. After addition, the temperature of the reaction was kept at −78 °C for 30 min and then allowed to reach 0 °C over 1 h, and the reaction was quenched with a solution of ammonium chloride and extracted twice with ethyl acetate. After drying over MgSO_4_ and concentration under vacuum, the crude oil was first distilled at low pressure to remove excess diethyl methylphosphonate, and the residue was then purified by flash chromatography (eluent: cyclohexane/EtOAc 2/1 to EtOAc) afforded **13a** as a yellow oil (3.3 g, 68% yield): [α]_D_^25^ +33.6 (*c* 1.17, CHCl_3_); ^1^H NMR (400 MHz, CDCl_3_) δ 5.03 (br s, 1H,), 4.16–3.94 (m, 7H), 3.08 (dd, *J* = 23.0, 14.0 Hz, 1H), 2.99 (dd, *J* = 22.6, 14.0 Hz, 1H), 2.84 (dd, *J* = 17.1, 6.0 Hz, 1H), 2.71 (dd, *J* = 17.1, 5.7 Hz, 1H), 1.33–1.21 (m, 6H), 1.15–1.20 (m, 6H); ^13^C NMR (101 MHz, CDCl_3_) δ 200.6, 155.8, 62.6 (d, *J* = 6.6 Hz), 62.5 (d, *J* = 6.5 Hz), 60.5, 49.6, 43.5, 42.9 (d, *J* = 127.4 Hz), 20.7, 16.2, 16.1, 14.6; HRMS-ESI (M + Na), *m/z* calcd. for C_12_H_24_NO_6_PNa 332.1239, found 332.1239.

### General procedure for the synthesis of **15**

**(*R*,*E*)-Ethyl [4-oxo-6-phenyl-hex-5-en-2-yl]carbamate 15a:** To a solution of **13a** (0.5 g, 1.6 mmol, 1 equiv) in THF (7 mL), was poured Ba(OH)_2_ (0.346 g, 2.0 mmol, 1.25 equiv) in one batch at room temperature. After 30 min, a solution of benzaldehyde (0.172 ml, 1.7 mmol, 1.05 equiv) in THF/H_2_O (40/1) (7 mL) was slowly added at room temperature. After 1 h, the reaction mixture was quenched with ammonium chloride and extracted three times with ethyl acetate. Then the organic layer was dried over MgSO_4_, concentrated under vacuum and purified by flash chromatography (eluent: cyclohexane to cyclohexane/EtOAc 8/2) to give **15a** as a white solid (0.401 g, 95%): Mp 74 °C; [α]_D_^25^ +9.5 (*c* 1.21, CHCl_3_); ^1^H NMR (400 MHz, CDCl_3_) δ 7.50 (d, *J* = 16.7 Hz, 1H), 7.47 (dd, *J* = 7.8, 3.0 Hz, 1H), 7.33–7.30 (m, 3H), 6.65 (d, *J* = 16.7 Hz, 1H), 5.14 (s, 1H), 4.14–4.06 (m, 1H), 4.02 (q, *J* = 6.9 Hz, 2H), 2.95 (dd, *J* = 15.9, 4.2 Hz, 1H), 2.71 (dd, *J* = 15.9, 6.5 Hz, 1H), 1.19 (d, *J* = 6.8 Hz, 3H), 1.14 (t, *J* = 6.9 Hz, 3H); ^13^C NMR (101 MHz, CDCl_3_) δ 198.8, 155.9, 143.4, 134.3, 130.6, 128.9, 128.4, 126.3, 60.6, 46.3, 44.1, 20.5, 14.6; HRMS-ESI (M + Na): calcd. for C_15_H_19_NO_3_Na 284.1263, found 284.1275.

## Supporting Information

File 1 Experimental section, characterization data and spectra of all new compounds.
