# Supplementary material for: Efficient synthesis of β’-amino-α,β-unsaturated ketones
Source: Beilstein J Org Chem. 2013 Mar 6;9:486–95. doi: 10.3762/bjoc.9.52 (PMC3596044; doi:10.3762/bjoc.9.52)
Supplement: File 1 — Experimental section, characterization data and spectra of all new compounds. [file Beilstein_J_Org_Chem-09-486-s001.pdf]

Supporting Information

for

Efficient synthesis of β'-amino-α,β-unsaturated ketones

Isabelle Abrunhosa-Thomas[1,2], Aurélie Plas[2,3], Nishanth Kandepedu[2,3], Pierre Chalard[1,2], Yves Troin*,§,1,2

Address:[1]Clermont Université, ENSCCF, Institut de Chimie de Clermont-Ferrand (ICCF), BP 10448, F-63000 CLERMONT-FERRAND, [2] CNRS, UMR 6296, Institut de Chimie de Clermont-Ferrand (ICCF), BP 80026, F-63171 AUBIERE and [3-]Clermont Université, Institut de Chimie de Clermont-Ferrand, BP 10448, F-63000 CLERMONT-FERRAND

[§]Fax: (33)473407008

Email: Yves Troin - yves.troin@ensccf.fr

*Corresponding author

Experimental section, characterization data and spectra of all new compounds

Table of contents:

1. Characterizations of β-*N*-protected aminoester **10** S2

2. Characterizations of phosphonates **13** S3

3. Characterizations of β-*N*-protected ketone **15** S4

4. References S10

4. ^{1}H NMR and ^{13}C NMR of products S11

1. Characterizations of β-*N*-protected aminoester 10

(*R*)-Methyl 3-(ethoxycarbonylamino)butanoate (**10a**).

Spectral data are identical with those reported [1]: $[\alpha]_D^{25}$ = −37.07 (*c* 1, $CHCl_3$). ^{1}H NMR (400 MHz, $CDCl_3$) δ 5.03 (br s, 1H), 4.03 (m, 3H), 3.62 (s, 3H), 2.46 (d, *J* = 6.9 Hz, 2H), 1.16 (t, *J* = 6.9 Hz, 3H), 1.15 (d, *J* = 6.6 Hz, 3H)

(*R*)-Methyl 3-(benzyloxycarbonylamino)butanoate (**10b**).

Spectral data are identical with those reported [2]: $[\alpha]_D^{25}$ = +16.9 (*c* 1.4, $CHCl_3$). ^{1}H NMR (400 MHz, $CDCl_3$) δ 5.03 (br s, 1H), 4.03 (m, 3H), 3.62 (s, 3H), 2.46 (d, *J* = 6.9 Hz, 2H), 1.16 (t, *J* = 6.9 Hz, 3H), 1.15 (d, *J* = 6.6, 3H).

(*R*)-Methyl 3-(benzyloxycarbonylamino)butanoate (**10c**) and (*S*)-methyl 3-(benzyloxycarbonylamino)butanoate (**10c'**).

R enantiomer: $[\alpha]_D^{25}$ = +41.5 (*c* 1.03, $CHCl_3$), *S* enantiomer: $[\alpha]_D^{25}$ = −40.9 (*c* 1.035, $CHCl_3$); ^{1}H NMR (400 MHz, $CDCl_3$) δ 4.98 (br s, 1H), 4.02 (m, *2*H), 3.90 (m, 1H), 3.61 (s, 3H), 2.49 (dd, *J* = 15.8, 4.8 Hz, 1H), 2.43 (dd, *J* = 15.8, 5.3 Hz, 1H), 1.48–1.22 (m, 2H), 1.16 (t, *J* = 7.0 Hz, 3H), 0.85 (t, *J* = 7.2 Hz, 3H); ^{13}C NMR (101 MHz, $CDCl_3$) δ 172,0, 156.0, 60.7, 51.6, 47.7, 38.9, 36.6, 19.3, 14.6, 13.8. HRMS-ESI (M + Na), *m/z*: calcd. for $C_{10}H_{19}NO_4Na$ 240.1212, found 240.1216.

(*R*)-Methyl 3-(ethoxycarbonylamino)undecanoate (**10d**).

$[\alpha]_D^{25}$ = +29.2 (*c* 1.05, $CHCl_3$); ^{1}H NMR (400 MHz, $CDCl_3$) δ 5.07 (br s, 1H), 4.22–4.03 (m, 2H), 3.98 (m, 1H), 3.69 (s, 3H), 2.57 (dd, *J* = 15.1, 4.6 Hz, 1H), 2.51 (dd, *J* = 15.1, 5.1, Hz 1H), 1.55–1.45 (m, 2H), 1.41–1.10 (m, 15H), 0.89 (t, *J* = 6.9 Hz, 3H); ^{13}C NMR (101 MHz, $CDCl_3$) δ 172.1, 156.1, 60.7, 51.5, 48.0, 38.9, 34.4, 31.8, 29.4, 29.3, 29.2, 26.1, 22.6, 14.6, 14.1; HRMS-ESI (M + Na), *m/z*: calcd. for $C_{15}H_{29}NO_4Na$ 310.1994, found 310.1996.

(*S*)-Methyl 3-(ethoxycarbonylamino)-3-phenylpropanoate (**10e**).

$[\alpha]_D^{25}$ = −9.7 (*c* 0.99, $CHCl_3$). ^{1}H NMR (400 MHz, $CDCl_3$) δ 7.36–7.25 (m, 5H), 5.75 (br s, 1H), 5.17 (m, 1H), 4.11 (q, *J* = 7.0 Hz, 2H), 3.62 (s, 3H), 2.91 (dd, *J* = 15.5, 6,0 Hz, 1H), 2.84 (dd, *J* = 15.5, 5.9 Hz, 1H), 1.23 (t, *J* = 7.0, 3H); ^{13}C NMR (101 MHz, $CDCl_3$) δ 171.3, 155.8, 140.9, 128.6, 127.6, 126.2, 61.0, 51.8, 51.7, 40.5, 14.6; HRMS-ESI (M + Na), *m/z*: calcd. for $C_{13}H_{17}NO_4Na$ 274.1055, found 274.1069.

(*S*)-Methyl 3-(*tert*-butoxycarbonylamino)-3-phenylpropanoate (**10f**).

Spectral data are identical with those reported [3]: $[\alpha]_D^{25}$ = −37.4 (*c* 1.2, $CHCl_3$). ^{1}H NMR (400 MHz, $CDCl_3$) δ 7.36–7.25 (m, 5H), 5.60 (br s, 1H), 3.62 (s, 3H), 2.84 (m, 2H), 1.42 (s, 9H); ^{13}C NMR (101 MHz, $CDCl_3$) δ 171.3, 155.0, 128.6, 127.5, 126.0, 111.7, 107.3, 100.3, 79.6, 51.6, 40.6, 28.4

(*S*)-Methyl 3-(benzyloxycarbonylamino)-3-phenylpropanoate (**10g**).

Spectral data are identical with those reported [4]: $[\alpha]_D^{25}$ = −16.1 (*c* 0.97, $CHCl_3$). ^1H NMR (400 MHz, $CDCl_3$) δ 7.22 (m, 10 H), 5.73 (br s, 1H), 5.09 (m, 1H), 5.01 (d, *J* = 12.3 Hz, 1H), 4.97 (d, *J* = 12.3 Hz, 1H), 3.50 (s, 3H), 2.81 (dd, *J* = 15.3, 5.0 Hz, 1H), 2.74 (dd, *J* = 15.3, 5.7 Hz, 1H).

2. Characterizations of phosphonates 13

(*R*)-Ethyl [5-(diethoxyphosphoryl)-4-oxopentan-2-yl]carbamate (**13a**).

$[\alpha]_D^{25}$ = +33.6 (*c* 1.17, $CHCl_3$); ^1H NMR (400 MHz, $CDCl_3$) δ 5.03 (br s, 1H), 4.16–3.94 (m, 7H), 3.08 (dd, *J* = 23.0, 14.0 Hz, 1H), 2.99 (dd, *J* = 22.6, 14.0 Hz, 1H), 2.84 (dd, *J* = 17.1, 6.0 Hz, 1H), 2.71 (dd, *J* = 17.1, 5.7 Hz, 1H), 1.33–1.21 (m, 6H), 1.15-1.20 (m, 6H); ^{13}C NMR (101 MHz, $CDCl_3$) δ 200.6, 155.8, 62.6 (d, *J* = 6.6 Hz), 62.5 (d, *J* = 6.5 Hz), 60.5, 49.6, 43.5, 42.9 (d, *J* = 127.4 Hz), 20.7, 16.2, 16.1, 14.6; HRMS-ESI (M + Na), *m/z*: calcd. for $C_{12}H_{24}NO_6PNa$ 332.1239, found 332.1239.

(*R*)-Benzyl [5-(diethoxyphosphoryl)-4-oxopentan-2-yl]carbamate (**13b**).

$[\alpha]_D^{25}$ = −26.4 (*c* 0.85, $CHCl_3$); ^1H NMR (400 MHz, $CDCl_3$) δ 7.36–7.22 (m, 5H), 5.26 (br s, 1H), 5.00 (s, 2H), 4.03 (m, 5H), 3.05 (dd, *J* = 23.2, 13.6 Hz, 1H), 2.96 (dd, *J* = 22.7, 13.6 Hz, 1H), 2.85 (dd, *J* = 17.3, 5.8 Hz, 1H), 2.70 (dd, *J* = 17.3, 5.6 Hz, 1H), 1.26 (t, *J* = 6.2 Hz, 6H), 1.16 (d, *J* = 6.7 Hz, 3H); ^{13}C NMR (101 MHz, $CDCl_3$) δ 200.7, 155.6, 136.6, 128.5, 128.0, 66.5, 62.7 (d, *J* = 6.6 Hz), 62.6 (d, *J* = 6.8 Hz), 49.5, 43.6, 43.3 (d, *J* = 129.5 Hz), 20.4, 16.3, 16.2; HRMS-ESI (M + Na), *m/z*: calcd. for $C_{17}H_{26}NO_6PNa$ 394.1395, found 394.1395.

(*R and S*)-Ethyl [1-(diethoxyphosphoryl)-2-oxoheptan-4-yl]carbamate (**13c** and **13c’**).

R enantiomer: $[\alpha]_D^{25}$ = +43.09 (*c* 1.03, $CHCl_3$), *S* enantiomer: $[\alpha]_D^{25}$ = −42.55 (*c* 1.075, $CHCl_3$). ^1H NMR (400 MHz, $CDCl_3$) δ 5.00 (d, *J* = 9.0 Hz, 1H), 4.15–3.97 (m, 6H), 3.90 (m, 1H), 3.09 (dd, *J* = 22.9, 13.5 Hz, 1H), 2.96 (dd, *J* = 22.5, 13.5 Hz, 1H), 2.80 (dd, *J* = 17.2, 5.9 Hz, 1H), 2.74 (dd, *J* = 17.2, 5.3 Hz, 1H), 1.48–1.39 (m, 2H), 1.37–1.29 (m, 2H), 1.27 (dt, *J* = 7.2, 2.0 Hz, 6H), 1.15 (t, *J* = 7.1 Hz, 3H), 0.84 (t, *J* = 7.2 Hz); ^{13}C NMR (101 MHz, $CDCl_3$) δ 200.9, 156.2, 62.7 (d, *J* = 6.5 Hz), 62.6 (d, *J* = 6.6 Hz), 60.6, 48.2, 47.5, 42.9 (d, *J* = 126.6 Hz), 36.7, 19.3, 16.3, 16.2, 14.6, 13.8; HRMS-ESI (M + Na), *m/z*: calcd. for $C_{14}H_{28}NO_6PNa$ 360.552, found 360.1562.

(*R*)-Ethyl [1-(diethoxyphosphoryl)-2-oxododecan-4-yl]carbamate (**13d**).

$[\alpha]_D^{25}$ = +30.06 (*c* 1.00, $CHCl_3$). ^1H NMR (400 MHz, $CDCl_3$) δ 5.02 (brs, 1H), 4.17–3.99 (m, 6H), 3.95 (m, 1H), 3.07 (dd, *J* = 23.1, 13.5 Hz, 1H), 2.98 (dd, *J* = 22.7, 13.5 Hz, 1H), 2.83 (dd, *J* = 17.4, 5.8 Hz, 1H), 2.75 (dd, *J* = 17.4, 5.2 Hz, 1H), 1.51–1.41 (m, 2H), 1.39–1.23 (m, 12H), 1.21 (t, *J* = 7.2 Hz, 6H), 1.16 (t, *J* = 7.1 Hz, 3H), 0.86 (t, *J* = 7.2 Hz, 3H); ^{13}C NMR (101 MHz, $CDCl_3$) δ 200.8, 156.1, 62.9 (d, *J* = 6.6 Hz), 62.8

(d, J = 6.7 Hz), 60.7, 48.2, 48.1, 42.7 (d, J = 127.1 Hz), 34.4, 31.8, 29.4, 29.3, 29.2, 26.1, 22.6, 16.4, 16.3, 14.6, 14.0; HRMS-ESI (M + Na), *m/z*: calcd. for $C_{19}H_{38}NO_6PNa$ 430.2334 found 430.2349.

(*S*)-Ethyl [4-(diethoxyphosphoryl)-3-oxo-1-phenylbutyl]carbamate (**13e**).

$[\alpha]_D^{25}$ = +1.65 (*c* 1.09, $CHCl_3$). ^1H NMR (400 MHz, $CDCl_3$) δ 7.29–7.21 (m, 4H), 7.16 (m, 1H), 5.72 (s, 1H), 5.09 (dd, J = 12.7, 6.7 Hz, 1H), 4.11–3.94 (m, 6H), 3.26 (dd, J = 16.9, 7.3 Hz, 1H), 3.04 (dd, J = 23.3, 13.1 Hz, 1H), 2.97 (dd, J = 16.9, 12.7 Hz, 1H), 2.93 (dd, J = 22.9, 13.1 Hz, 1H), 1.22 (td, J = 7.1, 1.9 Hz, 6H), 1.13 (t, J = 6.7 Hz, 3H); ^{13}C NMR (101 MHz, $CDCl_3$) δ 199.9, 155.9, 141.4, 128.6, 127.4, 126.3, 62.8 (d, J = 6.2 Hz), 62.6 (d, J = 6.5 Hz), 60.8, 51.1, 49.4, 43.3 (d, J = 125.5 Hz), 16.2 (d, J = 6.1 Hz), 14.6; HRMS-ESI (M + Na), *m/z*: calcd. for $C_{17}H_{26}NO_6PNa$ 394.1395, found 394.1414.

(*S*)-*tert*-Butyl [4-(diethoxyphosphoryl)-3-oxo-1-phenylbutyl]carbamate (**13f**).

$[\alpha]_D^{25}$ = +3.12 (*c* 1.01, $CHCl_3$); ^1H NMR (400 MHz, $CDCl_3$) δ 7.26–7.12 (m, 4H), 7.16 (m, 1H), 5.44 (br s, 1H), 5.03 (br s, 1H), 4.04–3.95 (m, 4H), 3.20 (dd, J = 16.8, 7.4 Hz, 1H), 3.01 (dd, J = 23.1, 12.9 Hz, 1H), 2.99 (m, 1H), 2.92 (dd, J = 22.7, 12.9 Hz, 1H), 1.32 (s, 9H), 1.21 (td, J = 7.1, 1.9 Hz, 6H); ^{13}C NMR (101 MHz, $CDCl_3$) δ 200.2, 155.1, 133.6, 130.9, 128.5, 126.3, 66.7, 62.8 (d, J = 6.3 Hz), 62.6 (d, J = 6.2 Hz), 50.9, 49.6, 41.0 (d, J = 127.6 Hz), 16.3 (d, J = 5.8 Hz), 16.2 (d, J = 5.6 Hz); HRMS-ESI (M + Na), *m/z*: calcd. for $C_{19}H_{30}NO_6PNa$ 422.1708, found 422.1722.

(*S*)-Benzyl [4-(diethoxyphosphoryl)-3-oxo-1-phenylbutyl]carbamate (**13g**).

$[\alpha]_D^{25}$ = +8.66 (*c* 1.55, $CHCl_3$). ^1H NMR (400 MHz, $CDCl_3$) δ 7.31–7.20 (m, 8H), 7.16 (m, 2H), 5.88 (br s, 1H), 5.11 (dd, J = 13.1, 7.4 Hz, 1H), 5.04 (d, J = 12.3 Hz, 1H), 4.96 (d, J = 12.3 Hz, 1H), 4.02–3.89 (m, 4H), 3.27 (dd, J = 16.6, 7.4 Hz, 1H), 3.02 (dd, J = 23.3, 13.1 Hz, 1H), 2.97 (m, 1H), 2.90 (dd, J = 22.6, 13.1 Hz, 1H), 1.21 (t, J = 7.0 Hz, 3H), 1.15 (t, J = 6.8 Hz, 3H); ^{13}C NMR (101 MHz, $CDCl_3$) δ 200.0, 155.6, 141.3, 136.5, 128.6, 128.4, 128.0, 127.5, 126.3, 66.7, 62.9 (d, J = 5.3 Hz), 62.6 (d, J = 6.4 Hz), 51.3, 49.3, 43.5 (d, J = 123.8 Hz), 16.3 (d, J = 4.1 Hz), 16.2 (d, J = 3.7 Hz); HRMS-ESI (M + Na), *m/z*: calcd. for $C_{22}H_{28}NO_6PNa$ 456.1552, found 456.1559.

3. Characterizations of β-*N*-protected ketone 15

(*R*,*E*)-Ethyl [4-oxo-6-phenylhex-5-en-2-yl]carbamate (**15a**).

$[\alpha]_D^{25}$ = +9.5 (*c* 1.21, $CHCl_3$). White solid: Mp 74 °C ; ^1H NMR (400 MHz, $CDCl_3$) δ 7.50 (d, J = 16.7 Hz, 1H), 7.47 (dd, J = 7.8, 3.0 Hz, 1H, 2H), 7.33–7.30 (m, 3H), 6.65 (d, J = 16.7 Hz, 1H), 5.14 (s, 1H), 4.14–4.06 (m, 1H), 4.02 (q, J = 6.9 Hz, 2H), 2.95 (dd, J = 15.9, 4.2 Hz, 1H), 2.71 (dd, J = 15.9, 6.5 Hz, 1H), 1.19 (d, J = 6.8 Hz, 3H), 1.14 (t, J = 6.9 Hz, 3H) ; ^{13}C NMR (101 MHz, $CDCl_3$) δ 198.8, 155.9, 143.4, 134.3,

130.6, 128.9, 128.4, 126.3, 60.6, 46.3, 44.1, 20.5, 14.6 ; HRMS-ESI (M + Na), *m/z*: calcd. for $C_{15}H_{19}NO_3Na$ 284.1263, found 284.1275.

(*R*,*E*)-Ethyl [6-(2-nitrophenyl)-4-oxohex-5-en-2-yl]carbamate (**15b**).

$[\alpha]_D^{25}$ = +21.94 (*c* 1.015, $CHCl_3$). Yellow solid: Mp 90 °C; ^1H NMR (400 MHz, $CDCl_3$) δ 8.07 (d, *J* = 8.1 Hz, 1H), 8.01 (d, *J* = 16.1 Hz, 1H), 7.71–7.59 (m, 2H), 7.56 (t, *J* = 7.4 Hz, 1H), 6.58 (d, *J* = 16.1 Hz, 1H), 5.08 (br s, 1H), 4.22–4.08 (m, 3H), 3.04 (dd, *J* = 16.3, 4.7 Hz, 1H), 2.85 (dd, *J* = 16.3, 6.3 Hz, 1H), 1.28 (d, *J* = 6.7 Hz, 3H), 1.22 (t, *J* = 7.0 Hz, 3H) ; ^{13}C NMR (101 MHz, $CDCl_3$) δ 198.3, 155.8, 148.3, 138.7, 133.7, 131.1, 130.8, 130.5, 129.1, 125.1, 60.7, 46.0, 43.9, 20.6, 14.6 ; HRMS-ESI (M + Na), *m/z*: calcd. for $C_{15}H_{18}N_2O_5Na$ 329.1113, found 329.1117.

(*R*,*E*)-Ethyl [6-(3-nitrophenyl)-4-oxohex-5-en-2-yl]carbamate (**15c**).

$[\alpha]_D^{25}$ = +5.92 (*c* 0.995, $CHCl_3$). Yellow solid: Mp 97 °C; ^1H NMR (400 MHz, $CDCl_3$) δ 8.43 (s, 1H), 8.27 (d, *J* = 8.1 Hz, 1H), 7.87 (d, *J* = 7.7 Hz, 1H), 7.65 (d, *J* = 16.2 Hz, 1H), 7.61 (d, *J* = 7.6 Hz, 1H), 6.85 (d, *J* = 16.2 Hz, 1H), 5.08 (br s, 1H), 4.27–4.04 (m, 3H), 3.07 (dd, *J* = 16.1, 3.3 Hz, 1H), 2.84 (dd, *J* = 16.1, 6.6 Hz, 1H), 1.31 (d, *J* = 6.7 Hz, 3H), 1.25 (t, *J* = 7.1 Hz, 3H) ; ^{13}C NMR (101 MHz, $CDCl_3$) δ 198.3, 155.6, 148.5, 140.2, 136.0, 133.9, 130.0, 128.6, 124.8, 122.6, 60.8, 47.0, 44.0, 20.5, 14.6 ; HRMS-ESI (M + Na), *m/z*: calcd. for $C_{15}H_{18}N_2O_5Na$ 329.1113, found 329.1125.

(*R*,*E*)-[Ethyl-6-(4-nitrophenyl)-4-oxohex-5-en-2-yl]carbamate (**15d**).

$[\alpha]_D^{25}$ = +16.42 (*c* 0.52, $CHCl_3$). Yellow solid: Mp 98 °C. ^1H NMR (400 MHz, $CDCl_3$) δ 8.19 (d, *J* = 8.8 Hz, 2H), 7.62 (d, *J* = 8.5 Hz, 2H), 7.53 (d, *J* = 16.2 Hz, 1H), 6.75 (d, *J* = 16.2 Hz, 1H), 4.97 (s, 1H), 4.10 (m, 1H), 4.03 (q, *J* = 7.0 Hz, 2H), 2.99 (dd, *J* = 15.6, 3.5 Hz, 1H), 2.74 (dd, *J* = 15.6, 6.5 Hz, 1H), 1.21 (d, *J* = 6.8 Hz, 1H), 1.15 (t, *J* = 7.0 Hz, 1H) ; ^{13}C NMR (101 MHz, $CDCl_3$) δ 198.0, 155.9, 140.5, 140.1, 128.5, 128.9, 124.8, 124.2, 60.8, 47.1, 44.0, 20.5, 14.6 ; HRMS-ESI (M + Na), *m/z*: calcd. for $C_{15}H_{18}N_2O_5Na$ 329.1113, found 329.1119.

(*R*,*E*)-Ethyl [6-(4-methoxyphenyl)-4-oxohex-5-en-2-yl]carbamate (**15e**).

$[\alpha]_D^{25}$ = +6.1 (*c* 1.055, $CHCl_3$). White solid: Mp 108 °C; ^1H NMR (400 MHz, $CDCl_3$) δ 7.54 (d, *J* = 16.1 Hz, 1H), 7.50 (d, *J* = 7.9 Hz, 2H), 6.91 (d, *J* = 7.9 Hz, 1H), 6.60 (d, *J* = 16.1 Hz, 1H), 5.21 (br s, 1H), 4.22–4.07 (m, 3H), 3.83 (s, 3H), 2.99 (dd, *J* = 15.8, 4.4 Hz, 1H), 2.76 (dd, *J* = 15.8, 5.8 Hz, 1H), 1.25 (d, *J* = 6.8 Hz, 1H), 1.21 (t, *J* = 7.0 Hz, 1H) ; ^{13}C NMR (101 MHz, $CDCl_3$) δ 198.8, 161.9, 155.9, 143.2, 130.3, 127.1, 124.3, 114.6, 60.8, 55.5, 46.2, 44.4, 20.6, 14.7 ; HRMS-ESI (M + Na), *m/z*: calcd. for $C_{16}H_{21}NO_4Na$ 314.1368, found 314.1371.

(*R*,*E*)-Ethyl [6-(2-bromophenyl)-4-oxohex-5-en-2-yl]carbamate (**15f**).

$[\alpha]_D^{25}$ = +18.2 (*c* 1.175, $CHCl_3$). White solid : Mp 65 °C. ^1H NMR (400 MHz, $CDCl_3$) δ 7.86 (d, *J* = 16.2 Hz, 1H), 7.56 (d, *J* = 7.8 Hz, 1H), 7.56 (d, *J* = 7.8 Hz, 1H), 7.27 (t, *J* = 7.8 Hz, 1H), 7.17 (td, *J* = 7.8, 1.5 Hz, 1H), 6.57 (d, *J* = 16.2 Hz, 1H), 5.08 (br s, 1H),

4.15–3.88 (m, 3H), 2.96 (dd, *J* = 16.4, 4.8 Hz, 1H), 2.79 (dd, *J* = 16.3, 6.2 Hz, 1H), 1.22 (d, *J* = 6.8 Hz, 3H), 1.16 (t, *J* = 7.1 Hz, 3H) ; ^{13}C NMR (101 MHz, $CDCl_3$) δ 198.8, 158.1, 145.0, 141.6, 133.5, 131.5, 129.0, 127.8, 60.7, 46.5, 44.0, 20.6, 14.6 ; HRMS-ESI (M + Na), *m/z*: calcd. for $C_{15}H_{18}BrNO_3Na$ 362.0368, found 362.0371.

(*R*,*E*)-Ethyl [6-(4-bromophenyl)-4-oxo-hex-5-en-2-yl]carbamate (**15g**).

$[\alpha]_D^{25}$ = +4.0 (*c* 1.03, $CHCl_3$). White solid : Mp 90 °C. ^{1}H NMR (400 MHz, $CDCl_3$) δ 7.53 (d, *J* = 8.6 Hz, 2H), 7.50 (d, *J* = 16.2 Hz, 1H), 7.41 (d, *J* = 8.5 Hz, 1H), 6.70 (d, *J* = 16.2 Hz, 1H), 5.10 (s, 1H, NH), 4.14 (m, 1H), 4.09 (q, *J* = 7.1 Hz, 1H), 3.01 (dd, *J* = 15.5, 3.4 Hz, 1H), 2.77 (dd, *J* = 15.5, 6.6 Hz, 1H), 1.27 (d, *J* = 6.8 Hz, 1H), 1.23 (t, *J* = 7.1, 1H) ; ^{13}C NMR (101 MHz, $CDCl_3$) δ 198.4, 156.0, 141.9, 133.2, 132.2, 129.7, 126.7, 124.9, 60.7, 50.0, 46.5, 44.1, 20.5, 14.6 ; HRMS-ESI (M + Na), *m/z*: calcd. for $C_{15}H_{18}BrNO_3Na$ 362.0368, found 362.0380.

(*R*,*E*)-Ethyl [6-(2-chloro-5-nitrophenyl)-4-oxo-hex-5-en-2-yl]carbamate (**15h**).

$[\alpha]_D^{25}$ = +15.06 (*c* 1.06, $CHCl_3$). Yellow solid : Mp 149 °C ; ^{1}H NMR (400 MHz, $CDCl_3$) δ 8.05 (d, *J* = 8.6 Hz, 1H), 7.97 (d, *J* = 16.0 Hz, 1H), 7.62 (s, 1H), 7.51 (d, *J* = 8.6, 1H), 6.57 (d, *J* = 16.0 Hz, 1H), 5.09 (br s, 1H), 4.23–4.03 (m, 3H), 3.05 (dd, *J* = 16.5, 5.2 Hz, 1H), 2.84 (dd, *J* = 16.5, 6.5 Hz, 1H), 1.28 (d, *J* = 6.9 Hz, 3H), 1.21 (t, *J* = 7.5 Hz, 3H) ; ^{13}C NMR (101 MHz, $CDCl_3$) δ 198.0, 158.9, 146.5, 140.4, 137.7, 132.9, 131.9, 130.5, 129.3, 126.7, 60.9, 46.5, 44.1, 20.7, 14.7 ; HRMS-ESI (M + Na), *m/z*: calcd. for $C_{15}H_{17}ClN_2O_5Na$ 363.0724, found 363.0717.

(*R*,*E*)-Ethyl [4-oxo-6-(pyridin-3-yl)-hex-5-en-2-yl]carbamate (**15i**).

$[\alpha]_D^{25}$ = +10.25 (*c* 0.865, $CHCl_3$). White solid: Mp 90 °C; ^{1}H NMR (400 MHz, $CDCl_3$) δ 8.71 (d, *J* = 1.6 Hz, 1H), 8.56 (d, *J* = 4.7 Hz, 1H), 7.82 (dt, *J* = 7.9,1.6, 1H), 7.51 (d, *J* = 16.3 Hz, 1H), 7.30 (dd, *J* = 7.9, 4.9 Hz, 1H), 6.72 (d, *J* = 16.3 Hz, 1H), 5.03 (br s, 1H), 4.20–3.70 (m, 3H), 2.98 (dd, *J* = 16.1, 4.0 Hz, 1H), 2.74 (dd, *J* = 16.1, 6.7 Hz, 1H), 1.21 (d, *J* = 6.9 Hz, 3H), 1.16 (t, *J* = 7.5 Hz, 3H) ; ^{13}C NMR (101 MHz, $CDCl_3$) δ 198.1, 155.9, 151.1, 149.9, 139.4, 134.5, 130.2, 128.0, 123.8, 60.7, 46.7, 44.1, 20.5, 14.6 ; HRMS-ESI (M + Na), *m/z*: calcd. for $C_{14}H_{18}N_2O_3Na$ 285.1215, found 285.1210.

(*R*,2*E*,4*E*)-Ethyl 8-(ethoxycarbonylamino)-6-oxo-nona-2,4-dienoate (**15j**).

$[\alpha]_D^{25}$ = +17.6 (*c* 0.695, $CHCl_3$). Viscous yellow oil. ^{1}H NMR (400 MHz, $CDCl_3$) δ 7.24 (dd, *J* = 14.9, 11.4 Hz, 1H), 7.14 (dd, *J* = 15.1, 11.4 Hz, 1H), 6.35 (d, *J* = 15.0 Hz, 1H), 6.19 (d, *J* = 15.0 Hz, 1H), 4.97 (s, 1H), 4.17 (q, *J* = 7.1 Hz, 2H), 4.05–4.00 (m, 3H), 2.89 (d, *J* = 12.5 Hz, 1H), 2.67 (dd, *J* = 16.3, 6.5 Hz, 1H), 1.25 (t, *J* = 7.1 Hz, 3H), 1.18 (d, *J* = 6.8 Hz, 3H), 1.16 (t, *J* = 7.1 Hz, 3H) ; ^{13}C NMR (101 MHz, $CDCl_3$) δ 198.4, 165.7, 155.8, 141.1, 139.1, 135.3, 129.5, 60.9, 60.7, 46.7, 43.9, 20.4, 14.6, 14.2 ; HRMS-ESI (M + Na), *m/z*: calcd. for $C_{14}H_{21}NO_5Na$ 306.1317, found 306.1331.

(R)-Ethyl 6-(ethoxycarbonylamino)-4-oxohept-2-enoate (**15k**).

Mixture of Z and E isomers (Z/E : 60/40): Colorless oil. ^{1}H NMR (400 MHz, $CDCl_3$) δ 6.96 (d, J = 16.0 Hz, 1H), 6.62 (d, J = 16.0 Hz, 1H), 6.43 (d, J = 12.0 Hz, 1H), 5.97 (d, J = 12.0 Hz, 1H), 5.18 (s, 1H), 5.12 (s, 1H), 4.25–4.11 (m, 6H), 2.91 (d, J = 15.0 Hz, 1H), 2.81 (dd, J = 16.9, 5.7 Hz, 1H), 2.78–2.67 (m, 1H), 1.27–1.13 (m, 12H) ; ^{13}C NMR (101 MHz, $CDCl_3$) (mixture of Z and E) δ 201.1, 197.3, 164.4, 164.2, 155.7, 155.0, 140.6, 138.3, 130.4, 124.0, 60.5, 60.3, 59.8, 59.7, 47.3, 46.0, 42.7, 42.4, 19.6, 19.4, 13.6, 13.1, 13.0 ; HRMS-ESI (M + Na), m/z: calcd. for $C_{12}H_{19}NO_5Na$ 280.1161, found 280.1163.

(R,E)-Ethyl [4-oxo-non-5-en-2-yl]carbamate (**15l**).

$[\alpha]_D^{25}$ = +12.13 (c 1.025, $CHCl_3$). Colorless oil. ^{1}H NMR (400 MHz, $CDCl_3$) δ 6.84 (dt, J = 15.9, 7.2 Hz, 1H), 6.07 (dd, J = 15.9, 1.5 Hz, 1H), 5.13 (br s, 1H), 4.11–4.02 (m, 3H), 2.87 (dd, J = 16.1, 4.4 Hz, 1H), 2.65 (dd, J = 16.1, 6.4 Hz, 1H), 2.19 (td, J = 7.2, 1.5 Hz, 2H), 1.49 (qd, J = 7.2 Hz, 2H), 1.22 (t, J = 7.2 Hz, 3H), 1.21 (d, J = 6.8 Hz, 3H), 0.92 (t, J = 7.1 Hz, 3H) ; ^{13}C NMR (101 MHz, $CDCl_3$) δ 199.1, 156.0, 148.5, 130.9, 60.7, 45.5, 44.2, 34.6, 21.4, 20.6, 14.7, 13.8 ; HRMS-ESI (M + Na), m/z: calcd. for $C_{12}H_{21}NO_3Na$ 250.1419, found 250.1421.

(R,E)-Ethyl [4-oxo-tetradec-5-en-2-yl]carbamate (**15m**).

$[\alpha]_D^{25}$ = +10.71 (c 1.025, $CHCl_3$). Yellow oil. ^{1}H NMR (400 MHz, $CDCl_3$) δ 6.79 (dt, J = 15.8, 6.9 Hz, 1H), 6.01 (d, J = 15.8 Hz, 1H), 5.16 (s, 1H), 4.07–4.03 (m, 1H, 3H), 2.86 (dd, J = 16.0, 4.0 Hz, 1H), 2.63 (dd, J = 16.0, 6.4 Hz, 1H), 2.14 (q, J = 6.9 Hz, 2H), 1.45–1.32 (m, 2H), 1.30–1.18 (m, 15H), 1.15 (d, J = 7.1 Hz, 3H), 0.81 (t, J = 6.9 Hz, 3H) ; ^{13}C NMR (101 MHz, $CDCl_3$) δ 199.1, 155.9, 148.7, 130.7, 60.7, 45.5, 44.1, 32.6, 31.9, 29.5, 29.4, 29.3, 29.2, 28.1, 22.7, 20.5, 14.7, 14.2 ; HRMS-ESI (M + Na), m/z: calcd. for $C_{17}H_{31}NO_3Na$ 320.2202, found 320.2209.

(R,E)-Benzyl [4-oxo-6-phenyl-hex-5-en-2-yl]carbamate (**15n**).

$[\alpha]_D^{25}$ = +2.56 (c 1.95, $CHCl_3$). White solid : Mp 96 °C ; ^{1}H NMR (400 MHz, $CDCl_3$) δ 7.50 (d, J = 16.1 Hz, 1H), 7.48–7.46 (m, 2H), 7.33–7.19 (m, 8H), 6.64 (d, J = 16.1 Hz, 1H), 5.23 (s, 1H), 5.10–4.96 (m, 2H), 4.18–4.06 (m, 1H), 2.97 (d, J = 15.7 Hz, 1H), 2.73 (dd, J = 15.7, 5.6 Hz, 1H), 1.21 (d, J = 6.2 Hz, 3H) ; ^{13}C NMR (101 MHz, $CDCl_3$) δ 199.5, 155.6, 143.1, 134.0, 130.7, 129.0, 128.5, 128.4, 128.1, 126.3, 66.6, 46.1, 44.3, 20.5 ; HRMS-ESI (M + Na), m/z: calcd. for $C_{20}H_{21}NO_3Na$ 346.1419, found 346.1424.

(R,E)-Benzyl [4-oxo-pentadec-5-en-2-yl]carbamate (**15o**).

$[\alpha]_D^{25}$ = -9.86 (c 0.975, $CHCl_3$). Yellow oil. ^{1}H NMR (400 MHz, $CDCl_3$) δ 7.42–7.30 (m, 5H), 6.87 (dt, J = 16.0, 6.7 Hz, 1H), 6.09 (d, J = 16.0 Hz, 1H), 5.29 (br s, 1H), 5.10 (br s, 2H), 4.13 (m, 1H), 2.91 (dd, J = 16.1, 3.1 Hz, 1H), 2.69 (dd, J = 16.1, 6.1 Hz, 1H), 2.22 (dt, J = 6.7, 7.1 Hz, 2H), 1.52–1.41 (m, 2H), 1.36–1.27 (m, 12H), 1.25 (d, J = 6.8 Hz, 3H), 0.90 (t, J = 7.1 Hz, 3H) ; ^{13}C NMR (101 MHz, $CDCl_3$) δ 200.4, 155.6, 148.7,

139.5, 130.5, 128.5, 128.1, 128.0, 66.5, 45.2, 44.2, 32.5, 31.8, 29.5, 29.4, 29.3, 29.2, 28.04, 22.6, 20.4, 14.1 ; HRMS-ESI (M + Na), *m/z*: calcd. for $C_{23}H_{36}NO_3$ 374.2695, found 374.2706.

(*R*,*E*)-Benzyl [4-oxo-heptadec-5-en-2-yl]carbamate (**15p**).

$[\alpha]_D^{25}$ = -9.80 (*c* 1.015, $CHCl_3$). Yellow oil. ^{1}H NMR (400 MHz, $CDCl_3$) δ 7.42–7.30 (m, 5H), 6.87 (dt, *J* = 15.9, 7.2 Hz, 1H), 6.06 (d, *J* = 15.9 Hz, 1H), 5.31 (br s, 1H), 5.08 (br s, 2H), 4.10 (m, 1H), 2.89 (d, *J* = 15.6 Hz, 1H), 2.66 (dd, *J* = 15.6, 5.3 Hz, 1H), 2.19 (q, *J* = 7.2 Hz, 2H), 1.47–1.40 (m, 6H), 1.33–1.21 (m, 15H), 0.88 (t, *J* = 6.9 Hz, 3H); ^{13}C NMR (101 MHz, $CDCl_3$) δ 199.1, 155.7, 148.9, 136.7, 130.6, 128.6, 128.1, 66.6, 45.3, 44.3, 32.6, 32.0, 29.7, 29.6, 29.5, 29.4, 29.3, 28.1, 27.0, 22.8, 20.5, 14.2; HRMS-ESI (M + Na), *m/z*: calcd. for $C_{25}H_{40}NO_3$ 402.3008, found 402.3015.

(*E*)-Ethyl [6-oxo-8-phenyl-oct-7-en-4-yl]carbamate (**15q** and **15q’**).

R enantiomer: $[\alpha]_D^{25}$ = +21.87 (*c* 0.97, $CHCl_3$), *S* enantiomer: $[\alpha]_D^{25}$ = −21.32 (c 0.76 $CHCl_3$). White solid: Mp 96 °C; ^{1}H NMR (400 MHz, $CDCl_3$) δ 7.48 (d, *J* = 16.8 Hz, 1H), 7.45–7.3 (m, 2H), 7.33–7.30 (m, 3H), 6.64 (d, *J* = 16.8 Hz, 1H), 5.06 (br s, 1H), 4.09–3.91 (m, 3H), 2.90 (dd, *J* = 17.2, 6.0 Hz, 1H), 2.78 (dd, *J* = 17.2, 5.5 Hz, 1H), 1.62–1.29 (m, 4H), 1.17 (t, *J* = 6.9 Hz, 3H), 0.85 (t, *J* = 7.3 Hz, 3H); ^{13}C NMR (101 MHz, $CDCl_3$) δ 199.0, 156.2, 143.2, 134.3, 130.6, 128.9, 128.4, 126.3, 60.6, 48.1, 44.8, 36.5, 19.5, 14.6, 13.8; HRMS-ESI (M + Na), *m/z*: calcd. for $C_{17}H_{23}NO_3Na$ 312.1576, found 312.1585.

(*R*,*E*)-Ethyl 8-(4-nitrophenyl)-6-oxo-oct-7-en-4-yl]carbamate (**15r**).

$[\alpha]_D^{25}$ = +13.8 (*c* 0.985, $CHCl_3$). Yellow solid : Mp 102 °C; ^{1}H NMR (400 MHz, $CDCl_3$) δ 8.25 (d, *J* = 8.1 Hz, 2H), 7.70 (d, *J* = 8.1 Hz, 2H), 7.60 (d, *J* = 16.2 Hz, 1H), 6.83 (d, *J* = 16.2 Hz, 1H), 5.01 (s, 1H), 4.14–4.01 (m, 3H), 3.01 (d, *J* = 16.2 Hz, 1H), 2.85 (dd, *J* = 16.2, 5.7 Hz, 1H), 1.64–1.49 (m, 2H), 1.47–1.30 (m, 2H), 1.22 (t, *J* = 6.8 Hz, 3H), 0.92 (t, *J* = 7.2 Hz, 1H); ^{13}C NMR (101 MHz, $CDCl_3$) δ 198.5, 156.2, 140.6, 140.0, 129.5, 128.9, 124.2, 124.2, 60.8, 48.1, 45.8, 36.6, 19.5, 14.6, 13.8; HRMS-ESI (M + Na), *m/z*: calcd. for $C_{17}H_{22}N_2O_5Na$ 357.1426, found 357.1420.

(*R*,*E*)-Ethyl [3-oxo-1-phenyl-tridec-1-en-5-yl]carbamate (**15s**).

$[\alpha]_D^{25}$ = +17.26 (*c* 1.015, $CHCl_3$). White solid : Mp 76 °C; ^{1}H NMR (400 MHz, $CDCl_3$) δ 7.58 (d, *J* = 16.1 Hz, 1H), 7.57–7.52 (m, 2H), 7.44–7.36 (m, 3H), 6.74 (d, *J* = 16.1 Hz, 1H), 5.23 (d, *J* = 7.4 Hz, 1H), 4.21–3.94 (m, 3H), 3.01 (d, *J* = 15.2 Hz, 1H), 2.84 (dd, *J* = 15.2, 3.8 Hz, 1H), 1.69–1.49 (m, 2H), 1.48–1.11 (m, 15H), 0.85 (t, *J* = 6.8 Hz, 3H) ; ^{13}C NMR (101 MHz, $CDCl_3$) δ 199.0, 156.2, 143.2, 134.3, 130.6, 129.6, 128.9, 128.4, 126.3, 60.6, 48.4, 44.9, 34.4, 31.8, 29.5, 29.2, 26.3, 22.6, 14.6, 14.1 ; HRMS-ESI (M + Na), *m/z*: calcd. for $C_{22}H_{33}NO_3Na$ 382.2358, found 382.2364.

(R,E)-Ethyl [4-oxo-tetradec-2-en-6-yl]carbamate (**15t**).

$[\alpha]_D^{25}$ = +17.44 (c 1.12, $CHCl_3$). White solid : Mp 59 °C ; ^1H NMR (400 MHz, $CDCl_3$) δ 6.87 (dt, J = 15.8, 6.8, 1H), 6.10 (d, J = 15.8, 1.6 Hz, 1H), 5.1 (br s, 1H), 4.07 (q, J = 7.2 Hz, 2H), 3.91 (m, 1H), 2.84 (dd, J = 15.8, 4.2 Hz, 1H), 2.67 (dd, J = 15.8, 5.5 Hz, 1H), 1.90 (d, 3H, J = 6.8 Hz), 1.55–1.46 (m, 2H), 1.33–1.20 (m, 15H), 0.86 (t, J = 6.9 Hz, 3H) ; ^{13}C NMR (101 MHz, $CDCl_3$) δ 199.2, 156.3, 143.6, 132.4, 60.7, 48.5, 44.0, 34.5, 31.9, 29.6, 29.5, 29.4, 26.4, 22.8, 18.5, 14.7, 14.2 ; HRMS-ESI (M + Na), m/z: calcd. for $C_{17}H_{31}NO_3Na$ 320.2202, found 320.2207.

(R,E)-Ethyl [6-oxo-hexadec-4-en-8-yl]carbamate (**15u**).

$[\alpha]_D^{25}$ = +12.1 (c 1.05, $CHCl_3$). Colorless oil. ^1H NMR (400 MHz, $CDCl_3$) δ 6.83 (dt, J = 15.9, 7.2 Hz, 1H), 6.07 (dd, J = 15.9, 1.4 Hz, 1H), 5.11 (br s, 1H), 4.07 (qd, J = 6.9, 2H), 3.91 (m, 1H), 2.85 (dd, J = 16.3, 4.5 Hz, 1H), 2.69 (dd, J = 16.3, 5.7 Hz, 1H), 2.18 (dd, J = 7.2, 1.4 Hz, 1H), 1.53–1.44 (m, 2H), 1.49 (qd, J = 7.2 Hz, 2H), 1. 29–1.19 (m, 15H), 0.92 (t, J = 7.2 Hz, 3H), 0.86 (t, J = 7.1 Hz, 3H); ^{13}C NMR (101 MHz, $CDCl_3$) δ 199.4, 156.3, 148.3, 130.9, 60.7, 46.5, 44.1, 34.6, 34.5, 31.9, 29.6, 29.5, 29.4, 26.4, 22.8, 21.4, 14.7, 14.2, 13.8; HRMS-ESI (M + Na), m/z: calcd. for $C_{19}H_{35}NO_3Na$ 348.2515, found 348.2525.

(S,E)-Ethyl [3-oxo-1,5-diphenyl-pent-4-enyl]carbamate (**15v**).

$[\alpha]_D^{25}$ = +6.6 (c 0.94, $CHCl_3$). Yellow oil. ^1H NMR (400 MHz, $CDCl_3$) δ 7.43 (d, J = 16.2 Hz, 1H), 7.41–7.11 (m, 10H), 6.59 (d, J = 16.2 Hz, 1H), 5.74 (br s, 1H), 5.16 (m, 1H), 4.01 (q, J = 6.3 Hz, 1H), 3.26 (dd, J = 15.9 Hz, 1H), 3.06 (dd, J = 15.9, 5.0 Hz, 1H), 1.13 (t, J = 6.3 Hz, 3H); ^{13}C NMR (101 MHz, $CDCl_3$) δ 197.9, 155.9, 143.6, 134.2, 130.7, 128.9, 128.6, 128.4, 127.5, 126.3, 126.0, 61.0, 51.7, 46.1, 14.6; HRMS-ESI (M + Na), m/z: calcd. for $C_{20}H_{21}NO_3Na$ 346.1419, found 346.1414.

(S,E)-Ethyl [3-oxo-1-phenyl-hex-4-enyl]carbamate (15w).

$[\alpha]_D^{25}$ = −13.3 (c 0.715, $CHCl_3$). Yellow oil. ^1H NMR (400 MHz, $CDCl_3$) δ 7.32–7.12 (m, 5H), 6.75 (dq, J = 15.9, 6.8 Hz, 1H), 5.96 (dd, J = 15.9,1.5 Hz, 1H), 5.68 (br s, 1H), 5.09 (m, 1H), 4.01 (q, J = 6.9 Hz, 2H), 3.12 (dd, J = 16.6, 5.8 Hz, 1H), 2.93 (dd, J = 16.6, 6.8 Hz, 1H), 1.79 (dd, J = 6.8, 1.5 Hz, 3H), 1.10 (t, J = 6.9 Hz, 3H); ^{13}C NMR (101 MHz, $CDCl_3$) δ 200.2, 158.2, 146.2, 143.7, 134.2, 130.8, 129.6, 128.5, 63.2, 53.9, 45.5, 20.6, 16.8; HRMS-ESI (M + Na), m/z: calcd. for $C_{15}H_{19}NO_3Na$ 284.1263, found 284.1275.

(S,E)-Ethyl [3-oxo-1-phenyl-oct-4-enyl]carbamate (15x).

$[\alpha]_D^{25}$ = −9.11 (c 1.14, $CHCl_3$). Colorless oil. ^1H NMR (400 MHz, $CDCl_3$) δ 7.35-7.21 (m, 5H), 6.80 (dt, J = 15.9, 6.9 Hz, 1H), 6.05 (td, J = 15.9, 1.5, 1H), 5.75 (s, 1H), 5.15 (dd, J = 5.8,3.1 Hz, 1H), 4.11 (q, J = 7.1 Hz, 2H), 3.23 (dd, J = 16.1, 3.1 Hz, 1H), 3.04 (dd, J = 16.1, 5.8 Hz, 1H), 2.16 (qd, J = 6.9, 1.5 Hz, 2H), 1.47 (sex, J = 6.8 Hz, 2H), 1.23 (t, J = 7.1 Hz, 3H), 0.92 (t, J = 6.8 Hz, 3H); ^{13}C NMR (101 MHz, $CDCl_3$) δ 198.3,

156.1, 148.8, 141.6, 130.6, 128.6, 127.4, 126.4, 61.0, 51.8, 45.3, 34.6, 21.3, 14.6, 13.7; HRMS-ESI (M + Na), *m/z*: calcd. for $C_{17}H_{23}NO_3Na$ 312.1576, found 312.1576.

(*S*,*E*)-Ethyl [3-oxo-1-phenyl-tridec-4-enyl]carbamate (**15y**).

$[\alpha]_D^{25}$ = −1.22 (*c* 0.995, $CHCl_3$). White solid: Mp 60 °C; ^{1}H NMR (400 MHz, $CDCl_3$) δ 7.23–7.17 (m, 4H), 7.13 (m, 1H), 6.70 (dt, *J* = 15.9, 6.9 Hz, 1H), 5.93 (d, *J* = 15.9 Hz, 1H), 5.88 (br s, 1H), 5.07 (m, 1H), 3.98 (q, *J* = 7.1 Hz, 2H), 3.09 (d, *J* = 16.1 Hz, 1H), 2.89 (dd, *J* = 16.1, 5.7 Hz, 1H), 2.06 (q, *J* = 6.9 Hz, 2H), 1.39–1.29 (m, 2H), 1.27–1.13 (m, 10H), 1.10 (t, *J* = 7.1 Hz, 3H), 0.79 (t, *J* = 6.9 Hz, 3H); ^{13}C NMR (101 MHz, $CDCl_3$) δ 198.2, 156.0, 148.9, 141.7, 130.3, 128.5, 127.3, 126.3, 60.8, 51.6, 45.3, 32.5, 31.8, 29.4, 29.3, 29.2, 29.1, 27.9, 22.6, 14.5, 14.1; HRMS-ESI (M + Na), *m/z*: calcd. for $C_{22}H_{33}NO_3Na$ 382.2358, found 382.2362.

(*S*,*E*)-*tert*-Butyl [3-oxo-1-phenyl-hex-4-enyl]carbamate (**15z**).

$[\alpha]_D^{25}$ = −10.68 (*c* 1.015, $CHCl_3$). White solid: Mp 94 °C; ^{1}H NMR (400 MHz, $CDCl_3$) δ 7.32–7.18 (m, 5H), 6.84 (dq, *J* = 15.9, 6.8 Hz, 1H), 6.06 (dd, *J* = 15.9, 1.6 Hz, 1H), 5.55 (br s, 1H), 5.09 (m, 1H), 3.12 (d, *J* = 16.6 Hz, 1H), 2.98 (dd, *J* = 16.6, 5.6 Hz, 1H), 1.85 (dd, *J* = 6.8, 1.6 Hz, 3H), 1.40 (s, 9H); ^{13}C NMR (101 MHz, $CDCl_3$) δ 198.2, 155.3, 144.0, 132.1, 128.7, 127.4, 126.4, 51.5, 45.6, 28.5, 18.5; HRMS-ESI (M + Na), *m/z*: calcd. for $C_{17}H_{23}NO_3Na$ 312.1576, found 312.1591.

(*S*,*E*)-Benzyl [3-oxo-1-phenyl-hex-4-enyl]carbamate (**1)**.

$[\alpha]_D^{25}$ = −5.34 (*c* 1.05, $CHCl_3$). White solid: Mp 60 °C; ^{1}H NMR (400 MHz, $CDCl_3$) δ 7.42–7.17 (m, 5H), 6.80 (dq, *J* = 15.8, 6.8 Hz, 1H), 6.05 (d, *J* = 15.8 Hz, 1H), 5.88 (br s, 1H), 5.18 (dd, *J* = 6.2, 5.6 Hz, 1H), 5.10 (d, *J* = 12.3 Hz, 1H), 5.05 (d, *J* = 12.3 Hz, 1H), 3.19 (dd, *J* = 16.2, 5.6 Hz, 1H), 3.00 (dd, *J* = 16.2, 6.2 Hz, 1H), 1.85 (d, *J* = 6.8 Hz, 1H); ^{13}C NMR (101 MHz, $CDCl_3$) δ 197.8, 155.7, 144.0, 136.4, 136.2, 131.9, 128.7, 128.6, 128.5, 128.0, 126.4, 126.3, 66.8, 51.8, 45.1, 18.3; HRMS-ESI (M + Na), *m/z*: calcd. for $C_{17}H_{23}NO_3Na$ 346.1419, found 346.1426.

4. References

1. Cooper, J.; Knight, D.W.; Gallagher, P.T. *J. Chem. Soc.: Perkin Trans. 1* **1991**, 705-713.
2. Fleck, T. J.; McWhorter, W. W.; Dekam, R. N.; Pearlman, B. A. *J. Org. Chem.* **2003**, *68*, 9612-9617.
3. Hansen, K. B.; Rosner, T.; Kubryk, M.; Dormer, P. G.; Armstrong, J. D. III. *Org. Lett.* **2005**, *7*, 4935-4938.
4. Tillman, A. L.; Ye, J.; Dixon, D. J. *Chem. Commun.* **2006**, 1191-1193.

4. ^{1}H NMR and ^{13}C NMR of products

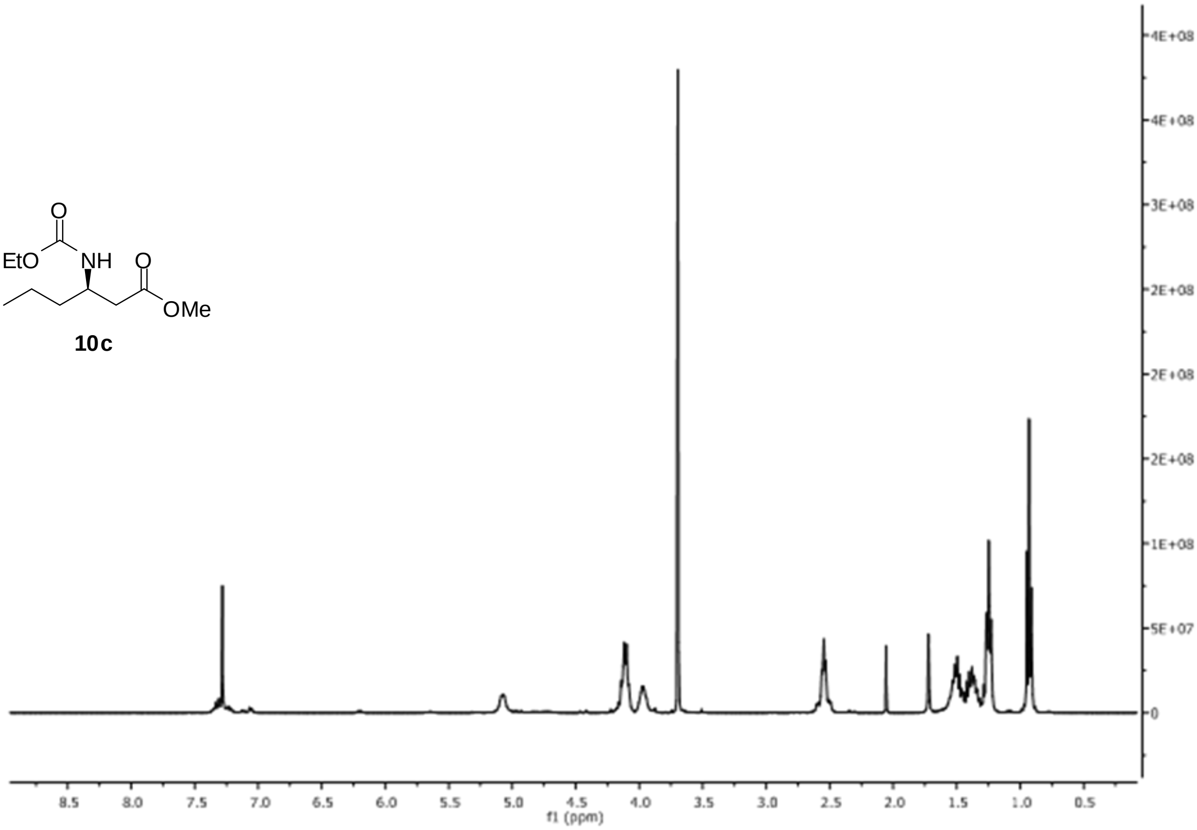

10c

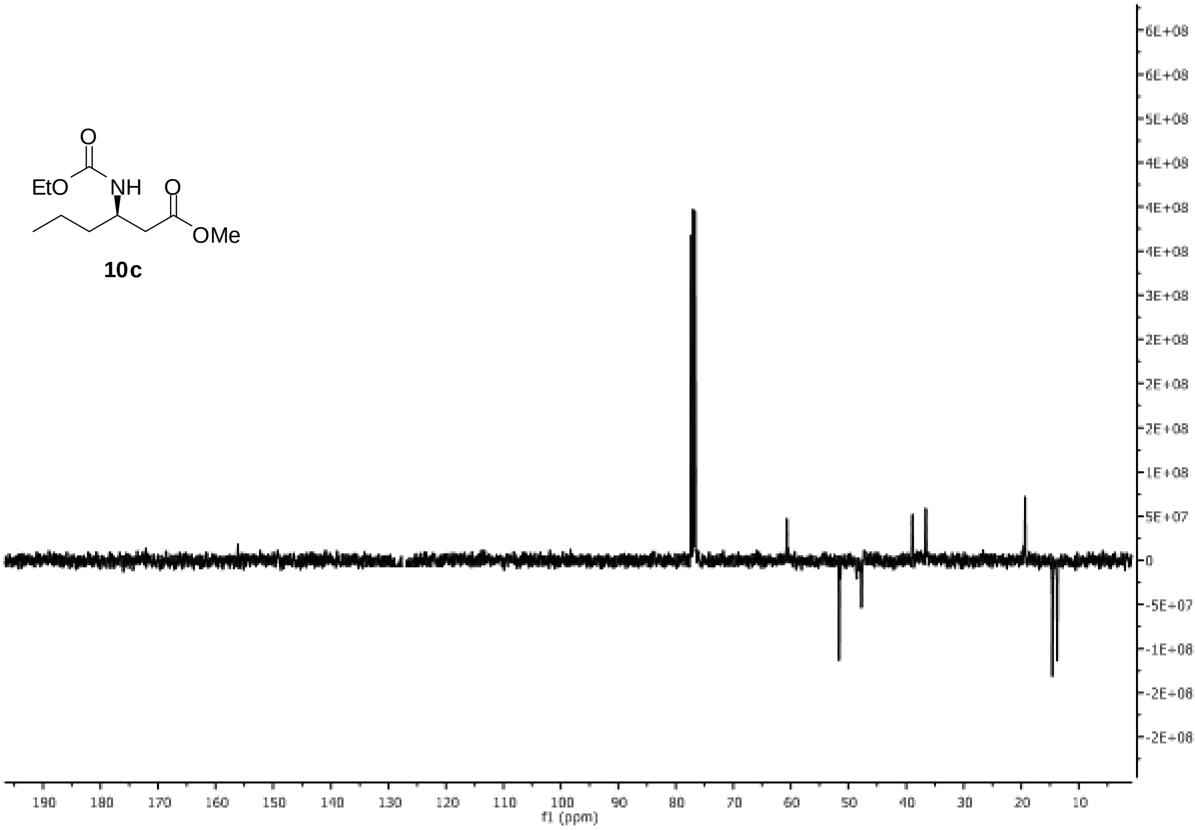

10c

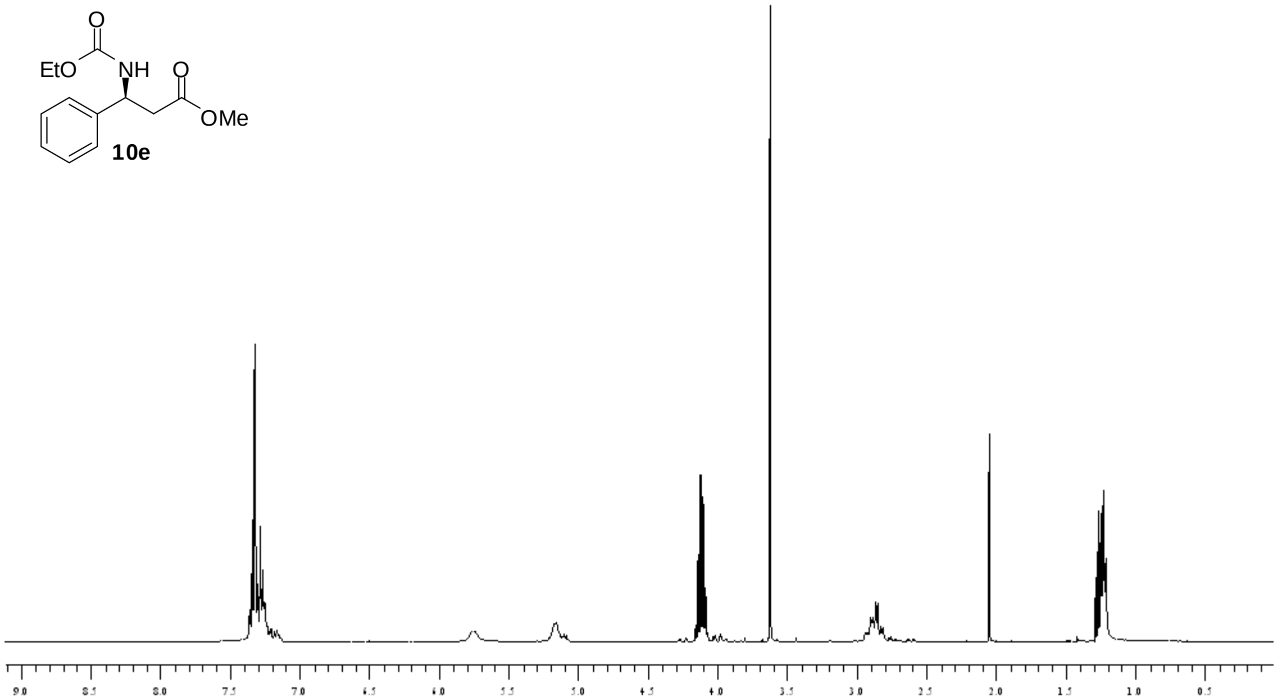
O
EtO
NH
O
OMe
10e
9.0
8.5
8.0
7.5
7.0
6.5
6.0
5.5
5.0
4.5
4.0
3.5
3.0
2.5
2.0
1.5
1.0
0.5

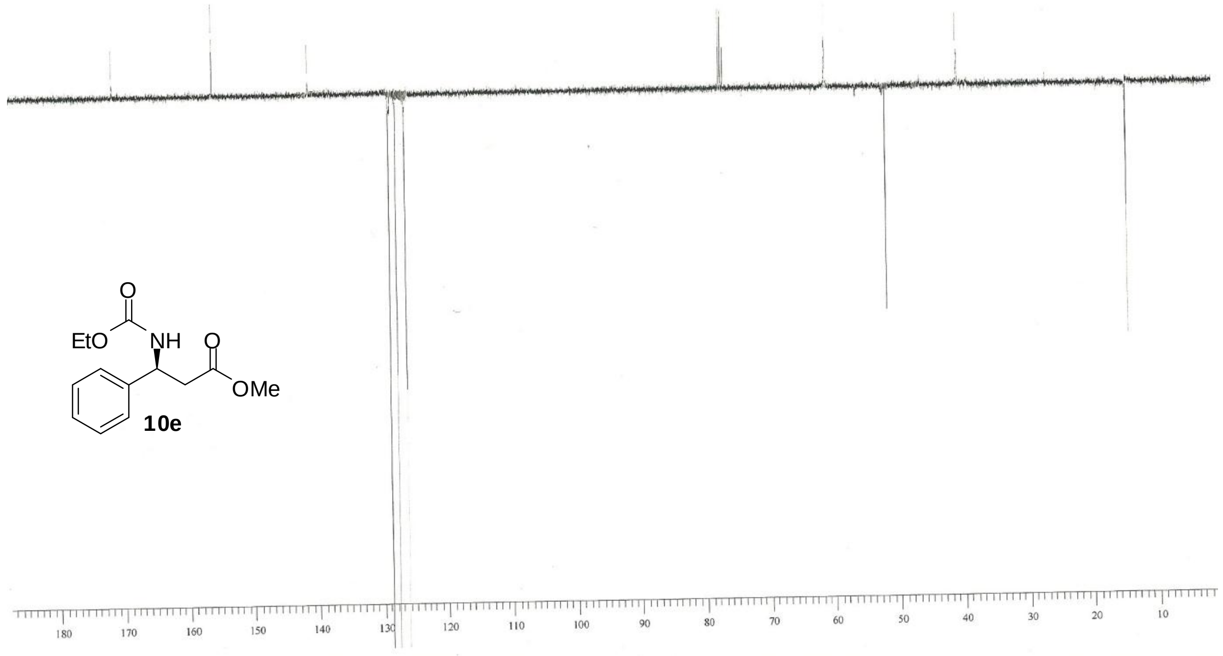
O
EtO
NH
O
OMe
10e
180
170
160
150
140
130
120
110
100
90
80
70
60
50
40
30
20
10

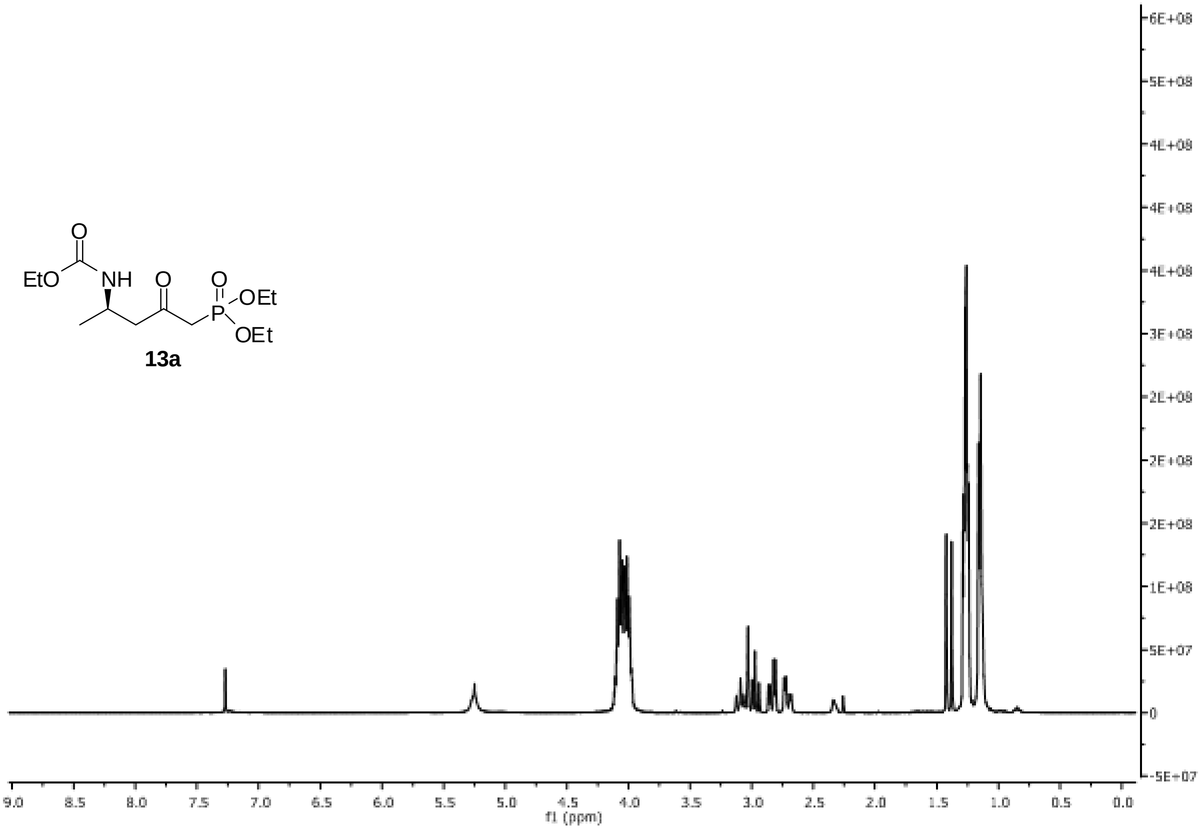

EtO
NH
O
O
P
OEt
OEt
13a
f1 (ppm)

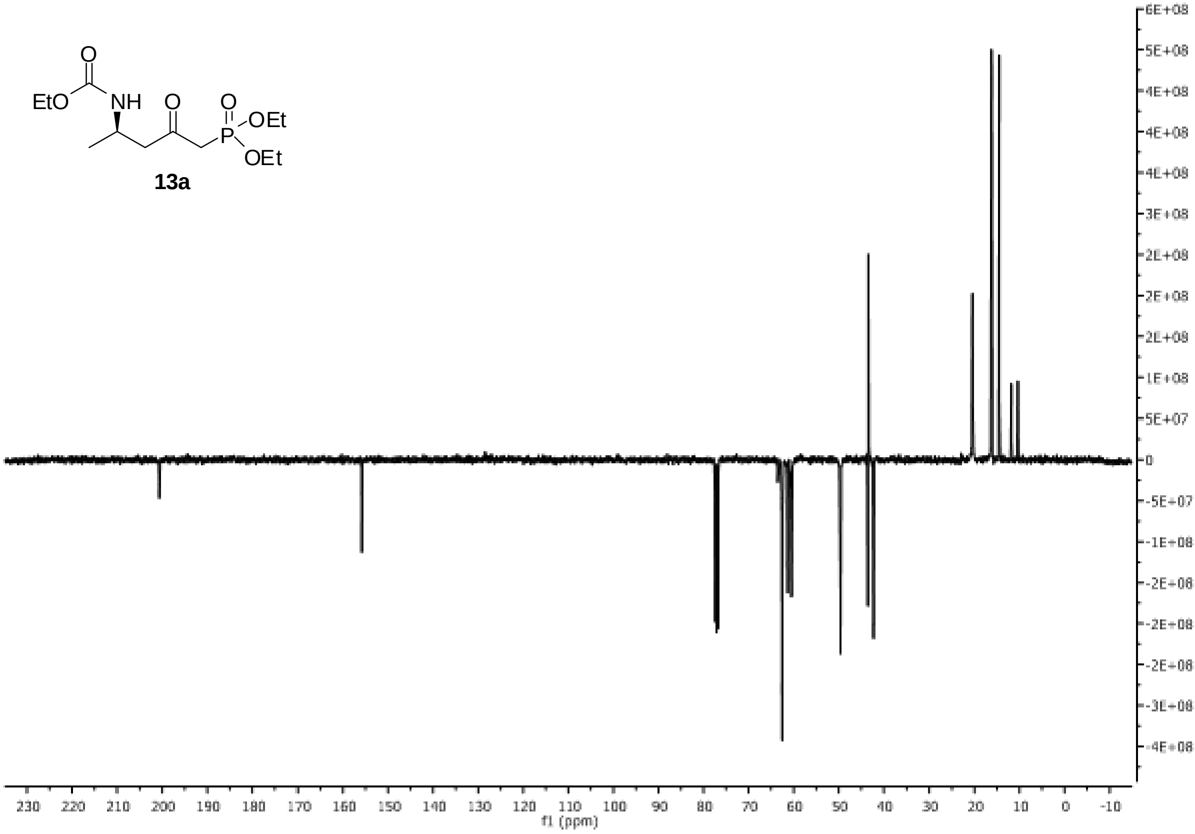

EtO
NH
O
O
P
OEt
OEt
13a
f1 (ppm)

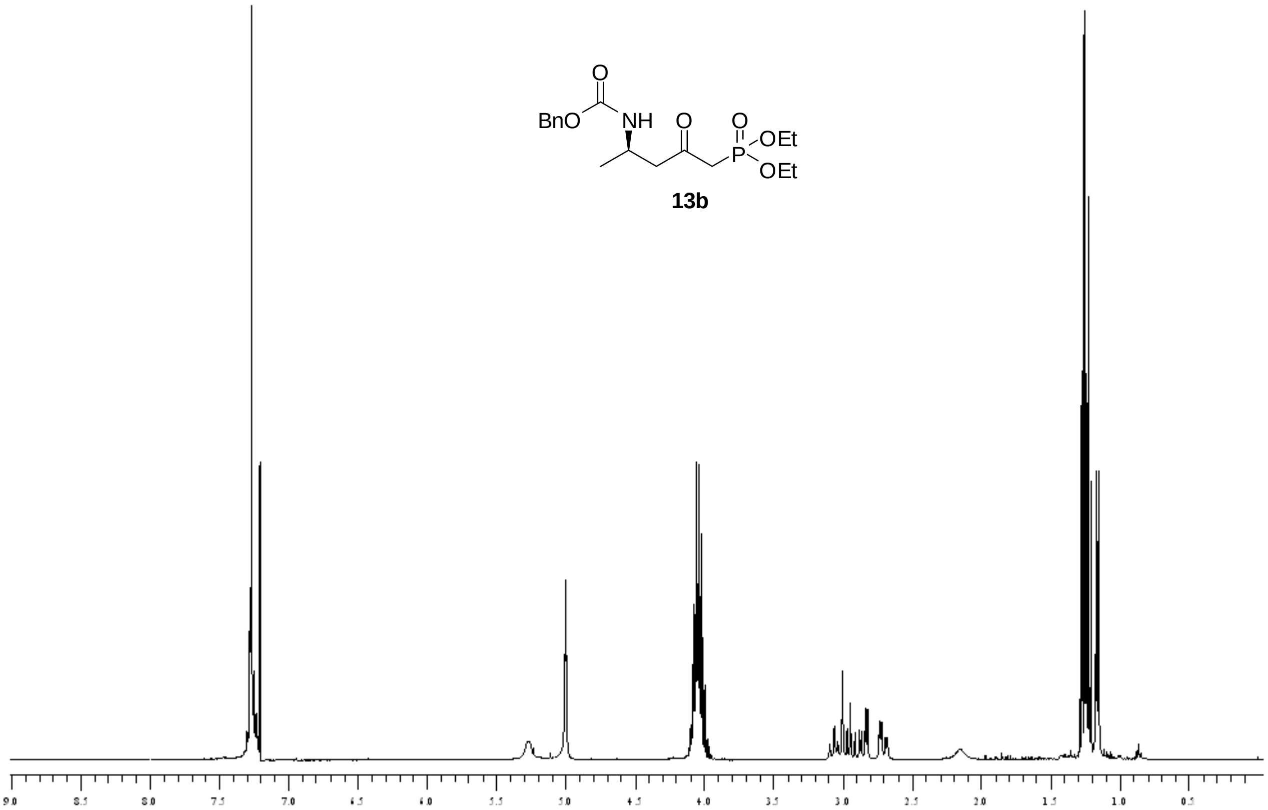
O
BnO NH O O
P OEt
OEt
13b
9.0 8.5 8.0 7.5 7.0 6.5 6.0 5.5 5.0 4.5 4.0 3.5 3.0 2.5 2.0 1.5 1.0 0.5

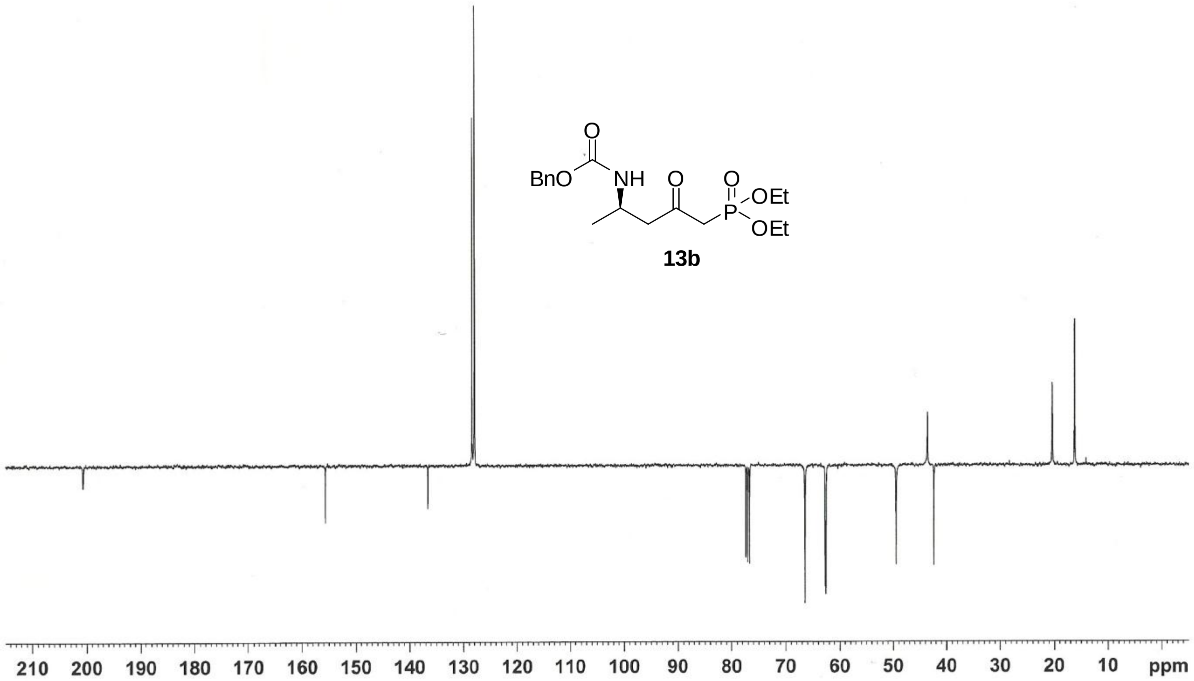
O
BnO NH O O
P OEt
OEt
13b
210 200 190 180 170 160 150 140 130 120 110 100 90 80 70 60 50 40 30 20 10 ppm

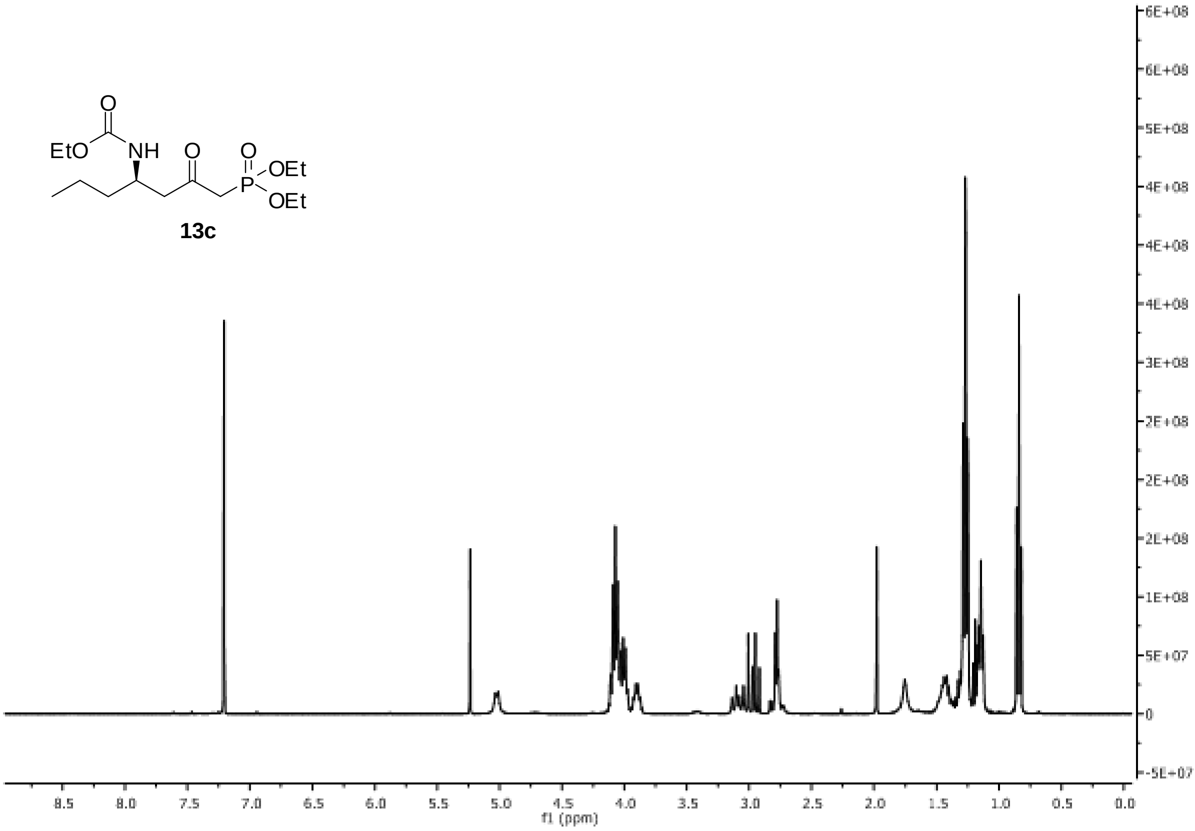
EtO
NH
OEt
OEt
13c
f1 (ppm)

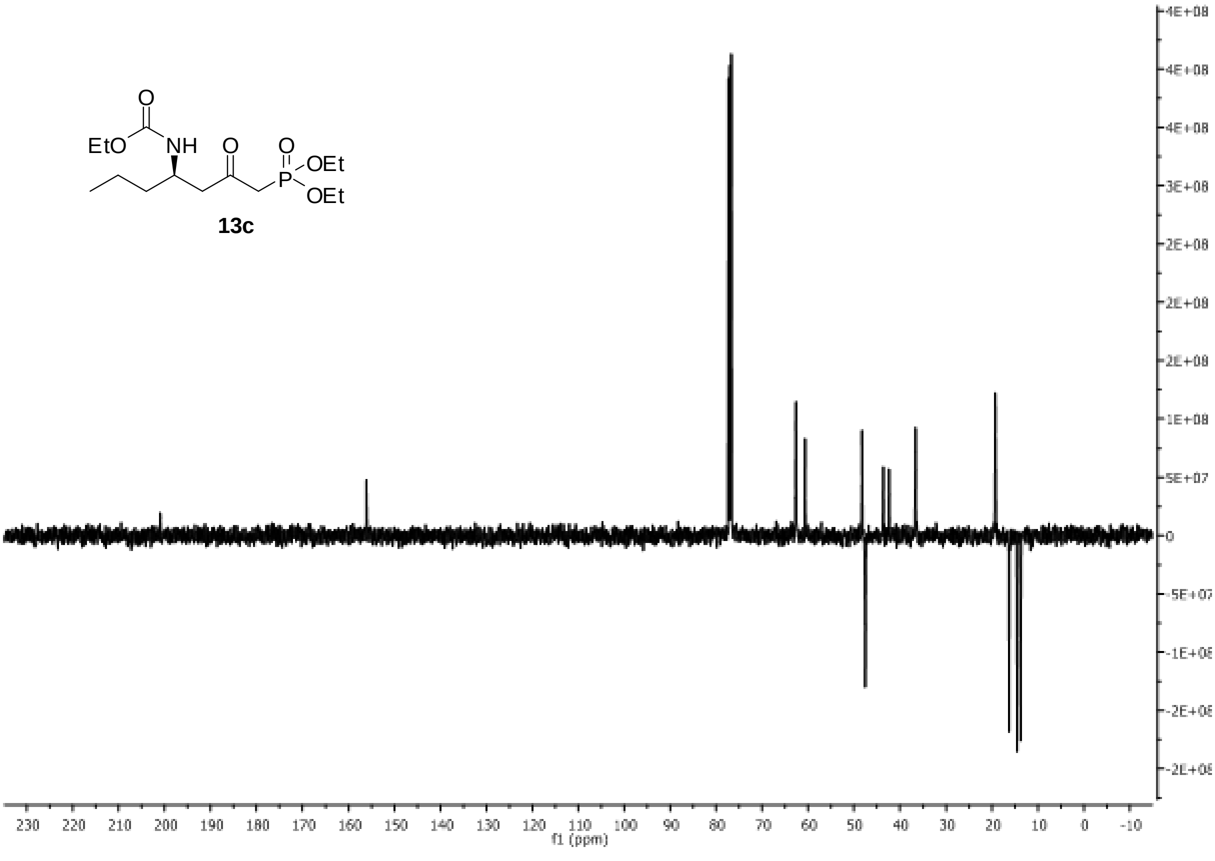
EtO
NH
OEt
OEt
13c
f1 (ppm)

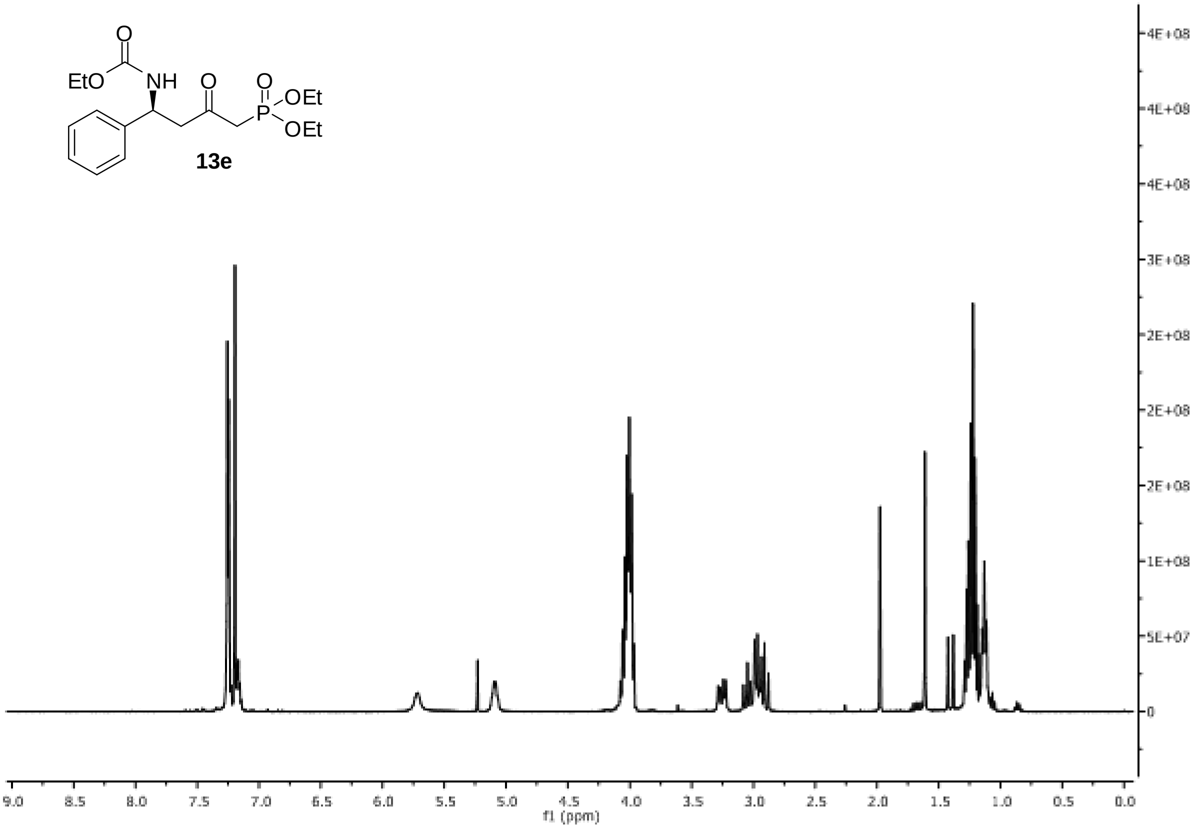
O
EtO
NH
O
O
P
OEt
OEt
13e
f1 (ppm)

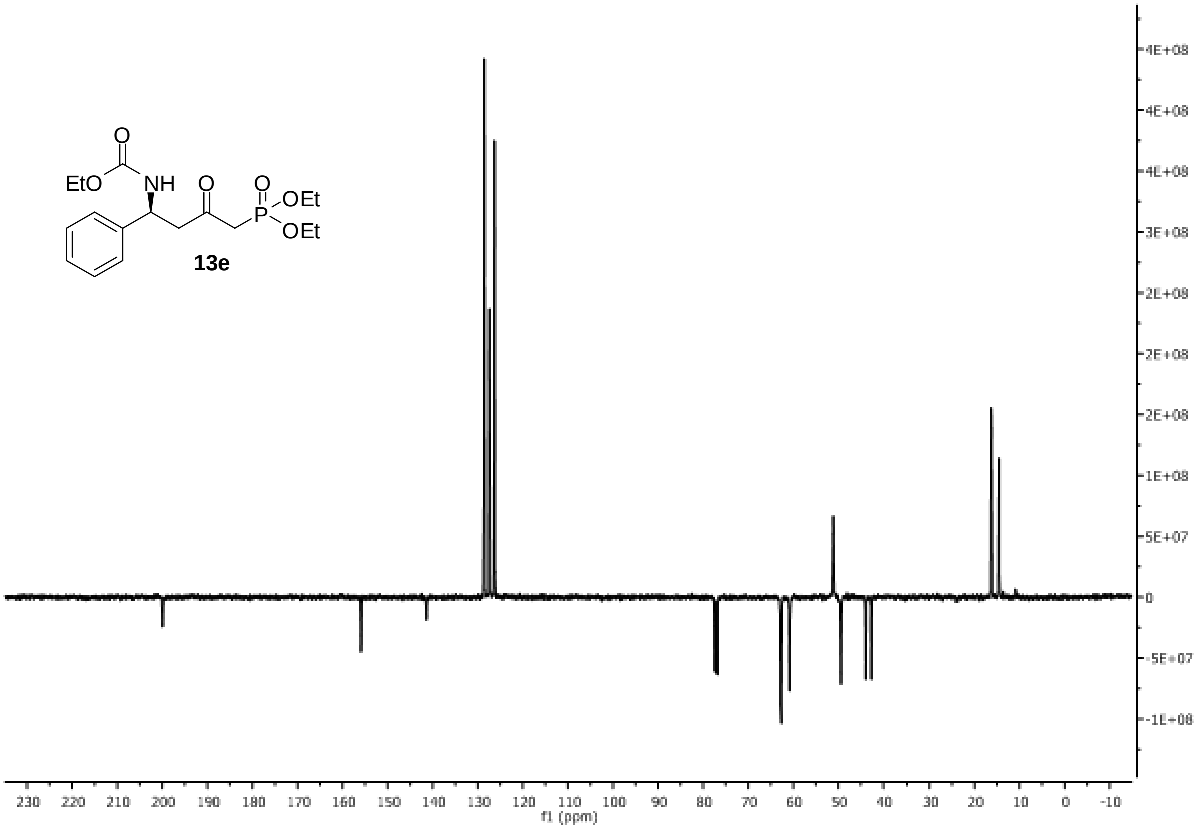
O
EtO
NH
O
O
P
OEt
OEt
13e
f1 (ppm)

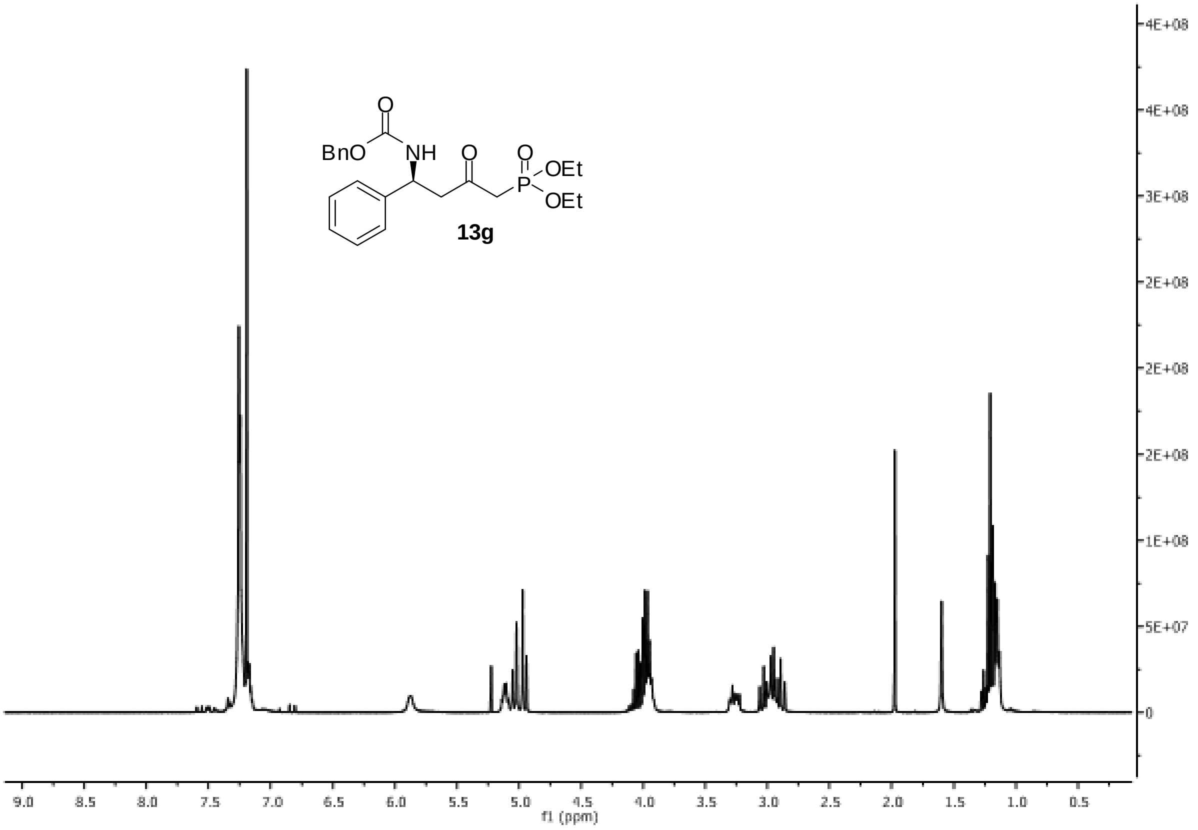
BnO
NH
O
O
OEt
P
OEt
13g
4E+08
3E+08
2E+08
1E+08
5E+07
0
9.0 8.5 8.0 7.5 7.0 6.5 6.0 5.5 5.0 4.5 4.0 3.5 3.0 2.5 2.0 1.5 1.0 0.5
f1 (ppm)

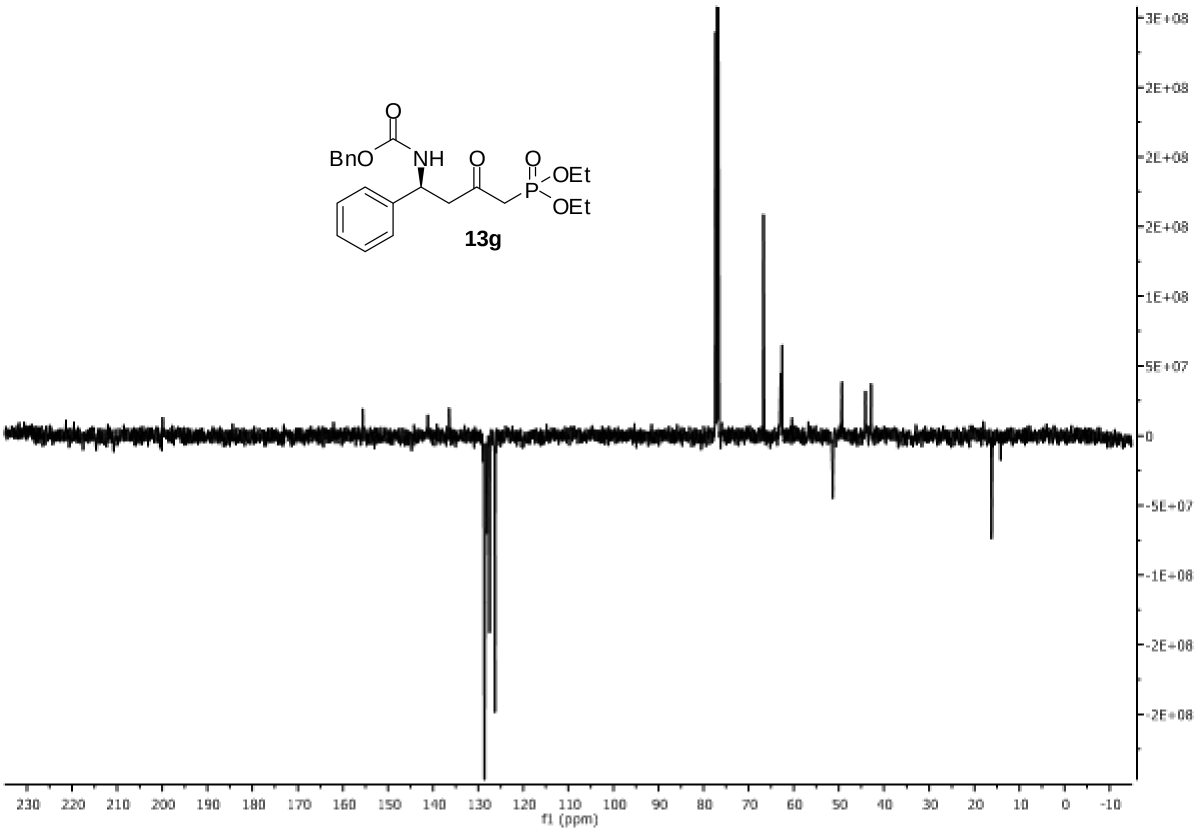
BnO
NH
O
O
OEt
P
OEt
13g
3E+08
2E+08
1E+08
5E+07
0
-5E+07
-1E+08
-2E+08
230 220 210 200 190 180 170 160 150 140 130 120 110 100 90 80 70 60 50 40 30 20 10 0 -10
f1 (ppm)

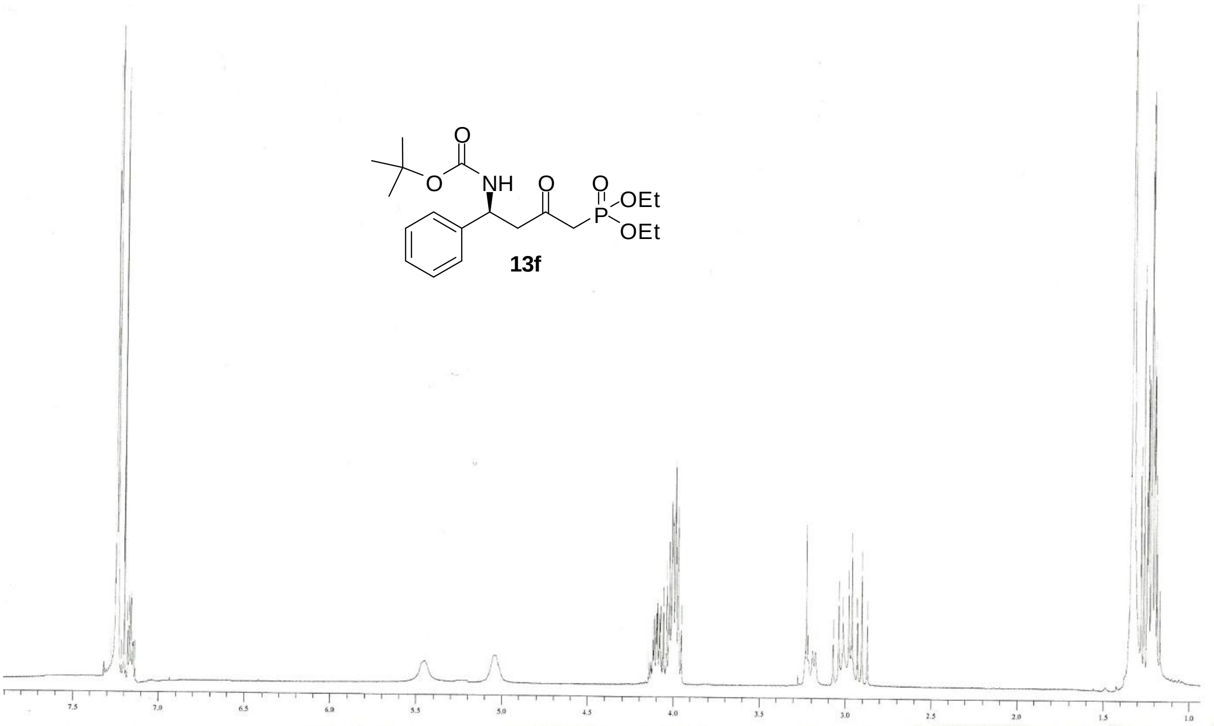
NH
O
OEt
OEt
13f
7.5 7.0 6.5 6.0 5.5 5.0 4.5 4.0 3.5 3.0 2.5 2.0 1.5 1.0

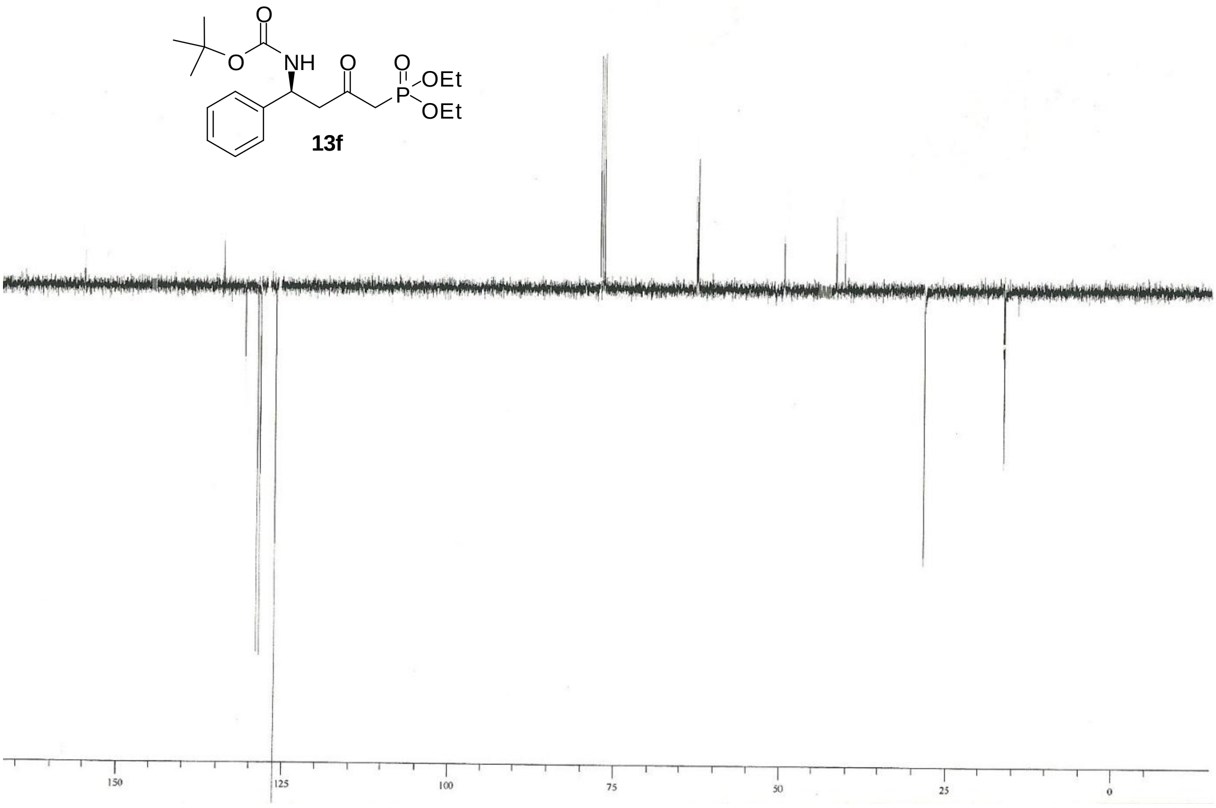
NH
O
OEt
OEt
13f
150 125 100 75 50 25 0

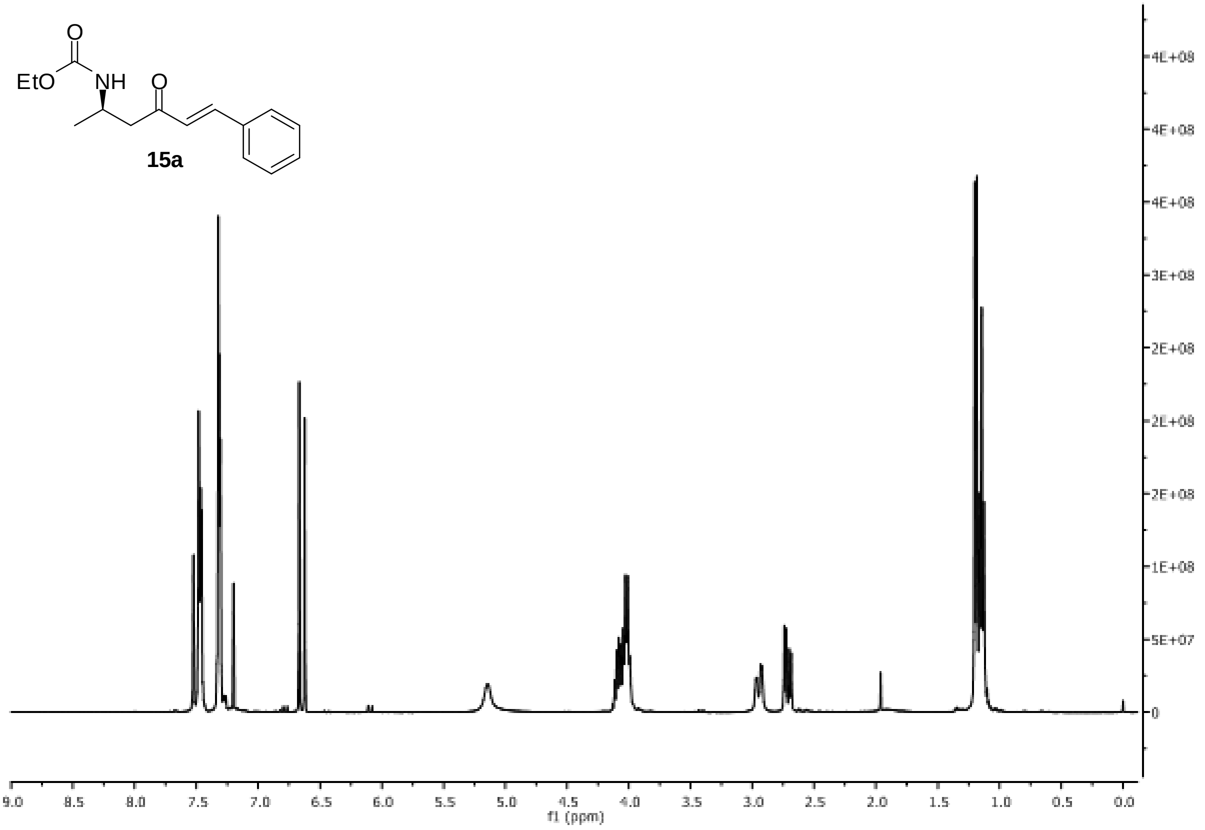
EtO
NH
O
O
15a
f1 (ppm)

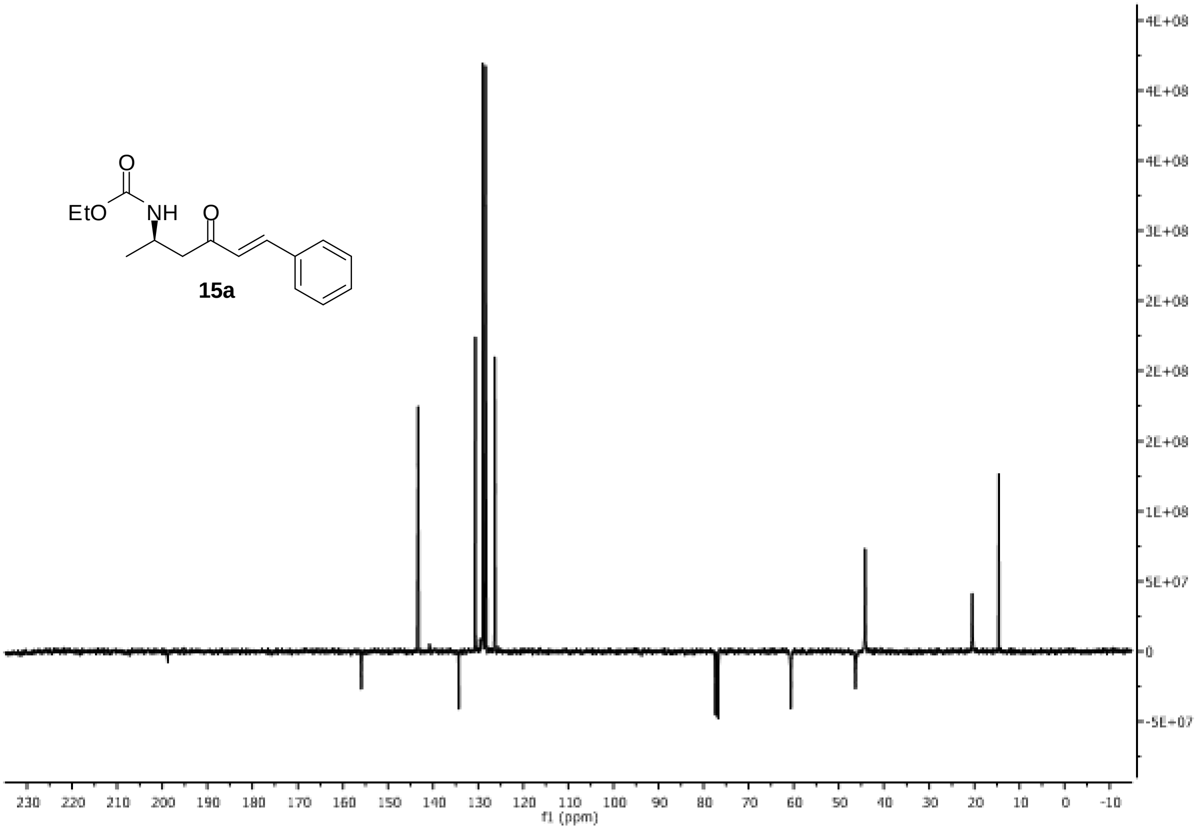
EtO
NH
O
O
15a
f1 (ppm)

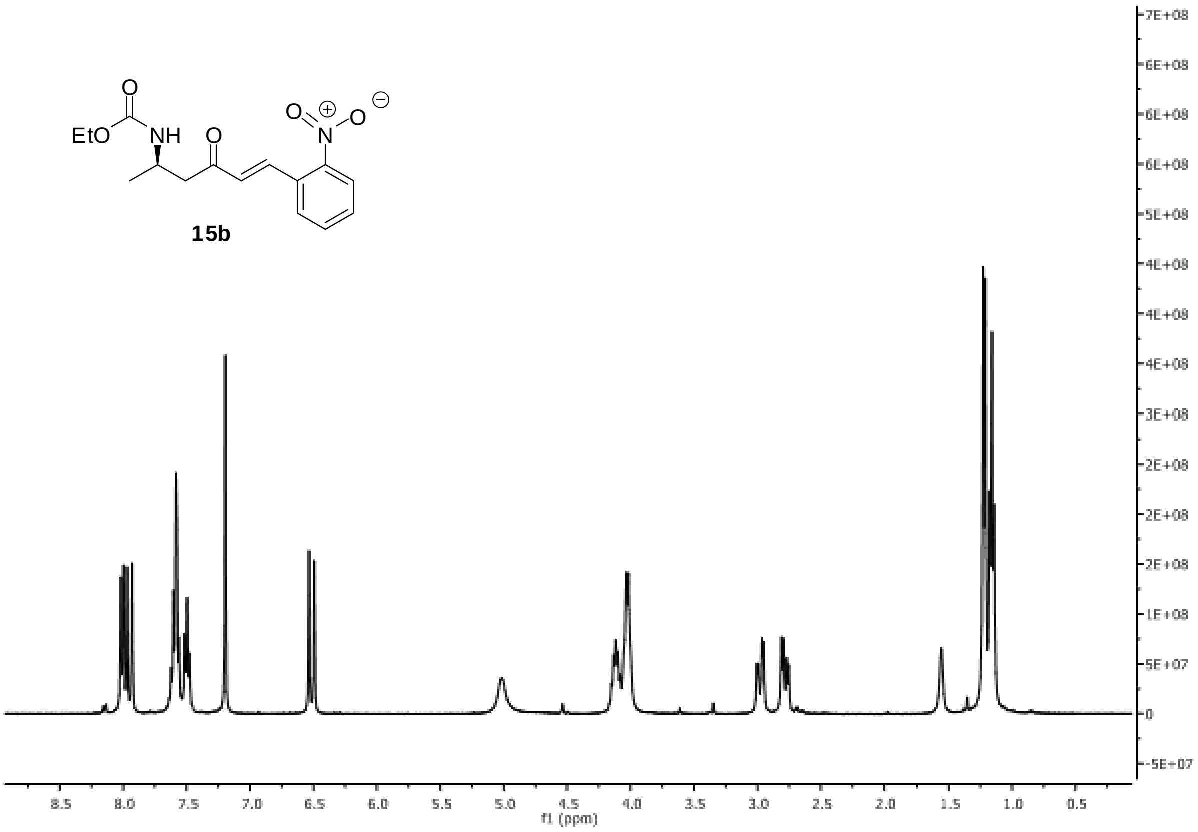
EtO
NH
15b
f1 (ppm)

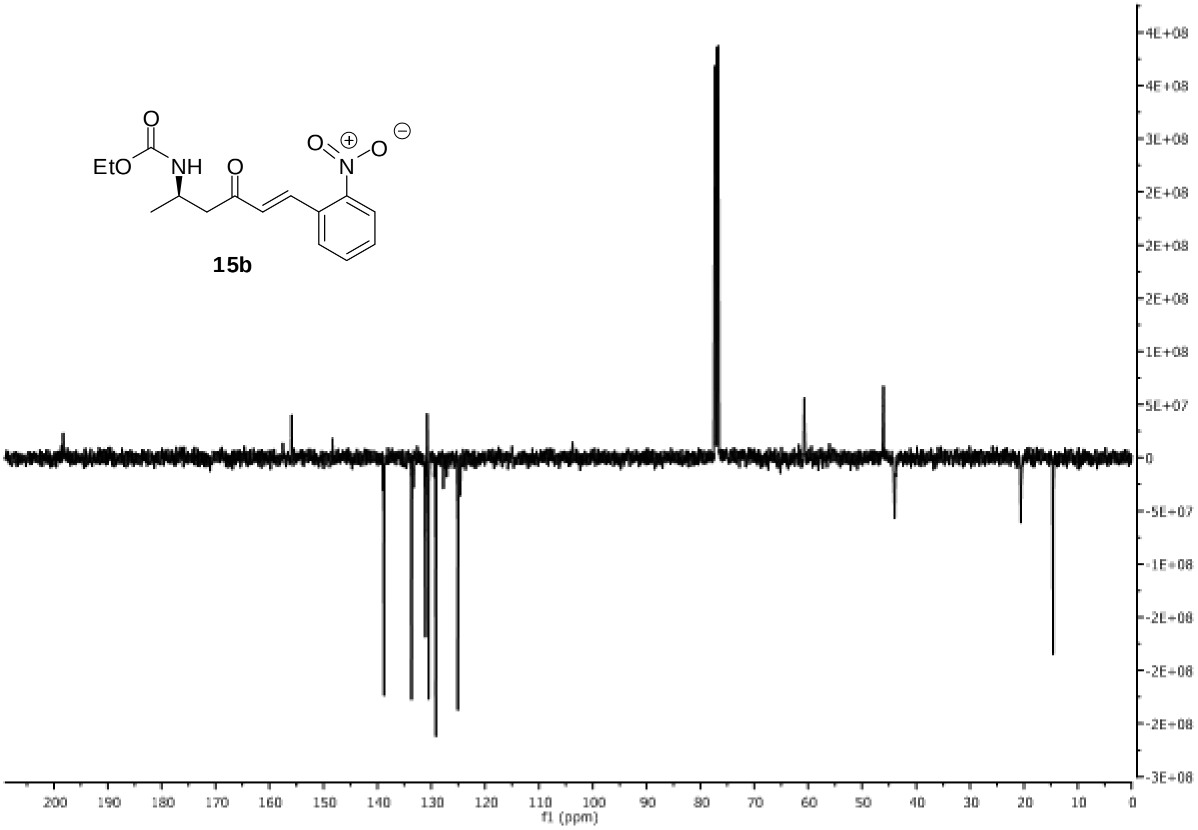
EtO
NH
15b
f1 (ppm)

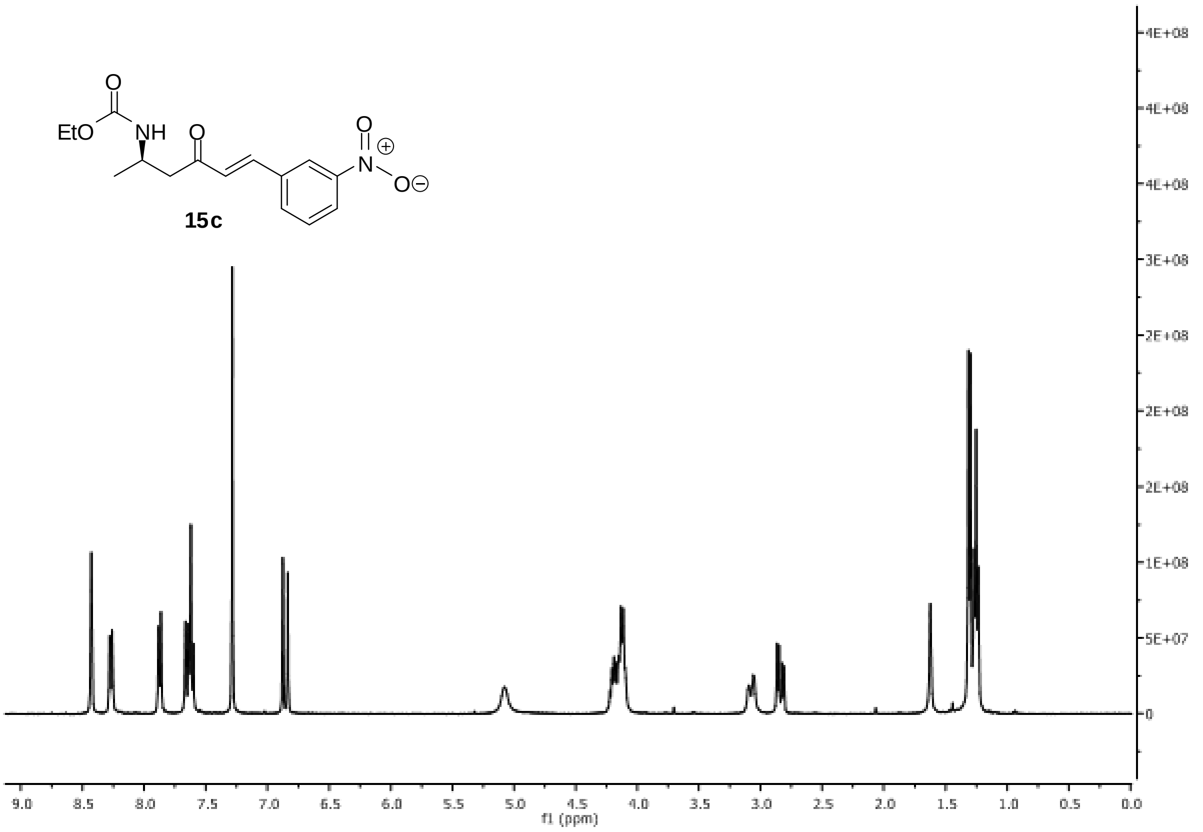

EtO
NH
O
N
15c
f1 (ppm)

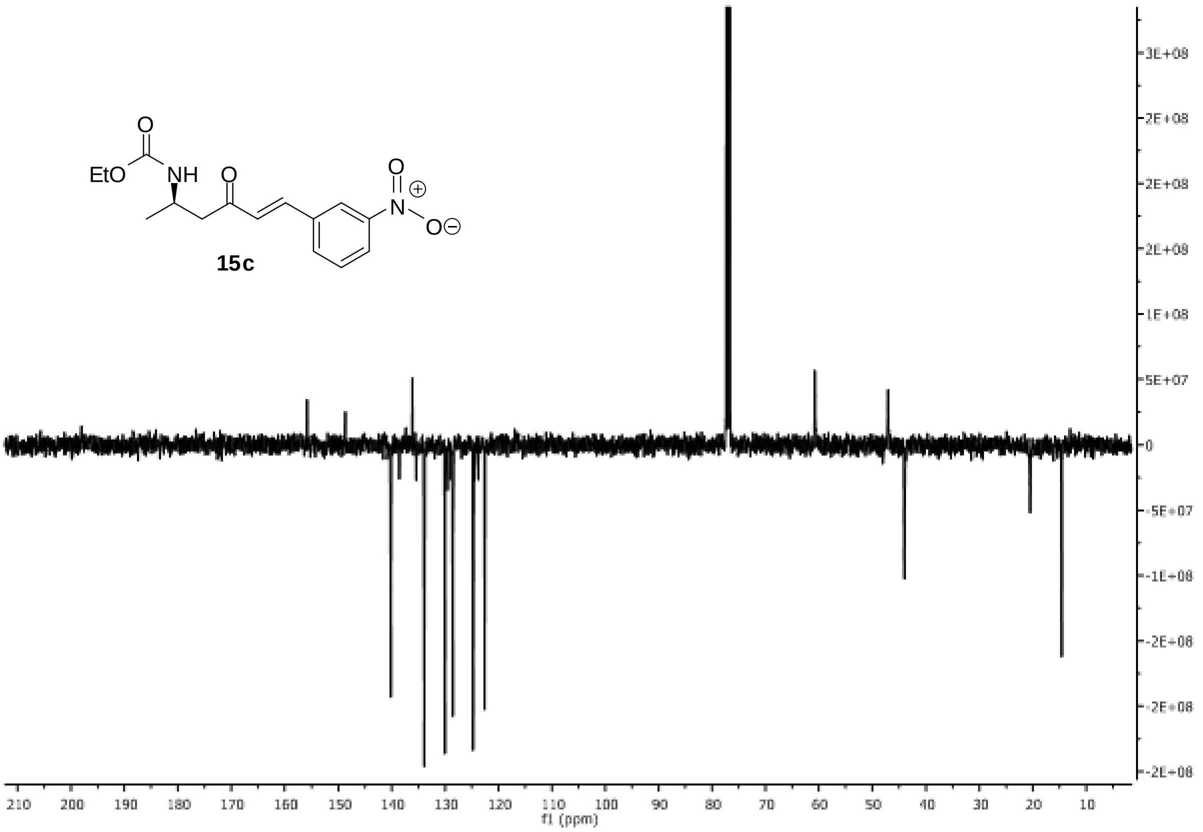

EtO
NH
O
N
15c
f1 (ppm)

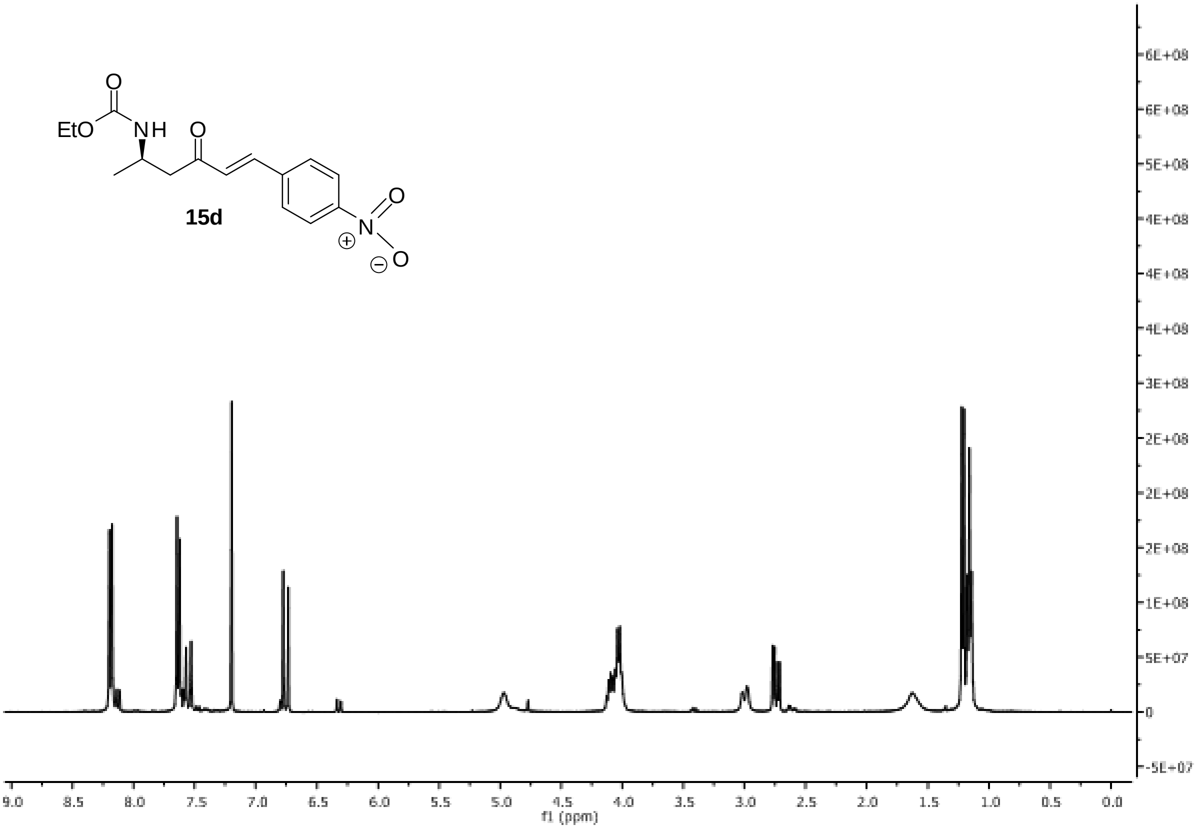
EtO
NH
O
O
N
O
O
15d
f1 (ppm)

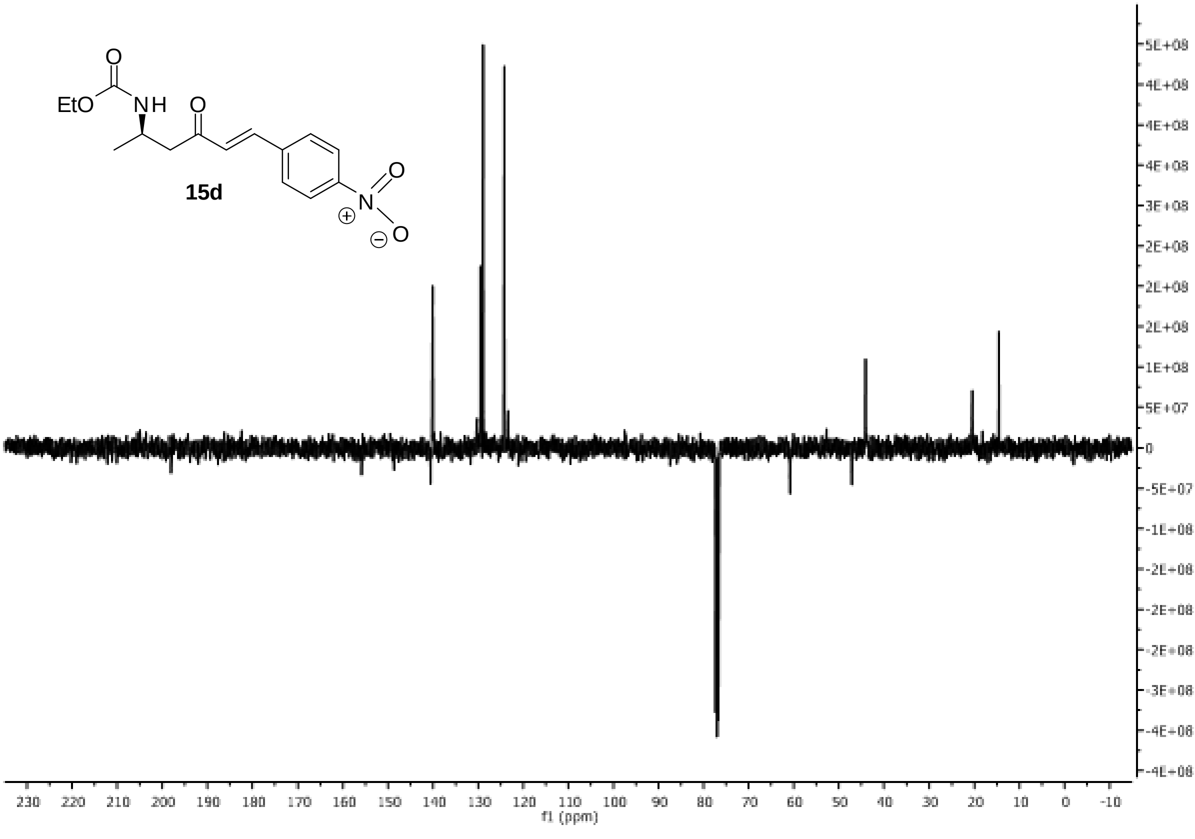
EtO
NH
O
O
N
O
O
15d
f1 (ppm)

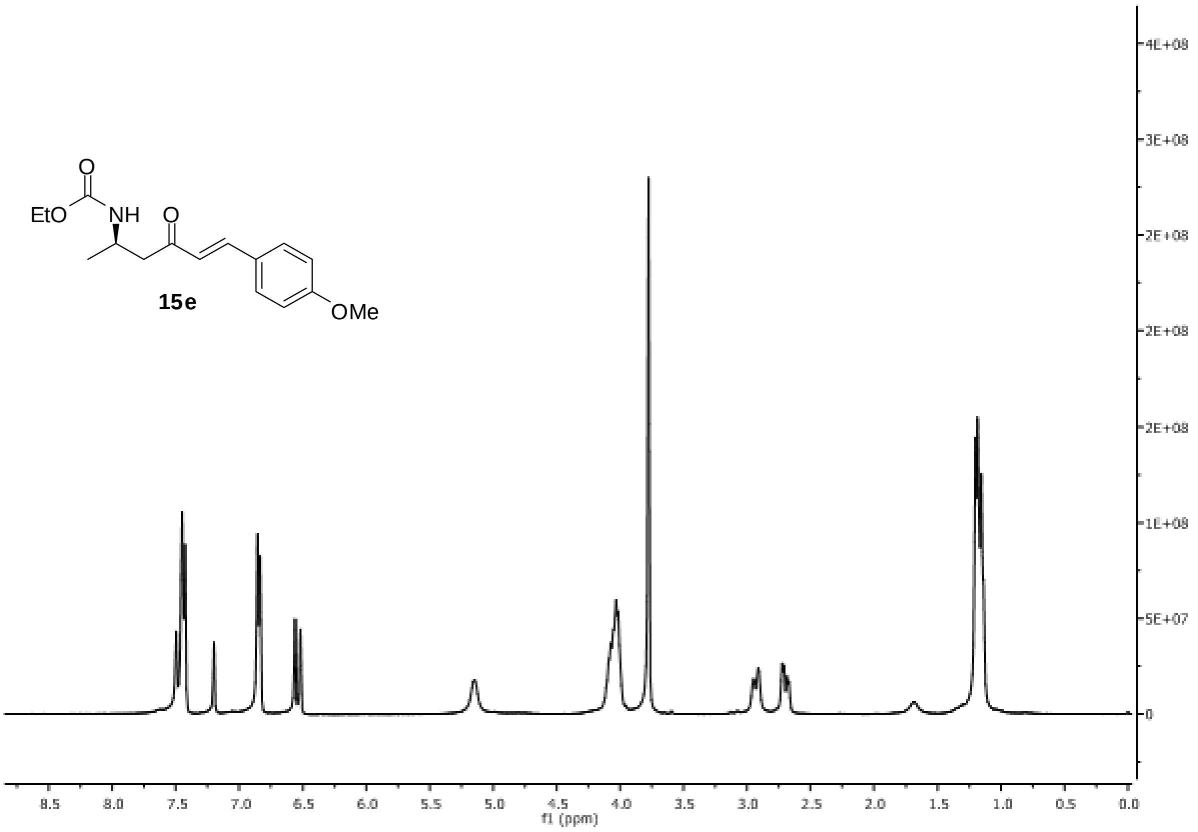
EtO
NH
O
O
15e
OMe
f1 (ppm)

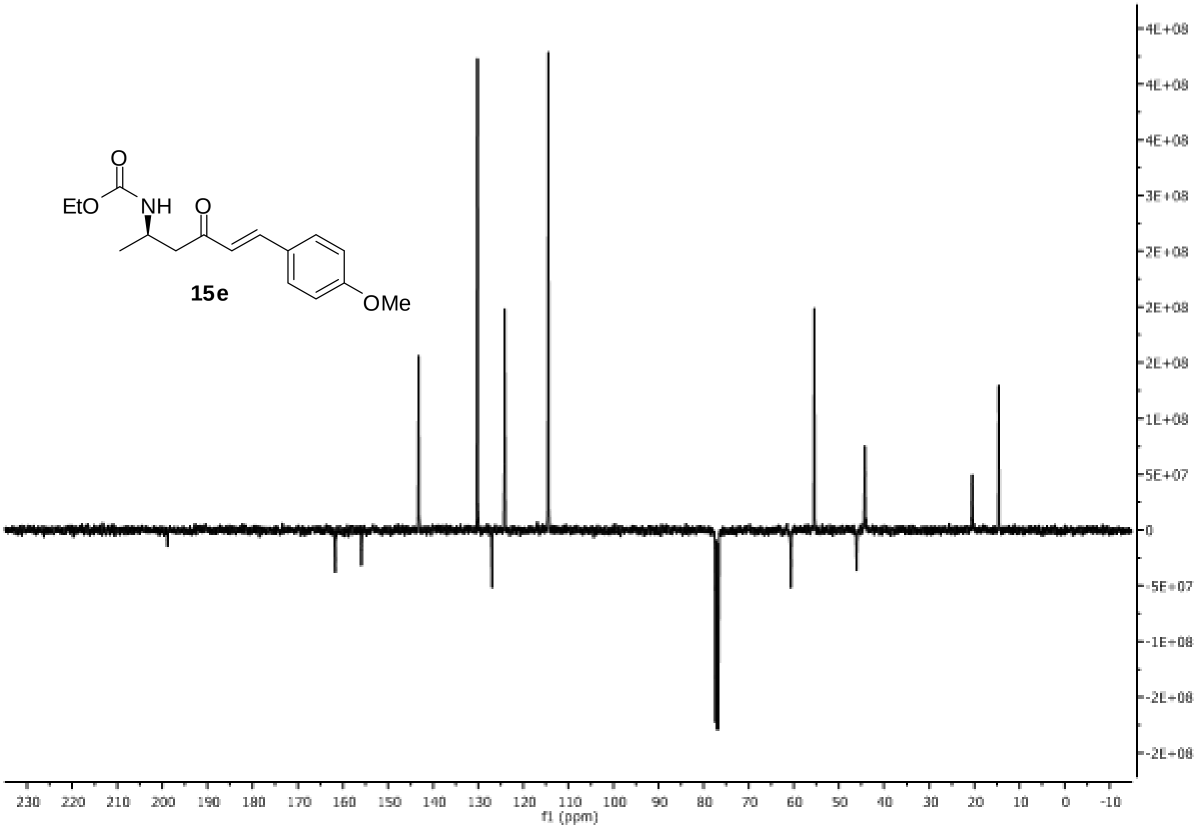
EtO
NH
O
O
15e
OMe
f1 (ppm)

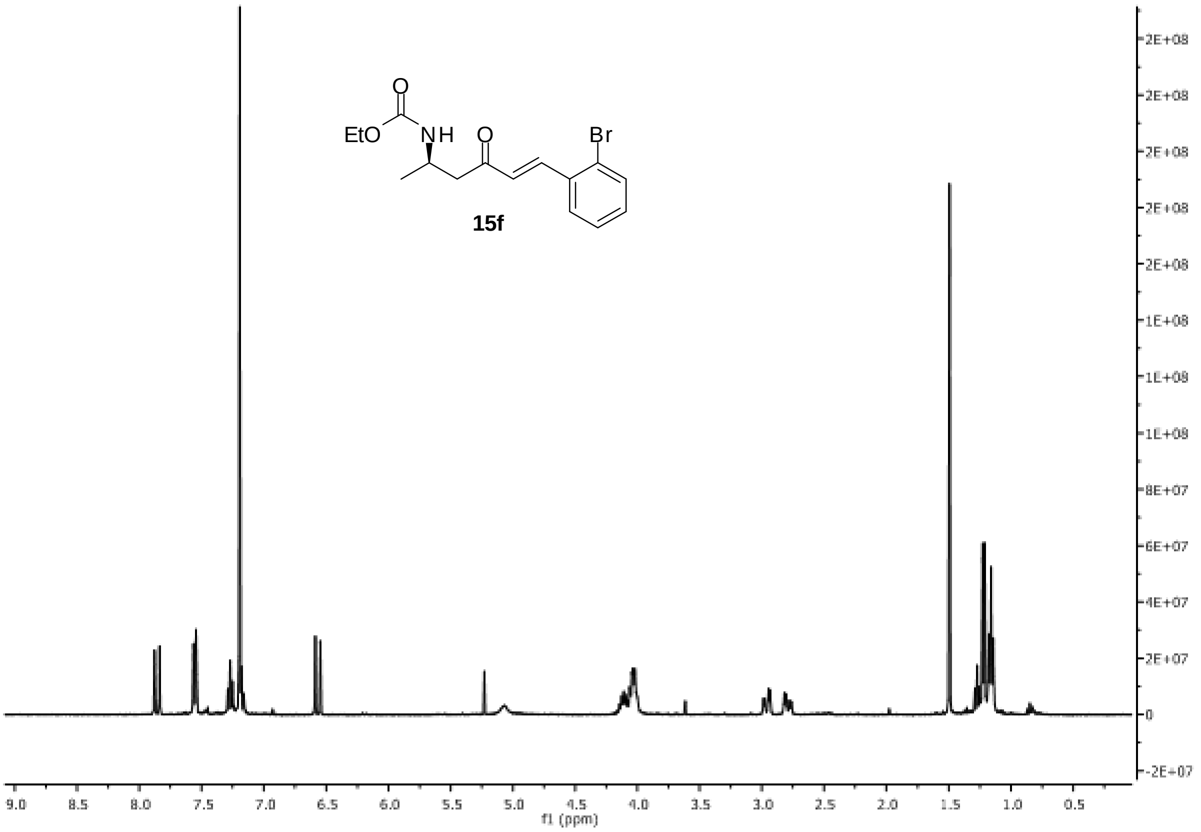
EtO
NH
O
Br
15f
f1 (ppm)

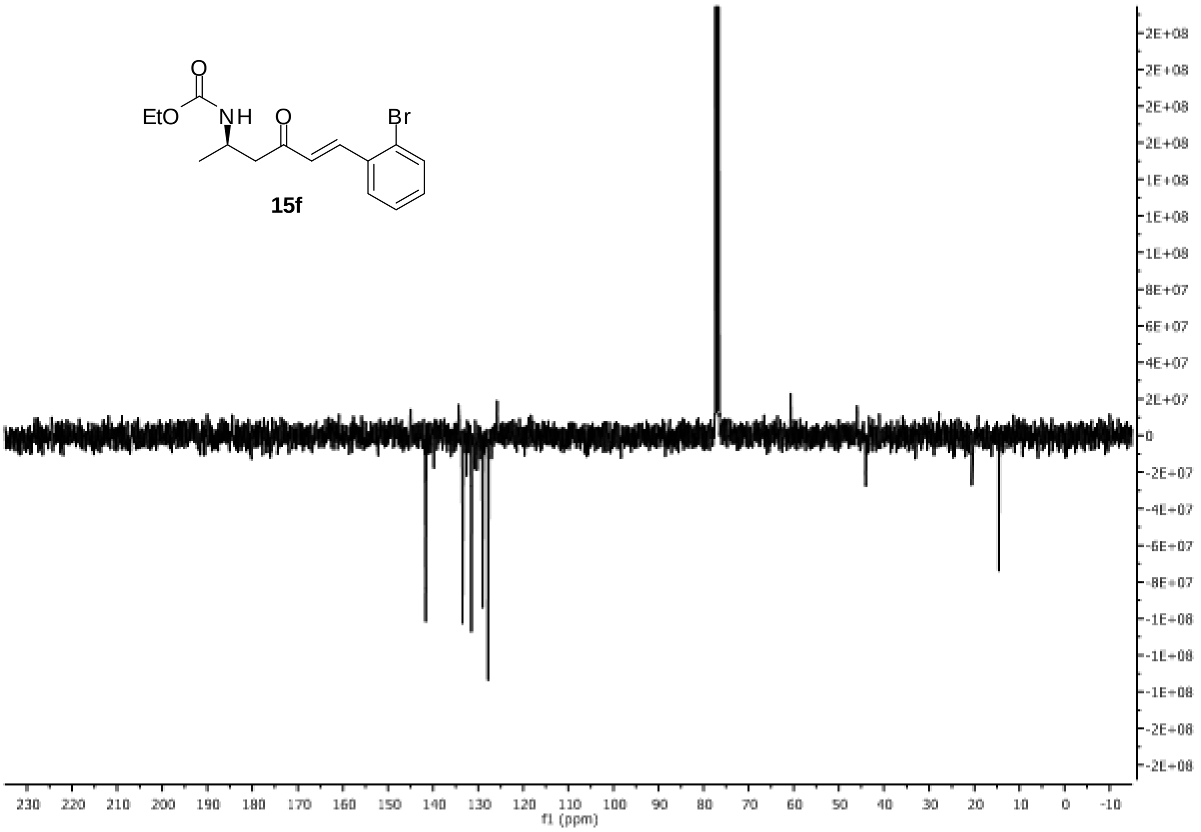
EtO
NH
O
Br
15f
f1 (ppm)

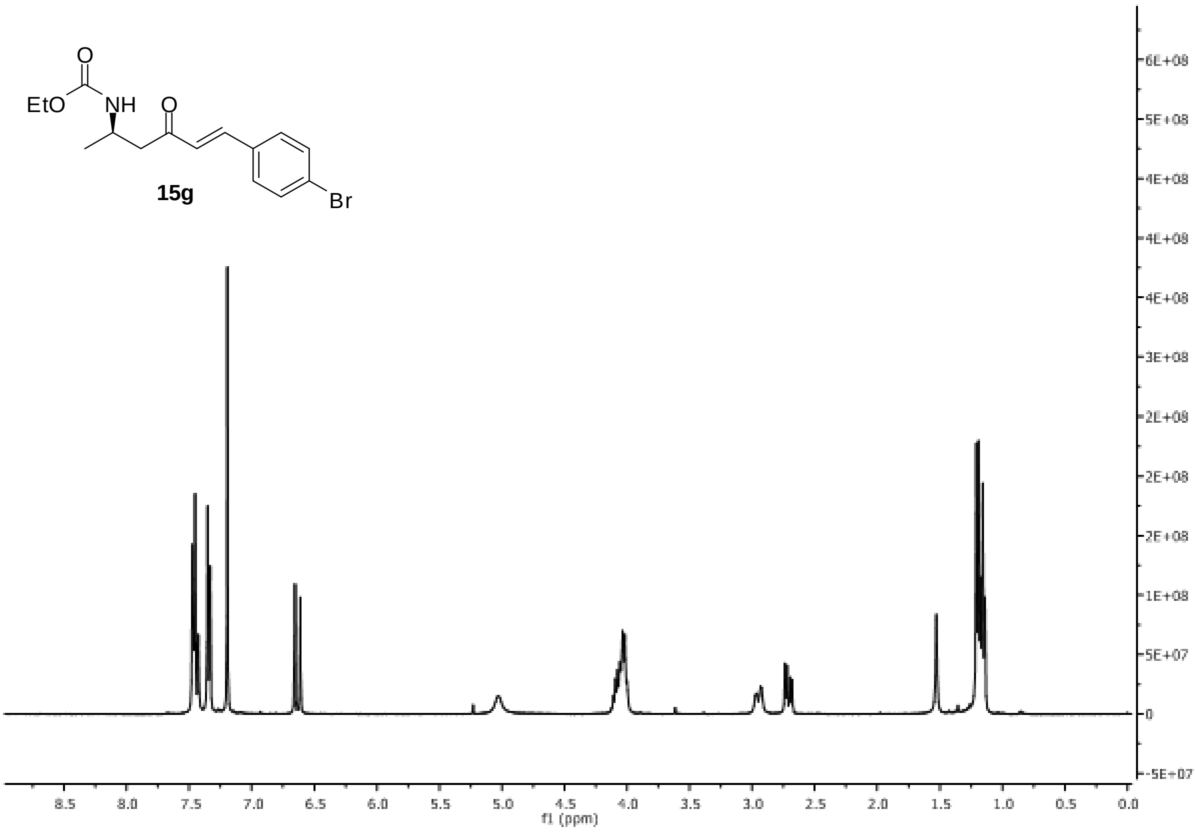
EtO
NH
O
O
Br
15g
f1 (ppm)

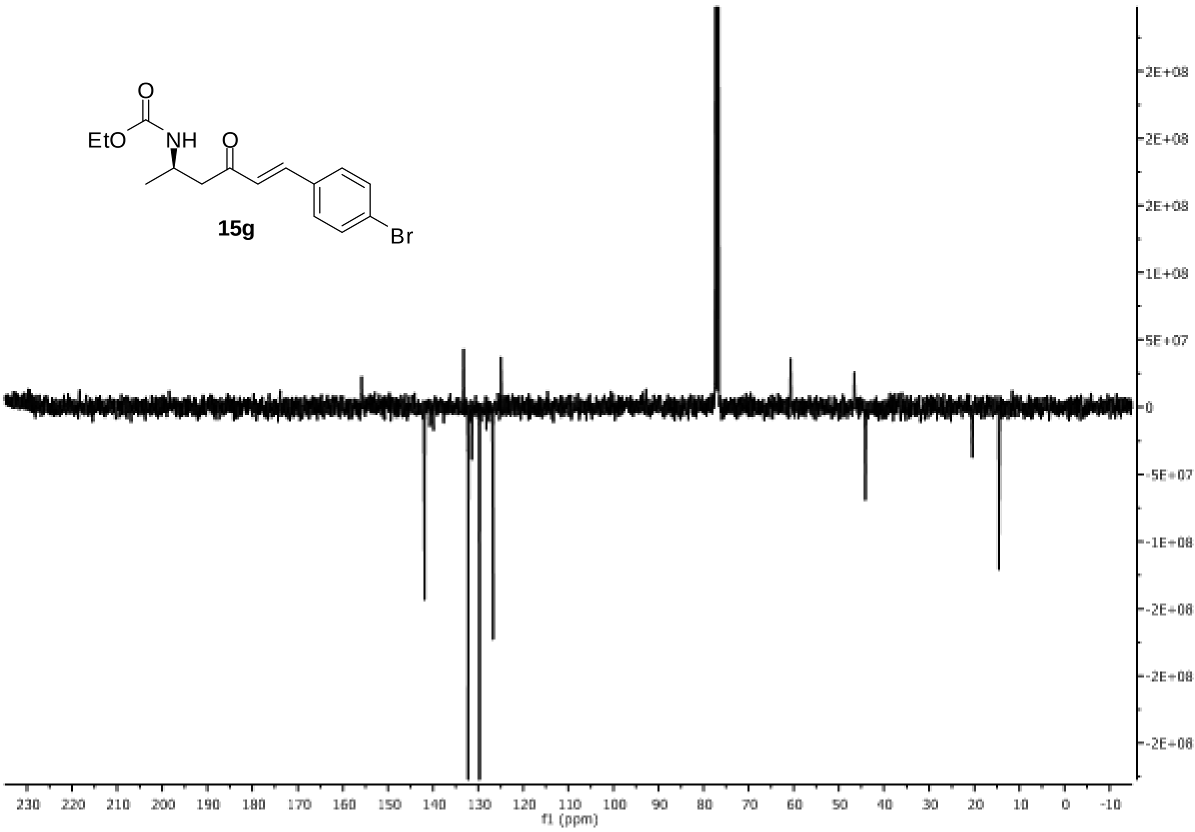
EtO
NH
O
O
Br
15g
f1 (ppm)

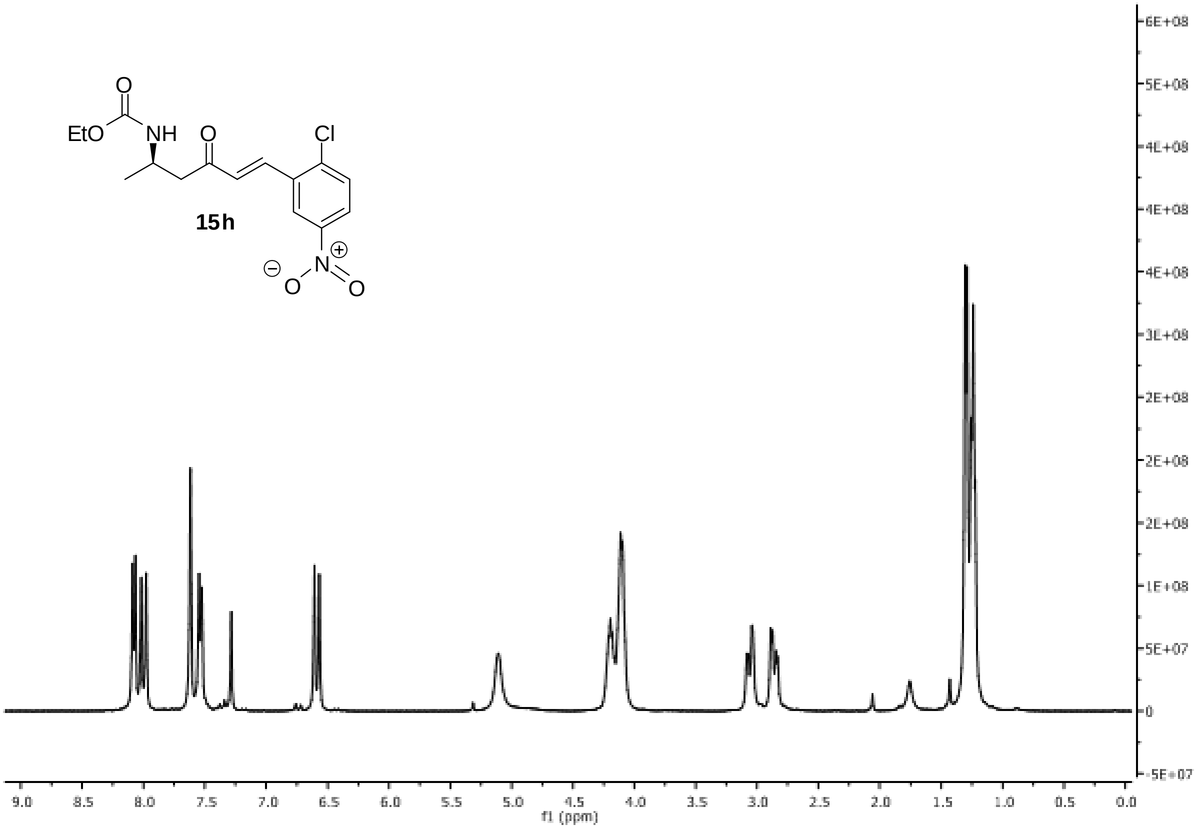
15h
f1 (ppm)

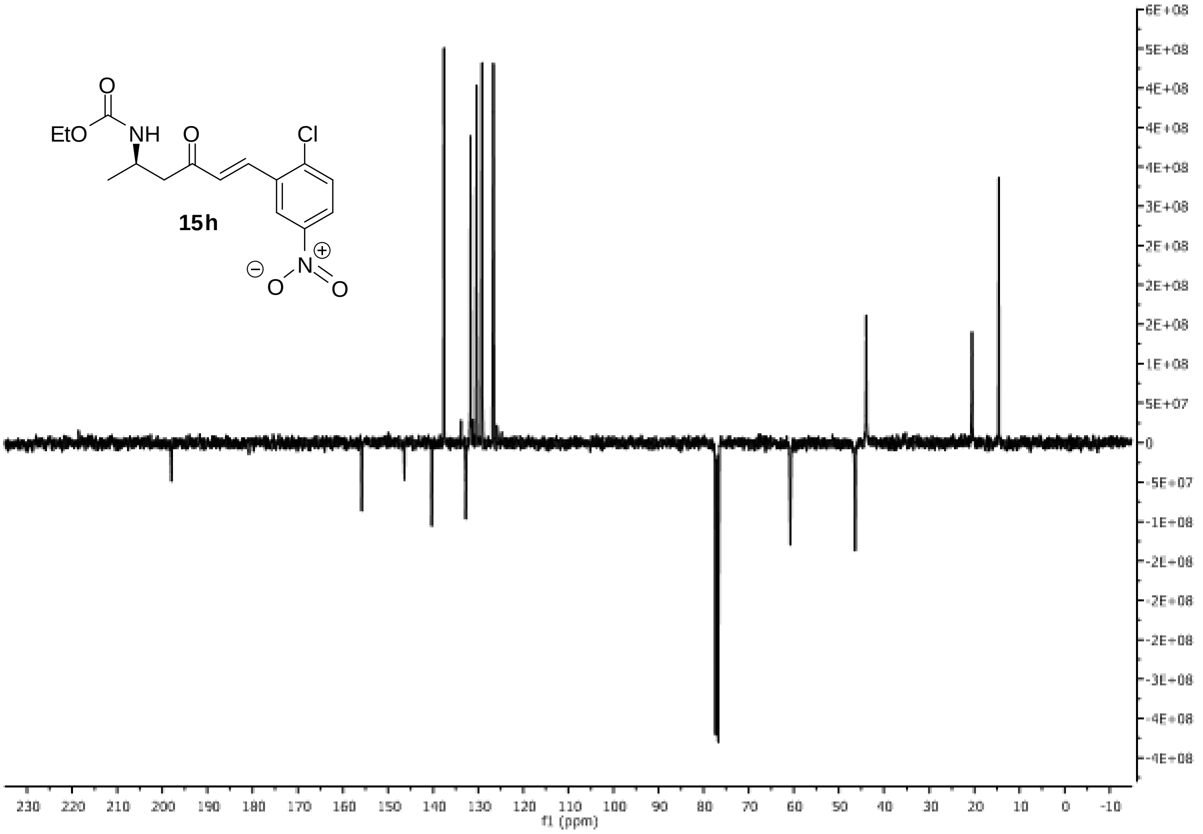
15h
f1 (ppm)

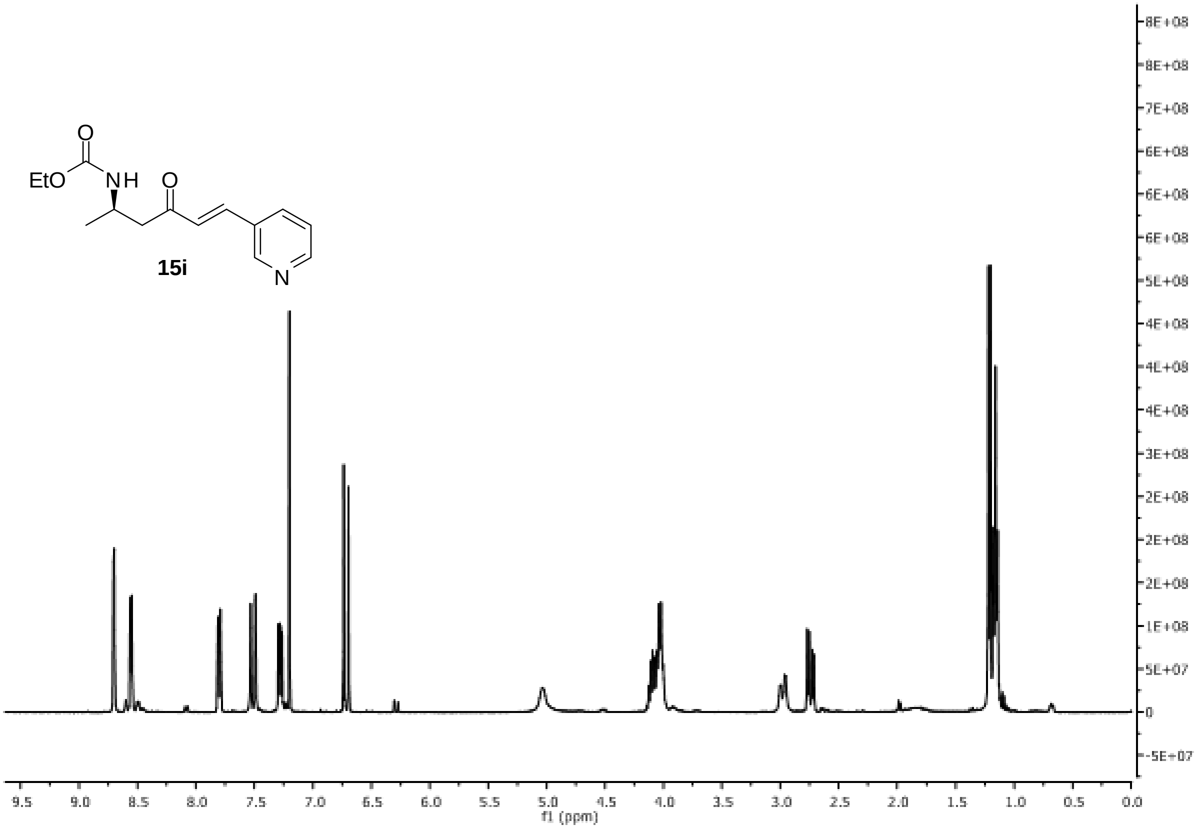
EtO
NH
O
O
N
15i
f1 (ppm)

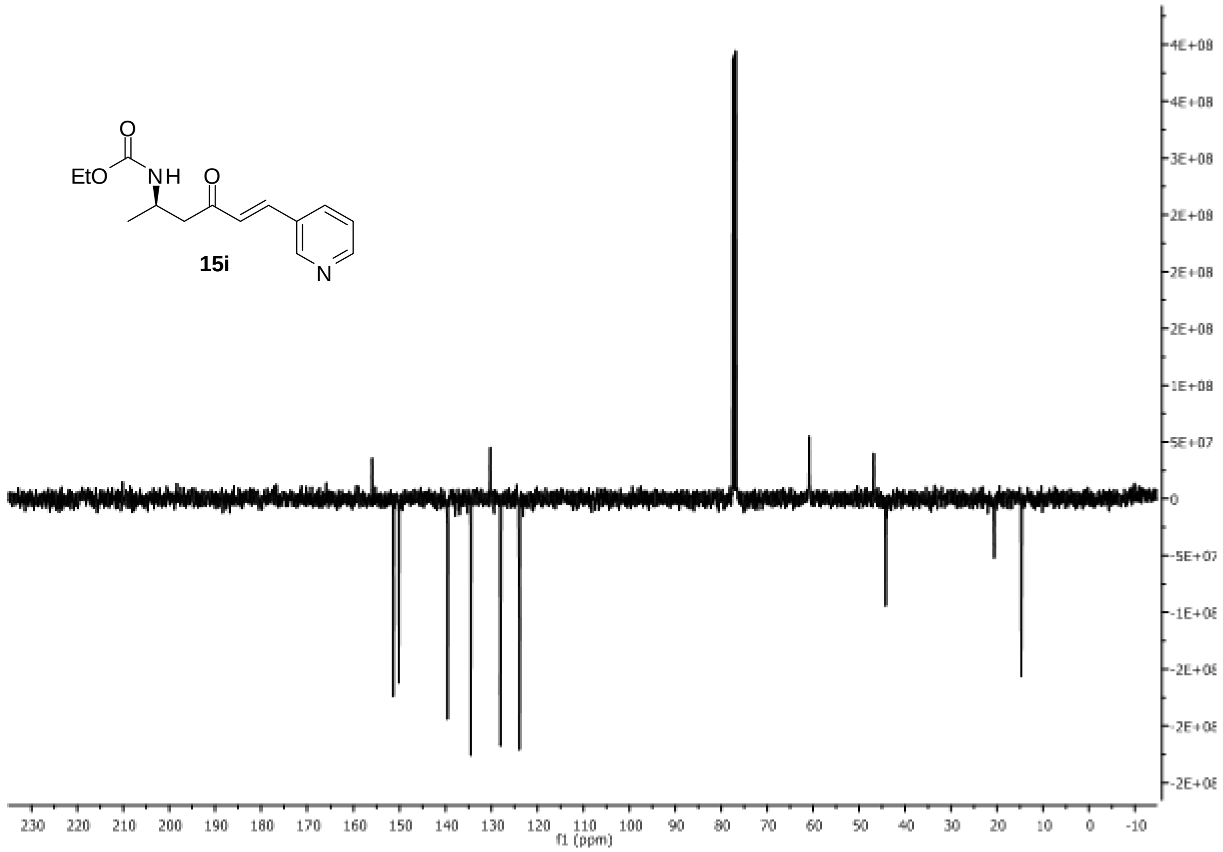
EtO
NH
O
O
N
15i
f1 (ppm)

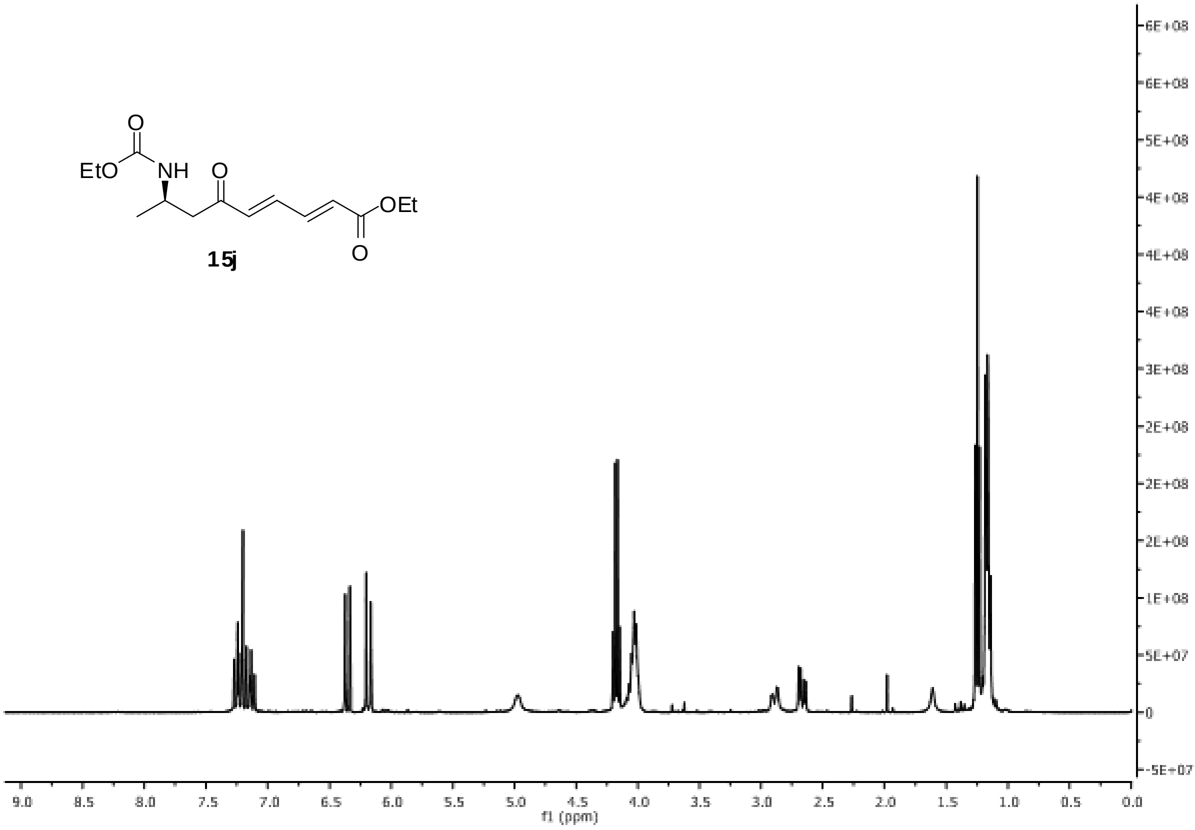
EtO
NH
O
O
OEt
O
15j
f1 (ppm)

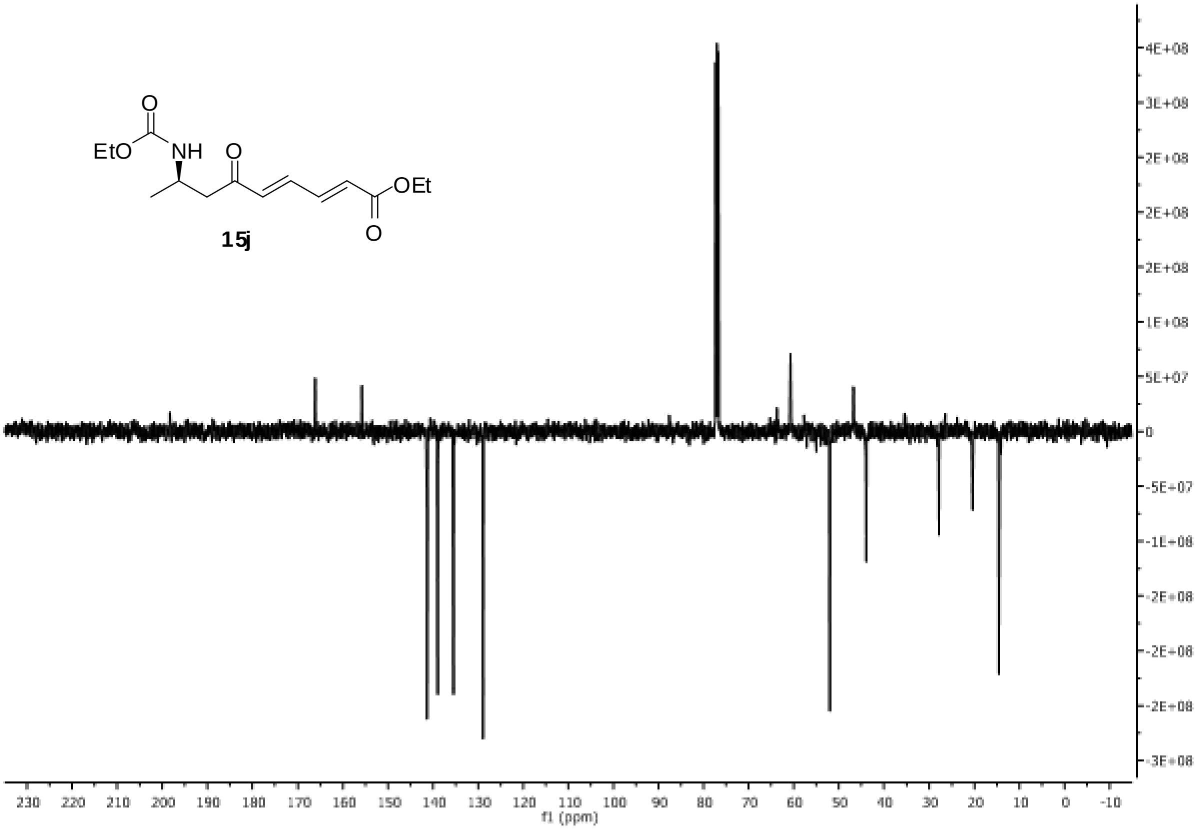
EtO
NH
O
O
OEt
O
15j
f1 (ppm)

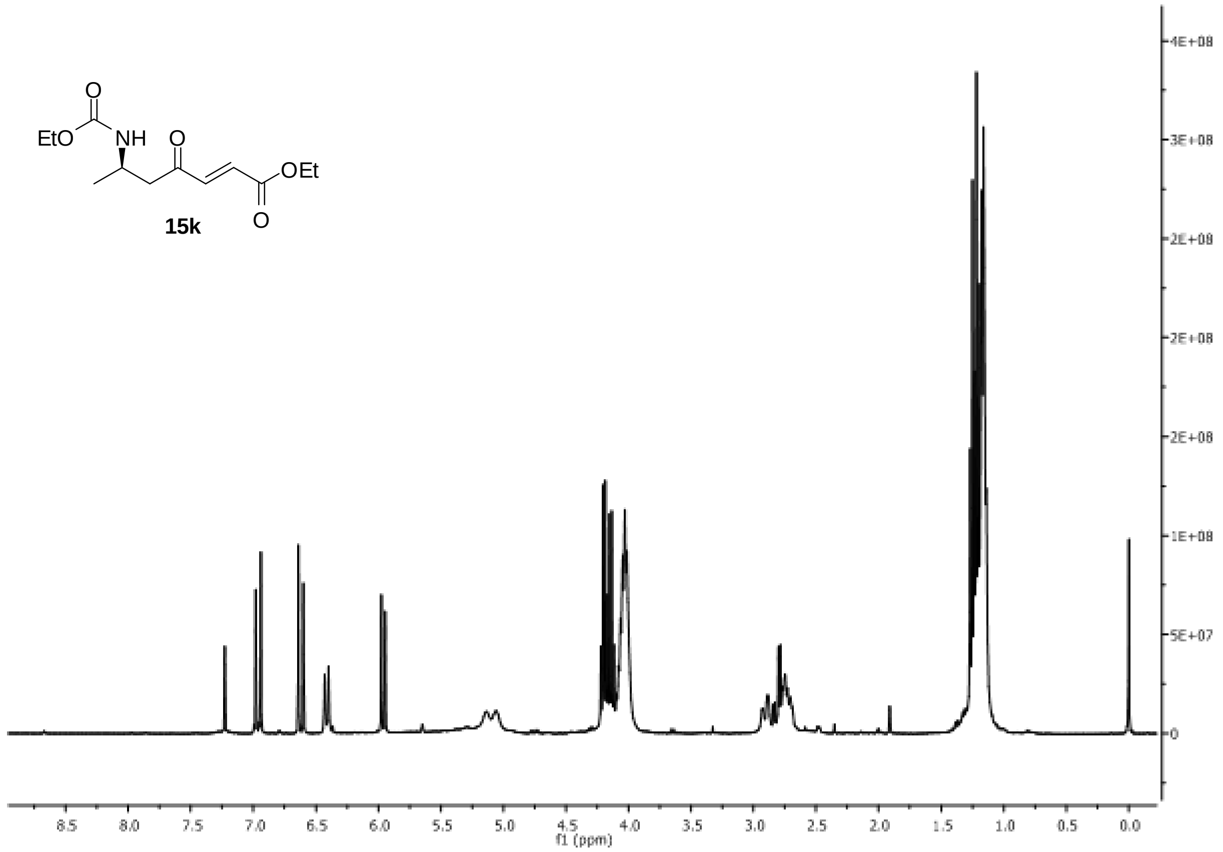
EtO
NH
O
O
OEt
O
15k
f1 (ppm)

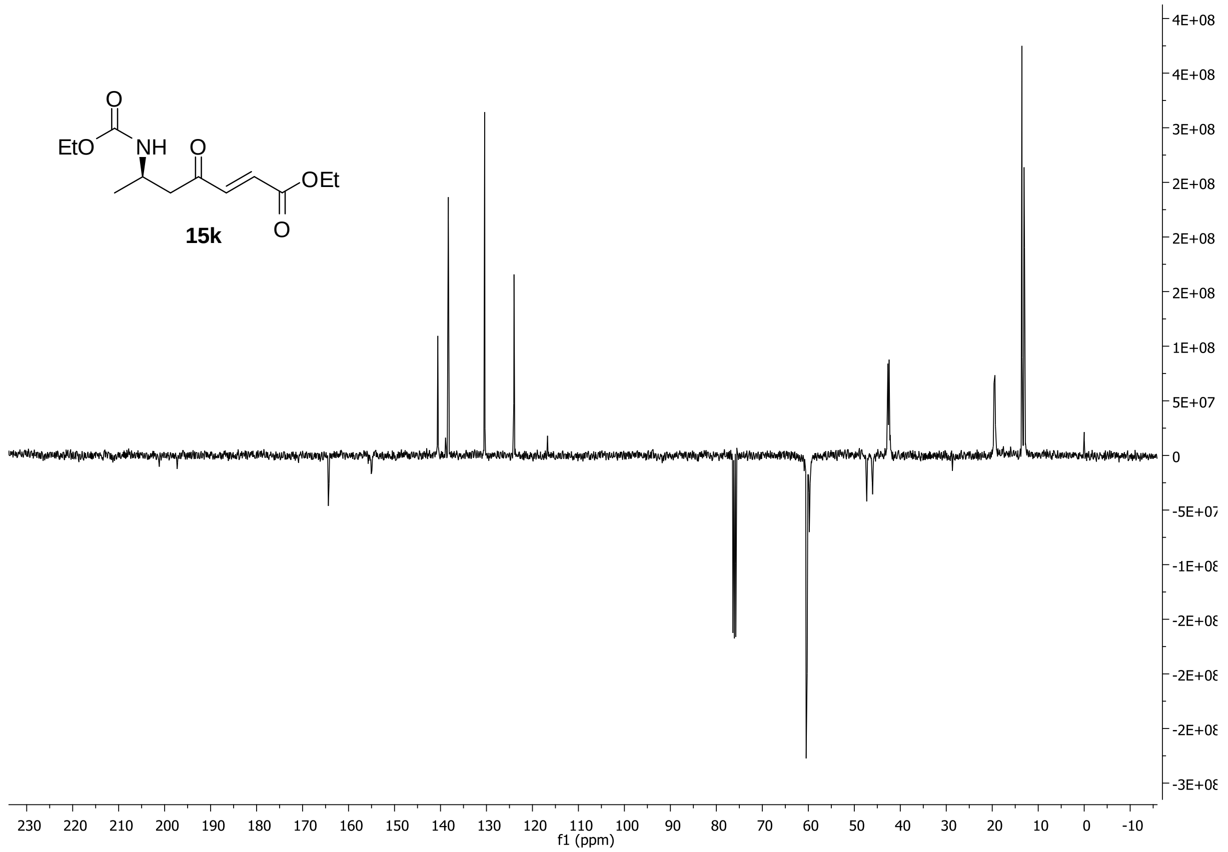
EtO
NH
O
O
OEt
O
15k
f1 (ppm)

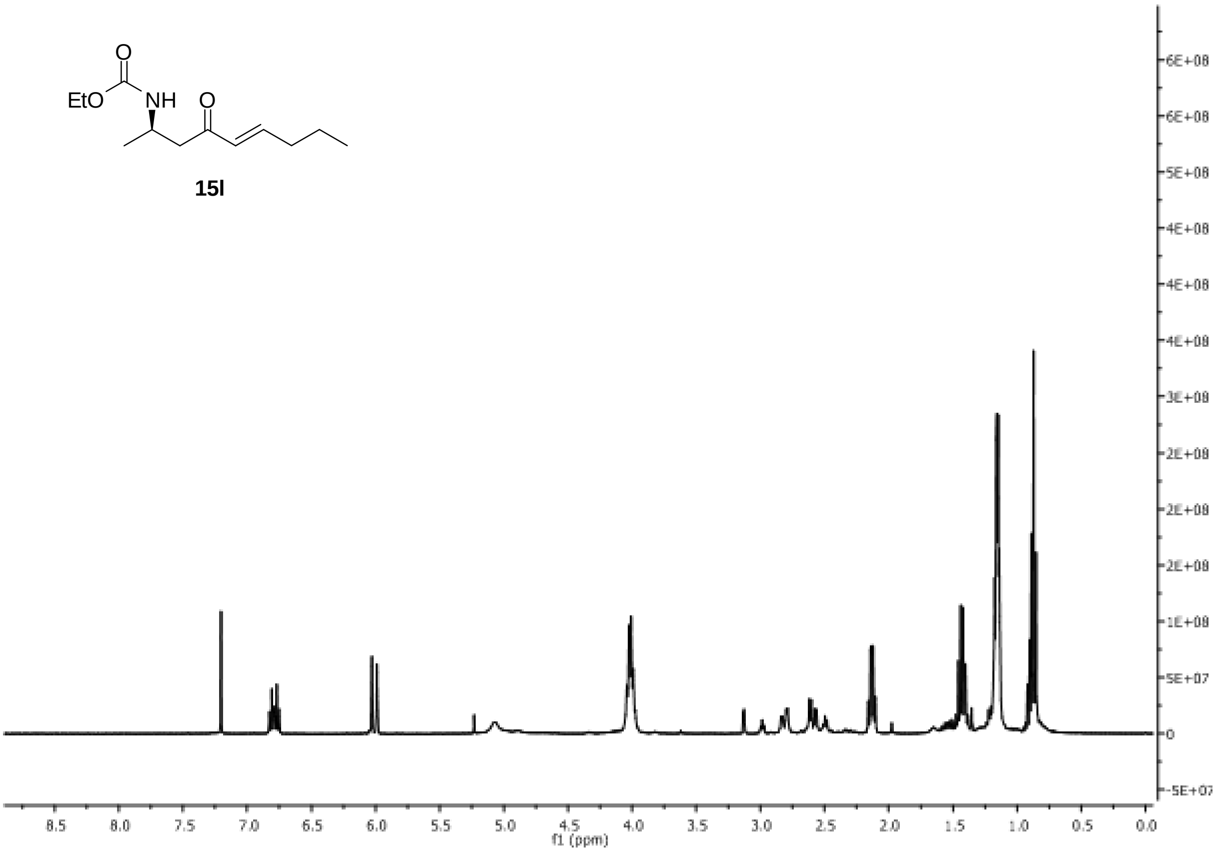
EtO
NH
O
15l
f1 (ppm)

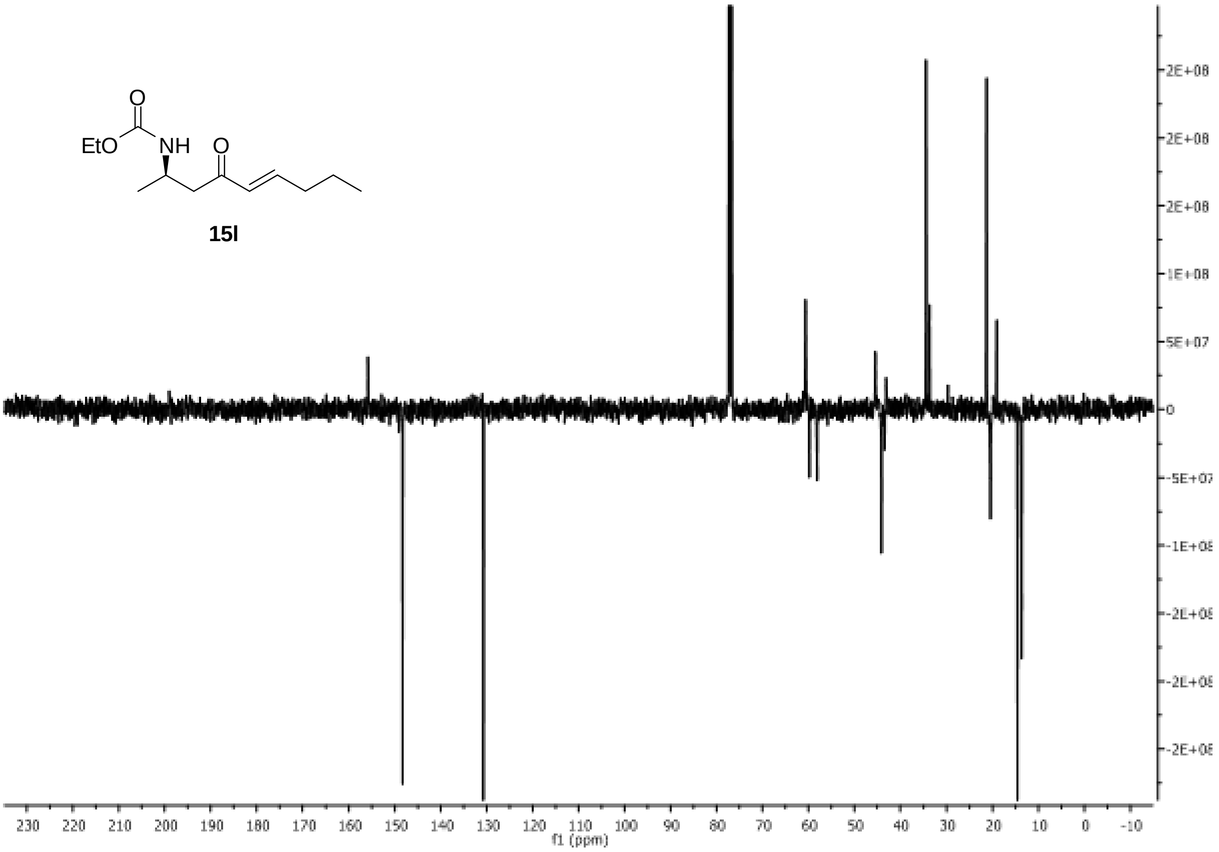
EtO
NH
O
15l
f1 (ppm)

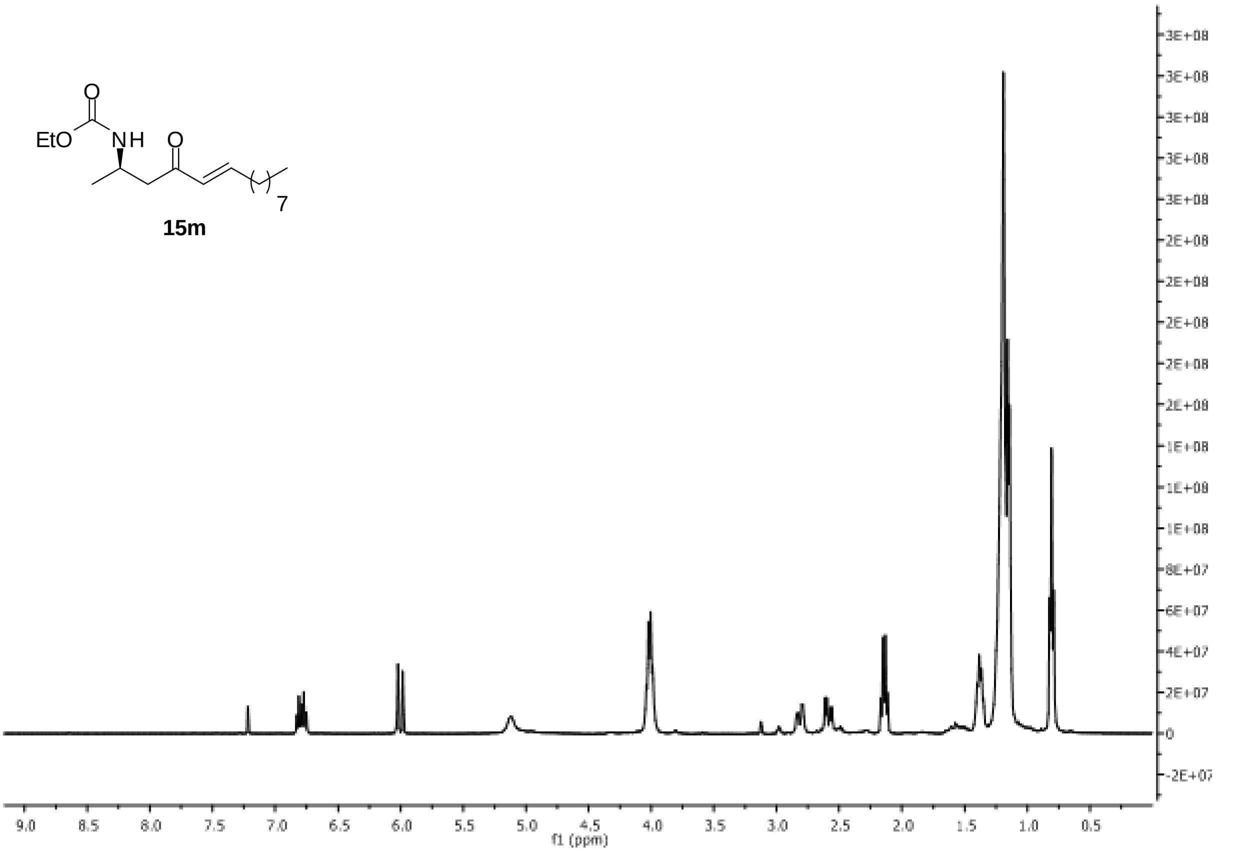
EtO
NH
O
7
15m
f1 (ppm)

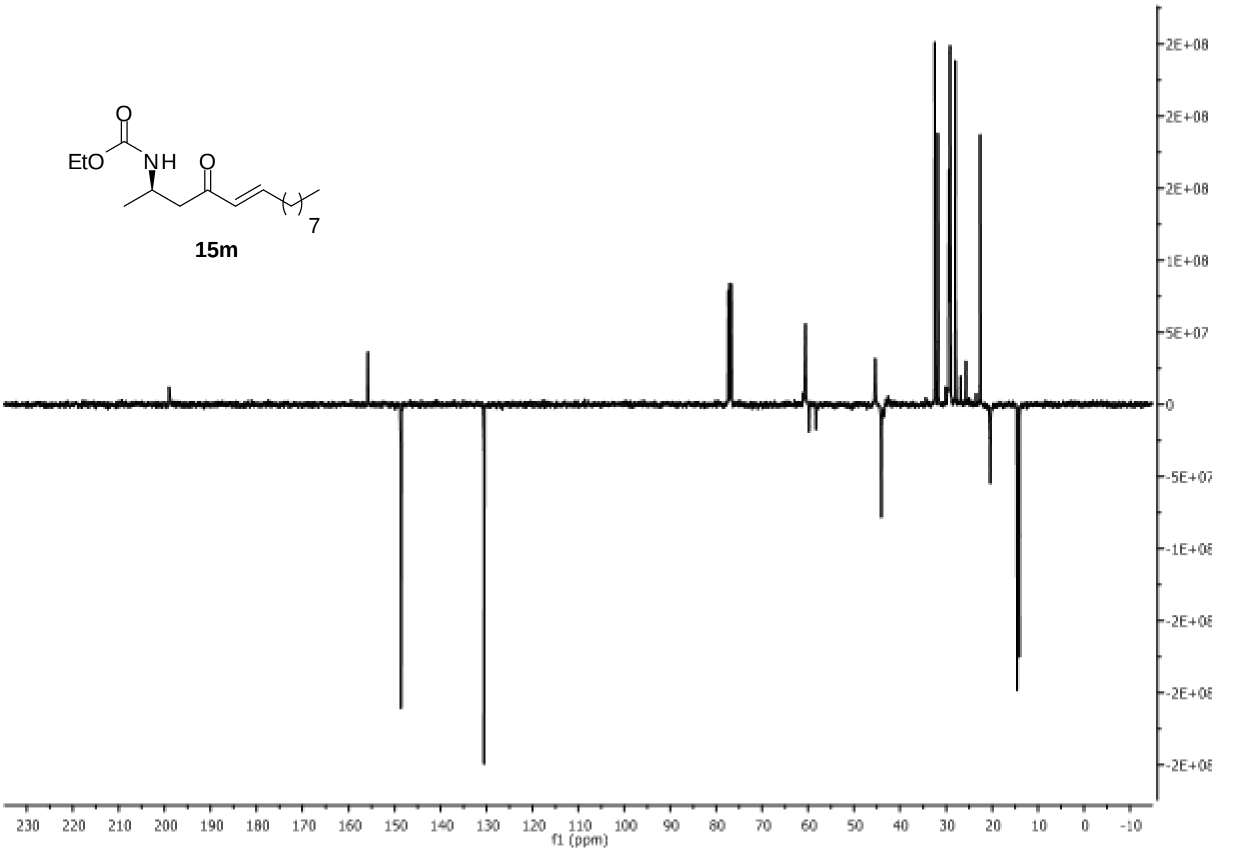
EtO
NH
O
7
15m
f1 (ppm)

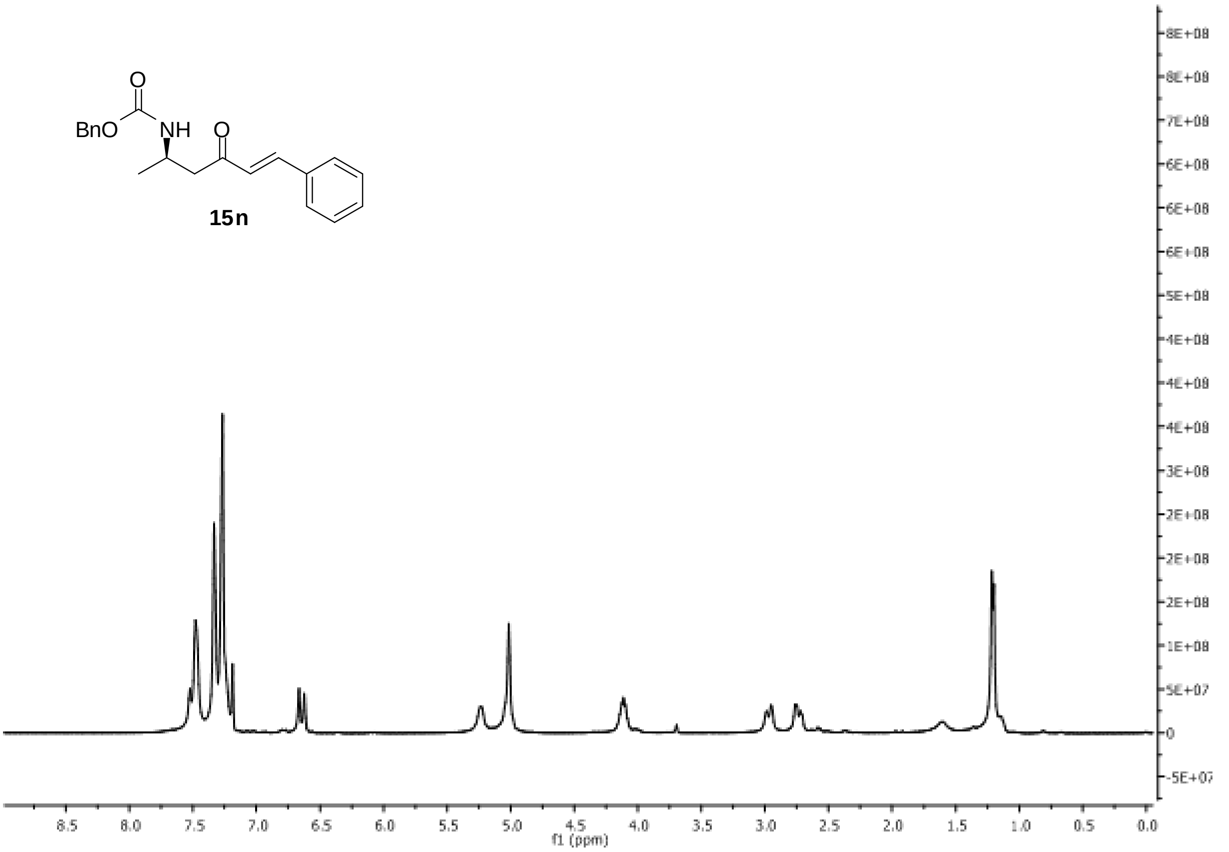
BnO
NH
O
O
15n
f1 (ppm)

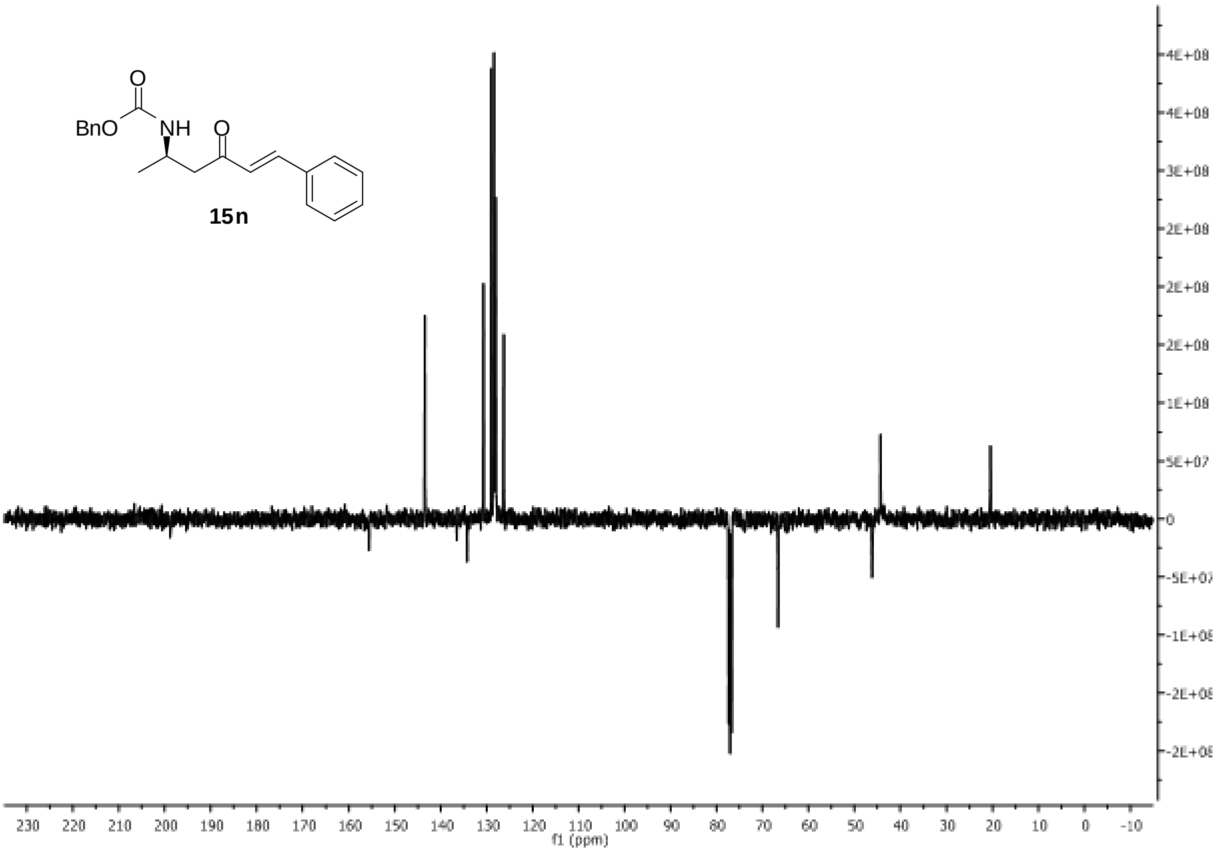
BnO
NH
O
O
15n
f1 (ppm)

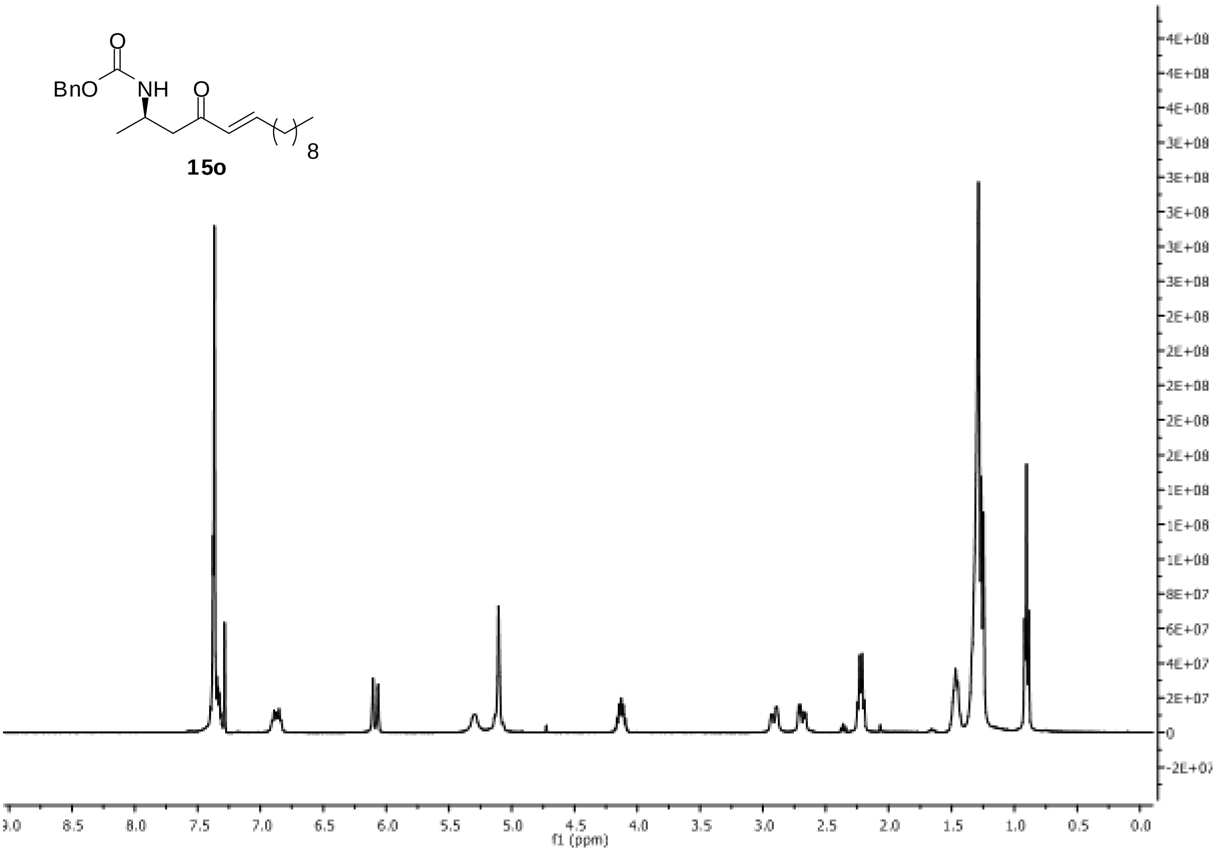
BnO
NH
O
O
8
15o
f1 (ppm)

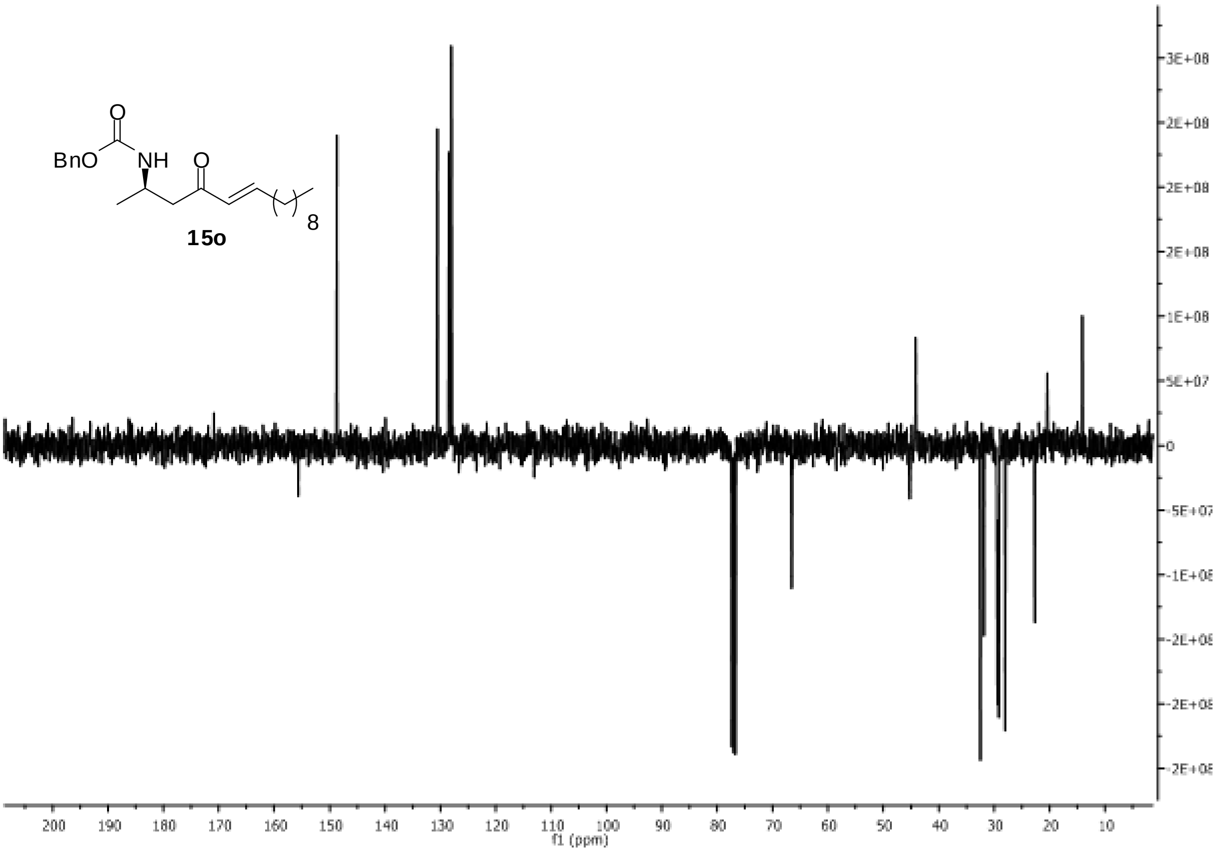
BnO
NH
O
O
8
15o
f1 (ppm)

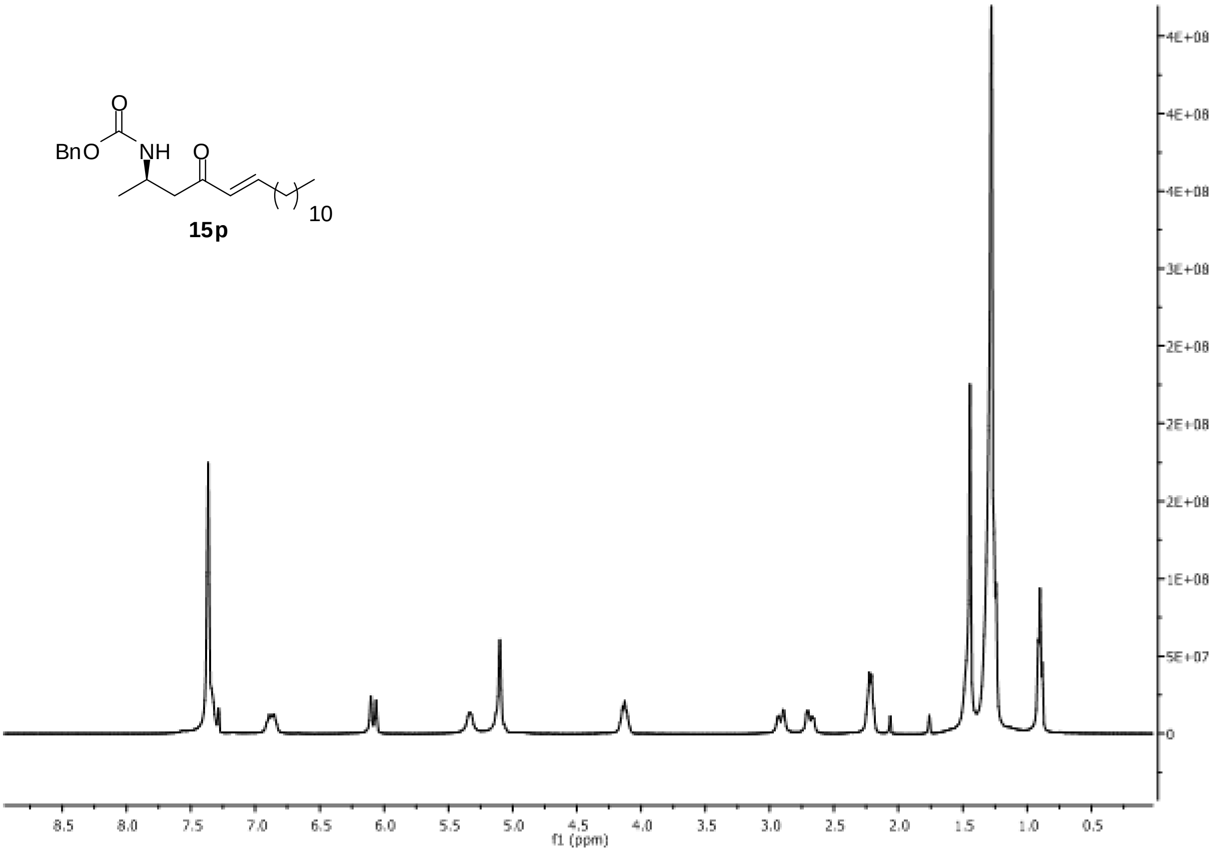
BnO
NH
O
O
10
15p
f1 (ppm)

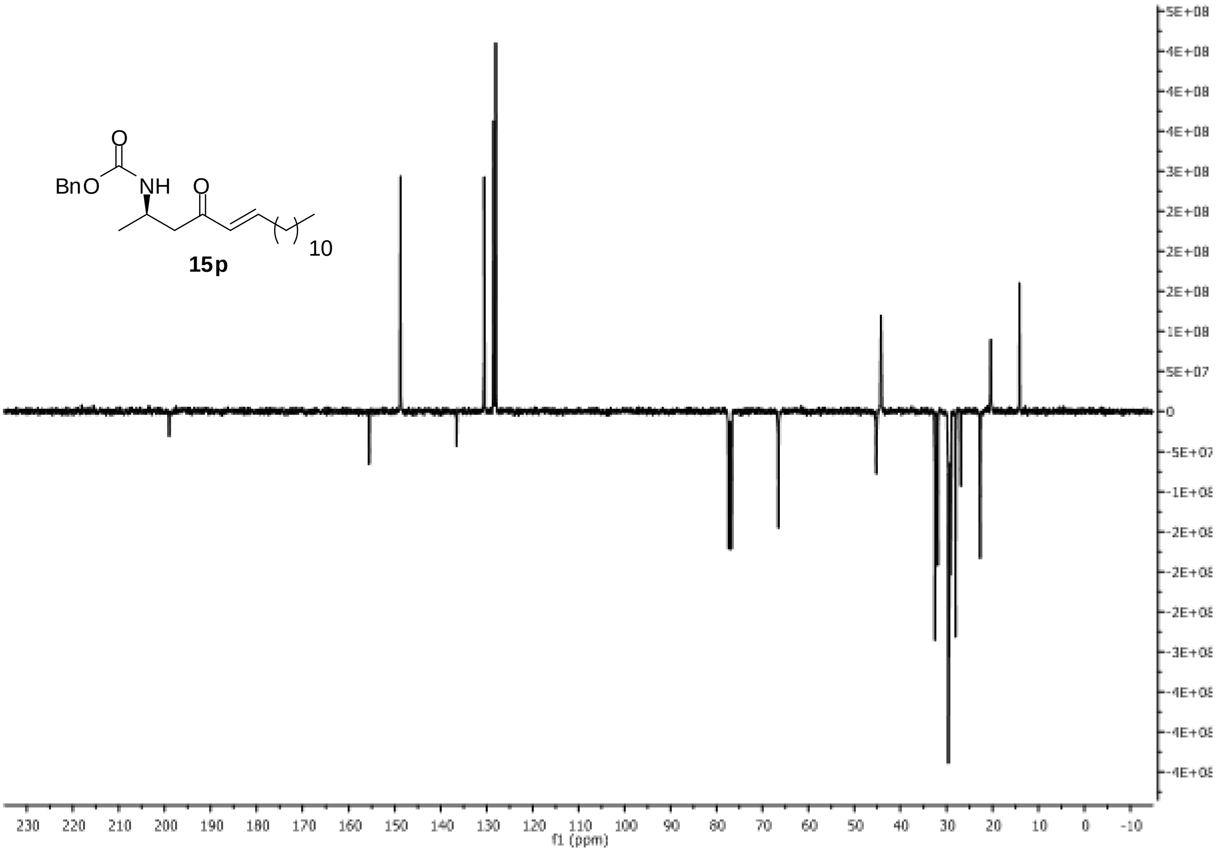
BnO
NH
O
O
10
15p
f1 (ppm)

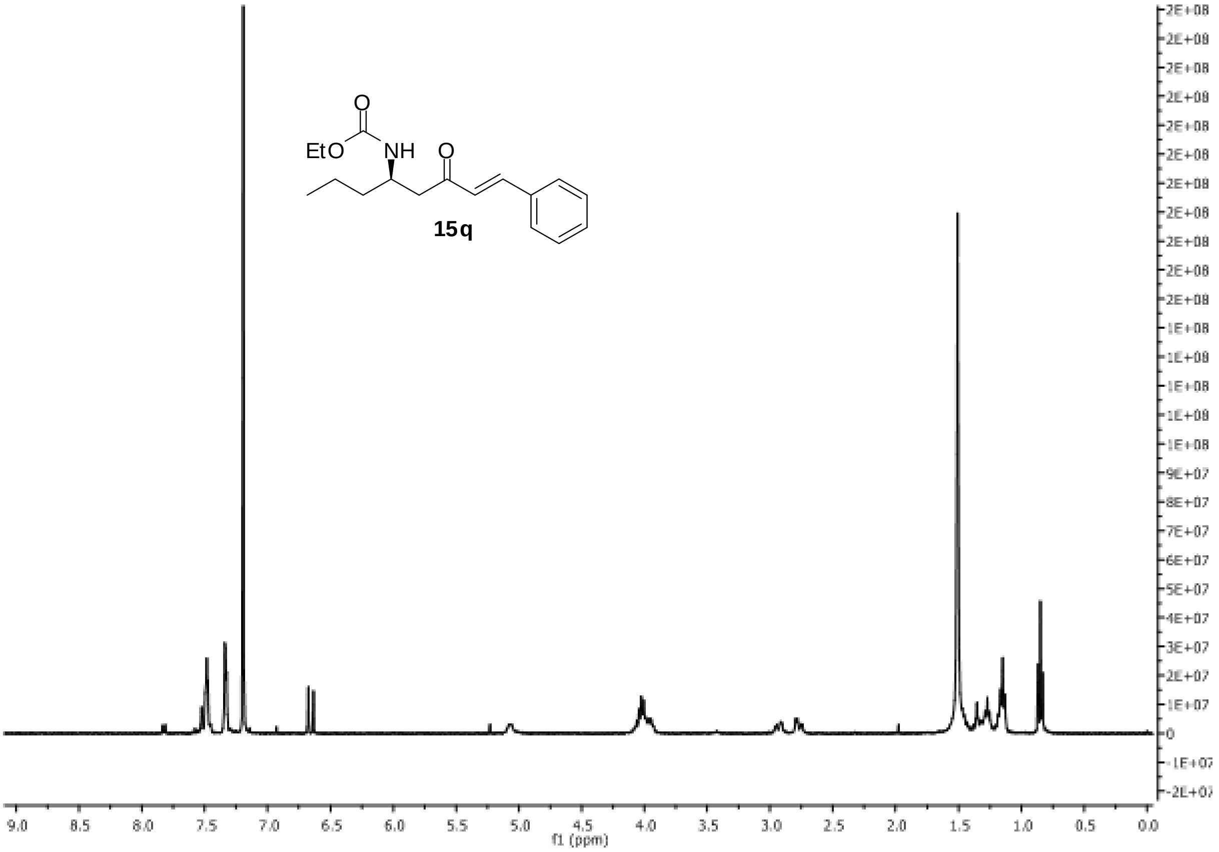
EtO
NH
O
O
15q
f1 (ppm)

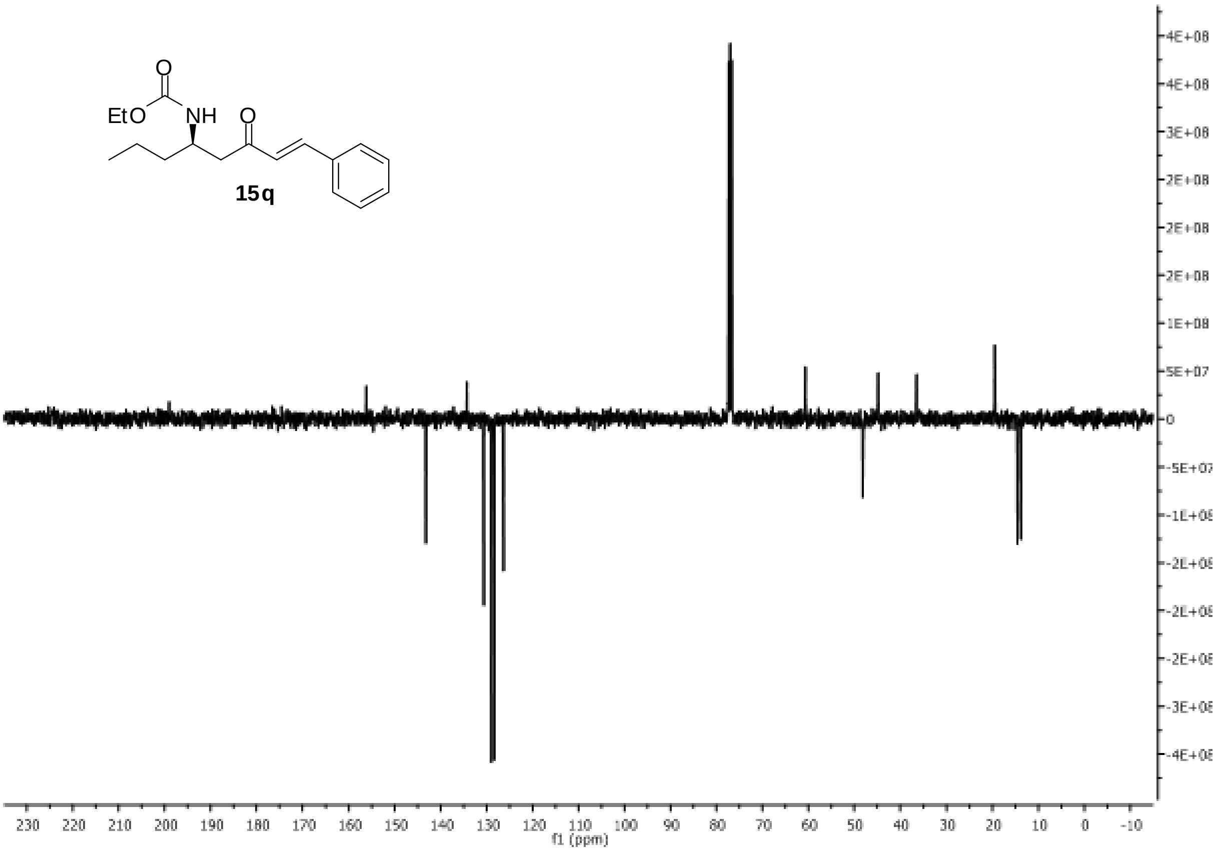
EtO
NH
O
O
15q
f1 (ppm)

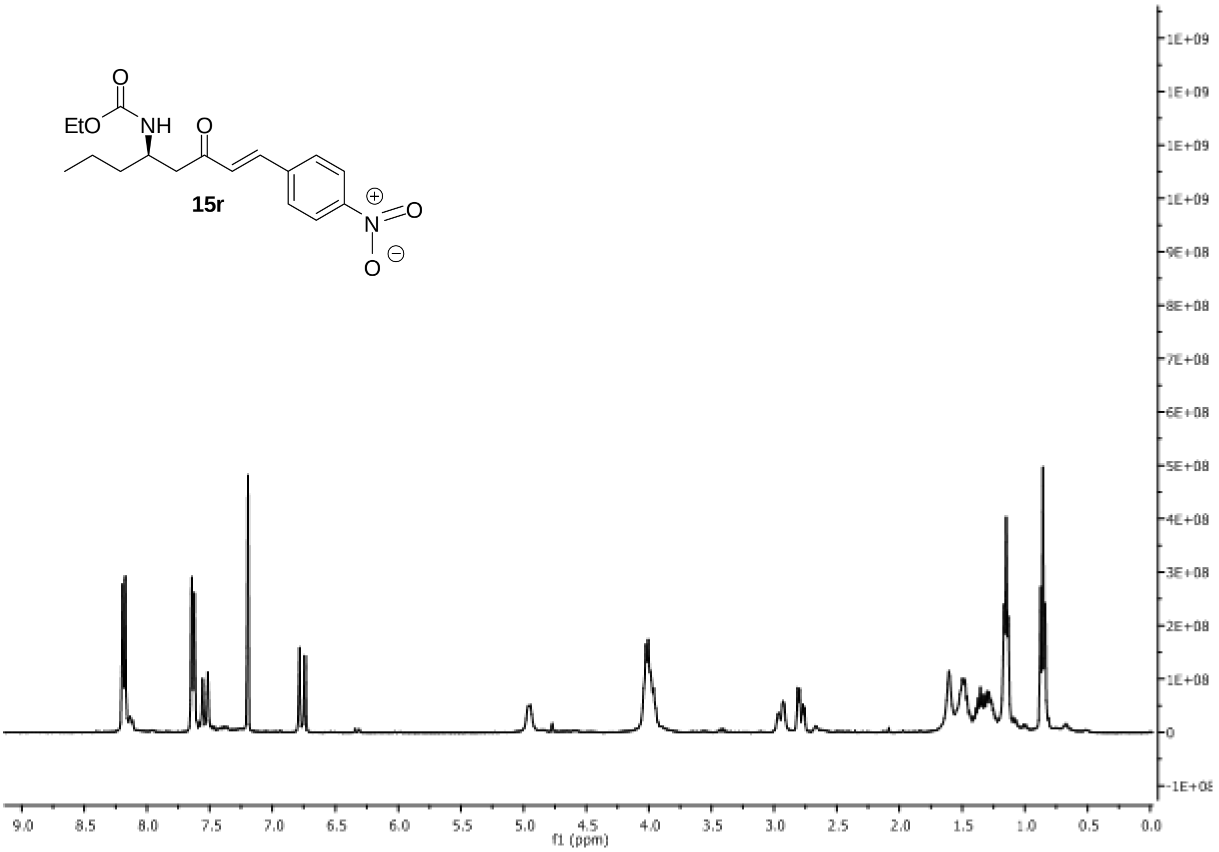
EtO
NH
O
O
15r
N
O
O
f1 (ppm)

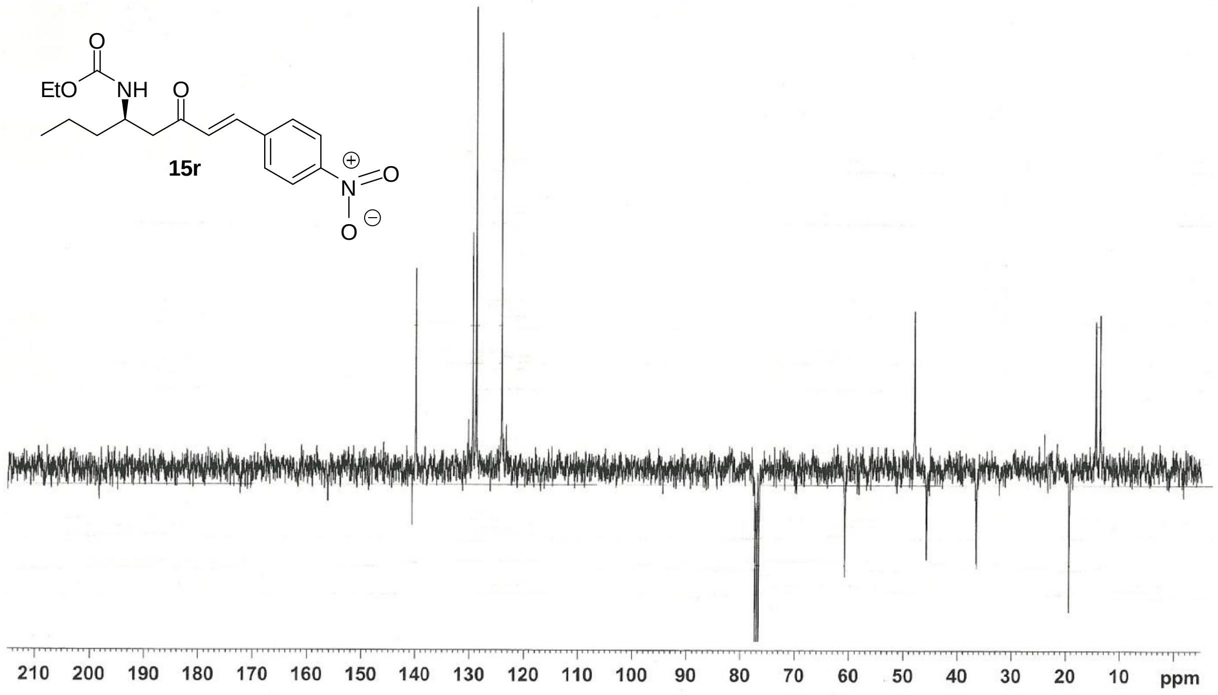
EtO
NH
O
O
15r
N
O
O
ppm

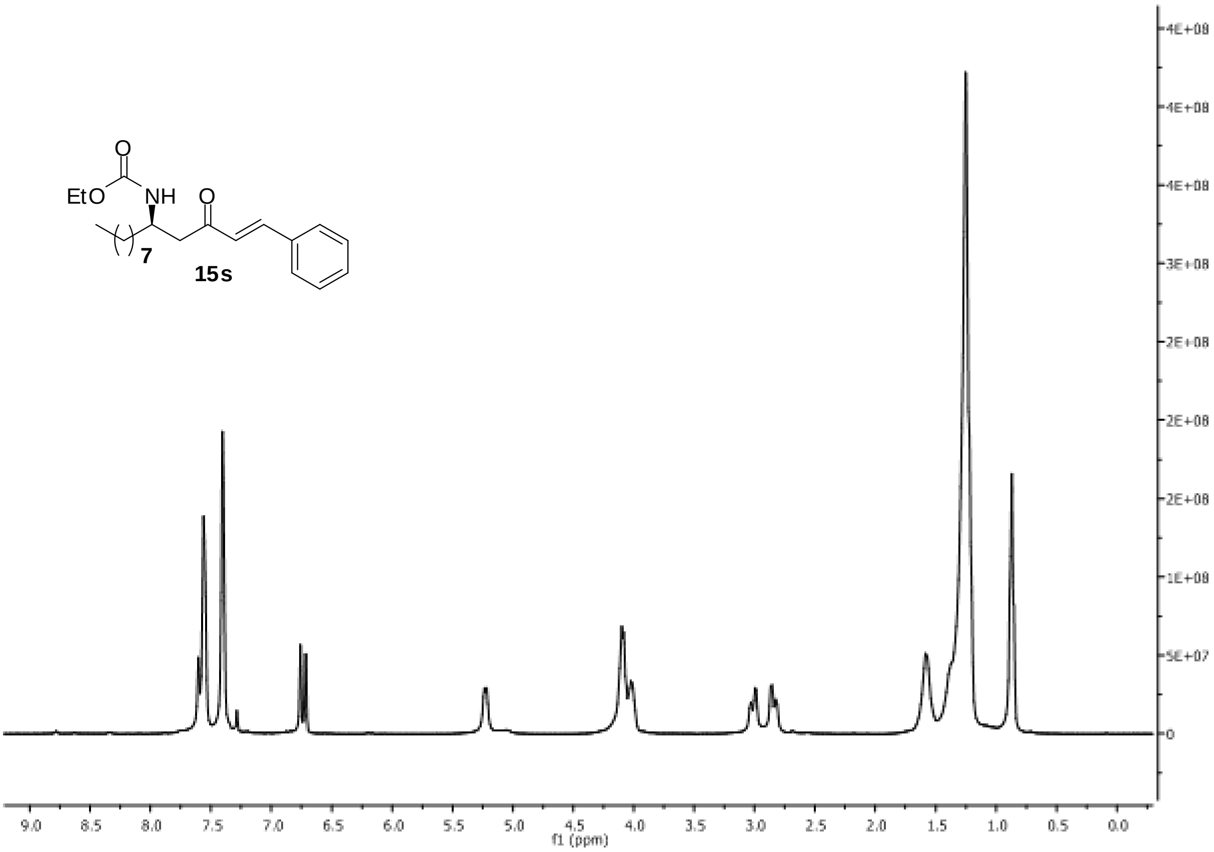
EtO
NH
O
O
7
15s
9.0
8.5
8.0
7.5
7.0
6.5
6.0
5.5
5.0
4.5
4.0
3.5
3.0
2.5
2.0
1.5
1.0
0.5
0.0
f1 (ppm)

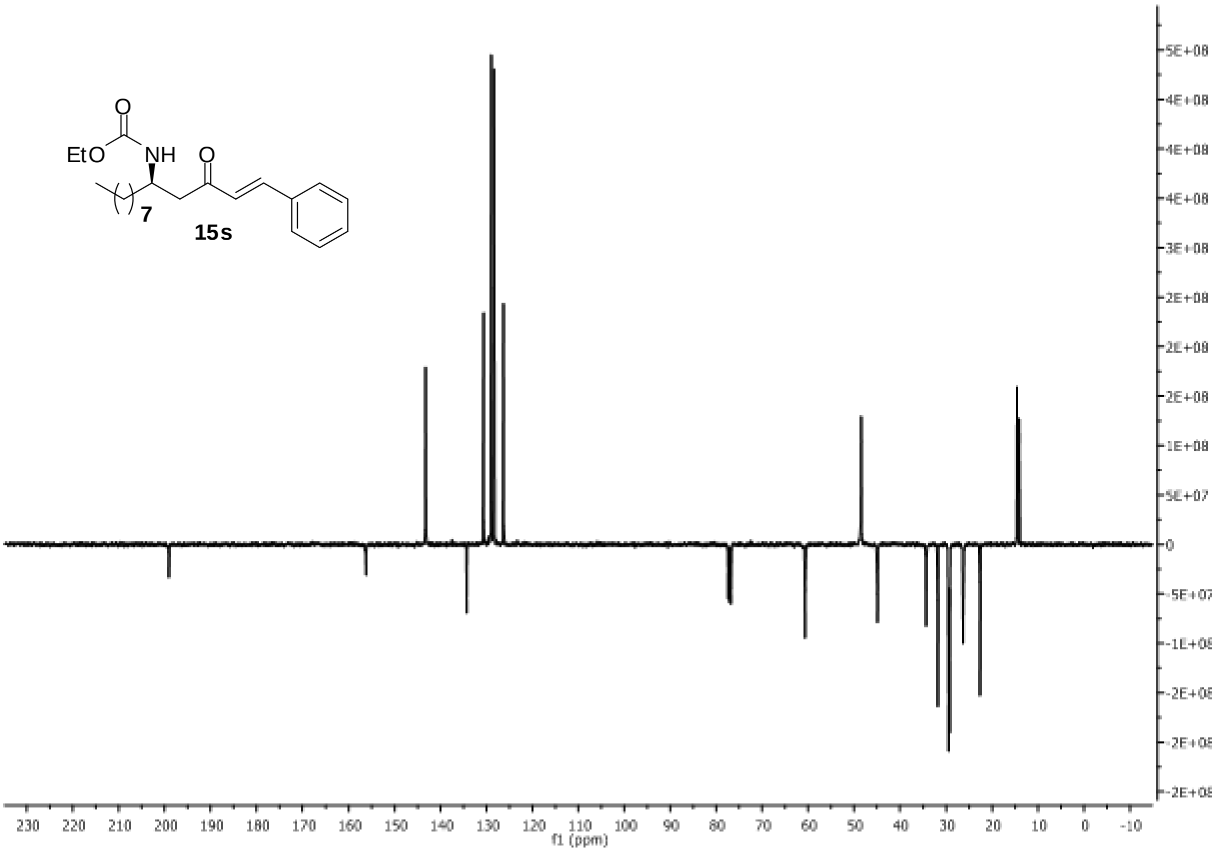
EtO
NH
O
O
7
15s
230
220
210
200
190
180
170
160
150
140
130
120
110
100
90
80
70
60
50
40
30
20
10
0
-10
f1 (ppm)

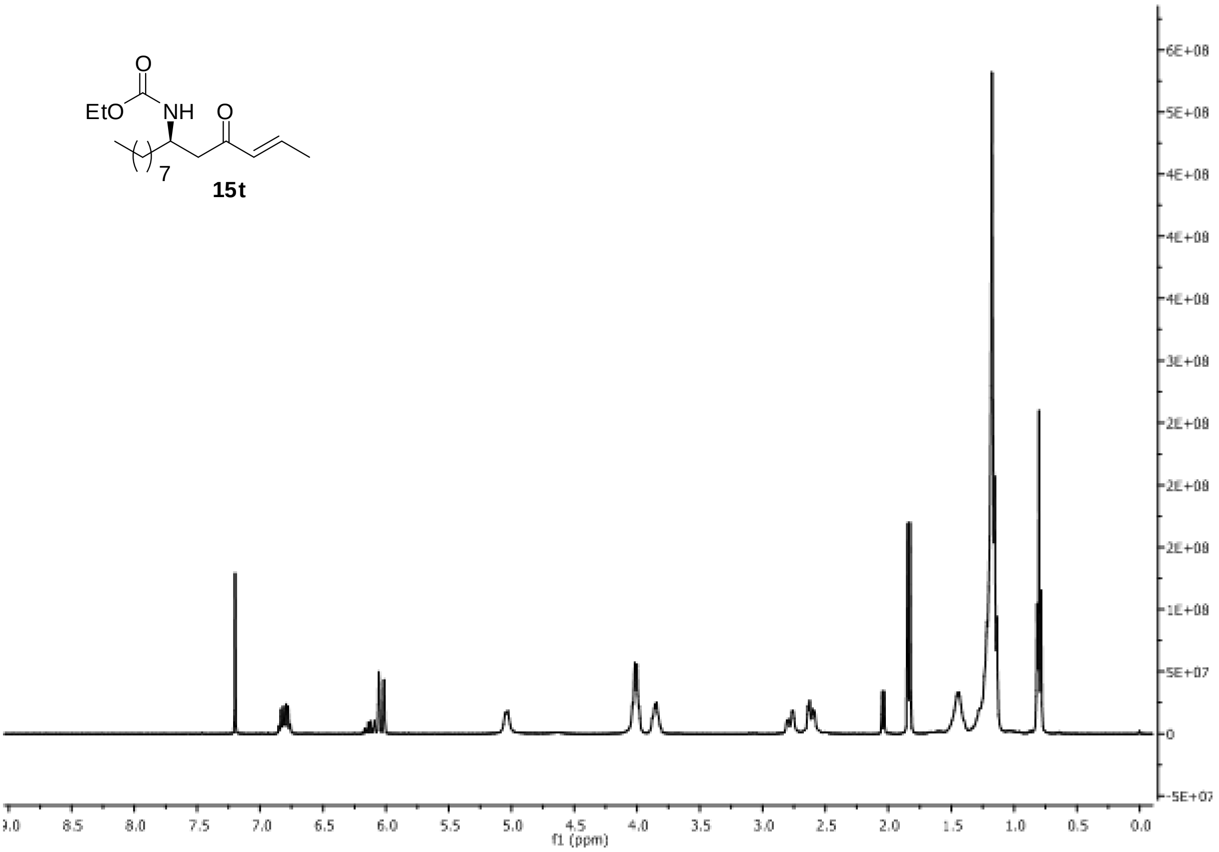
EtO
NH
O
7
15t
f1 (ppm)

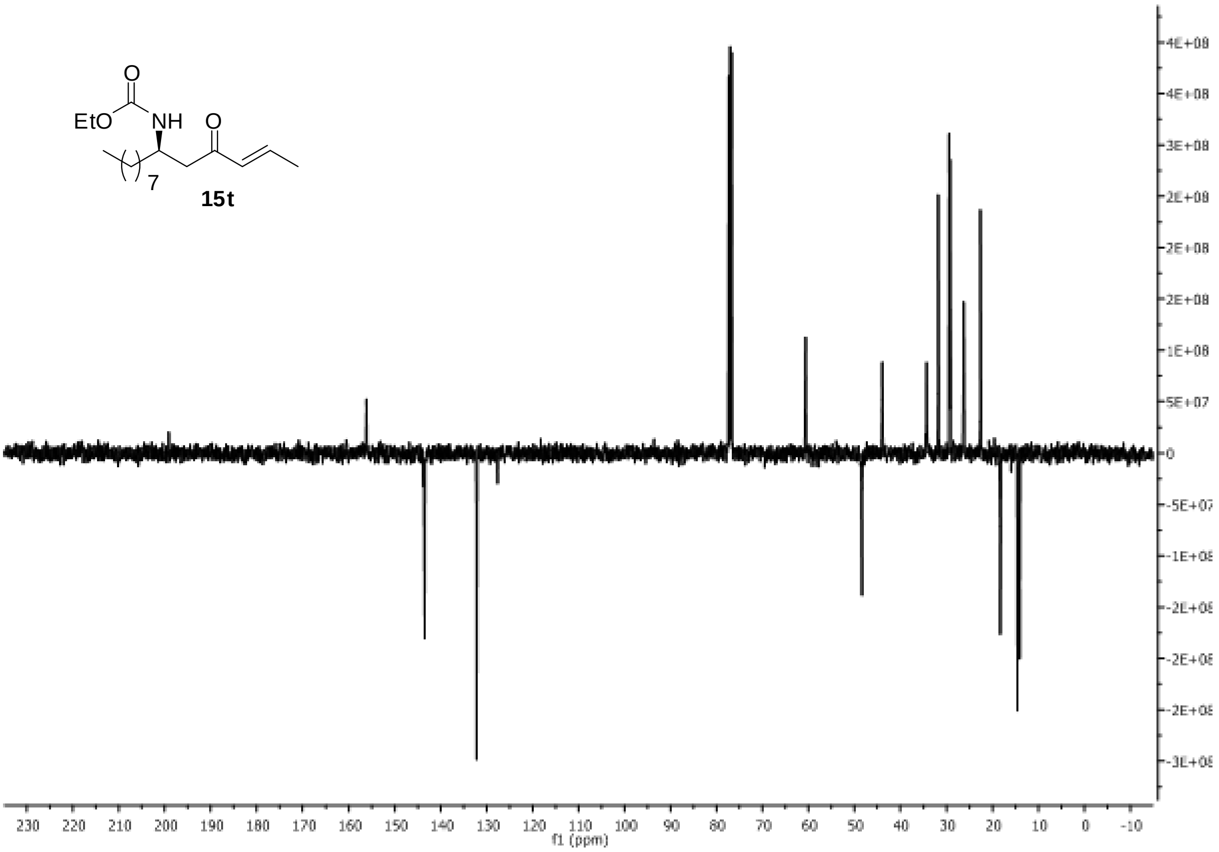
EtO
NH
O
7
15t
f1 (ppm)

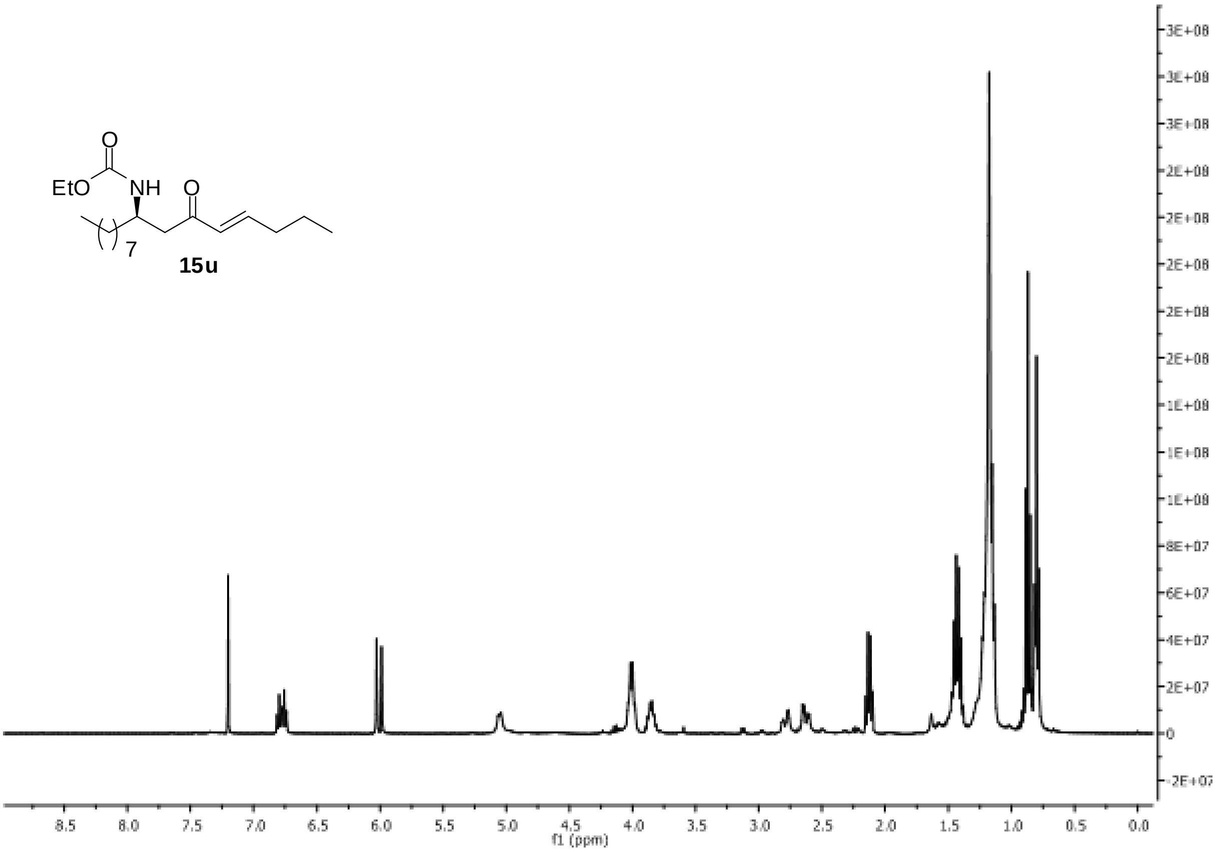

EtO
NH
O
O
7
15u
f1 (ppm)

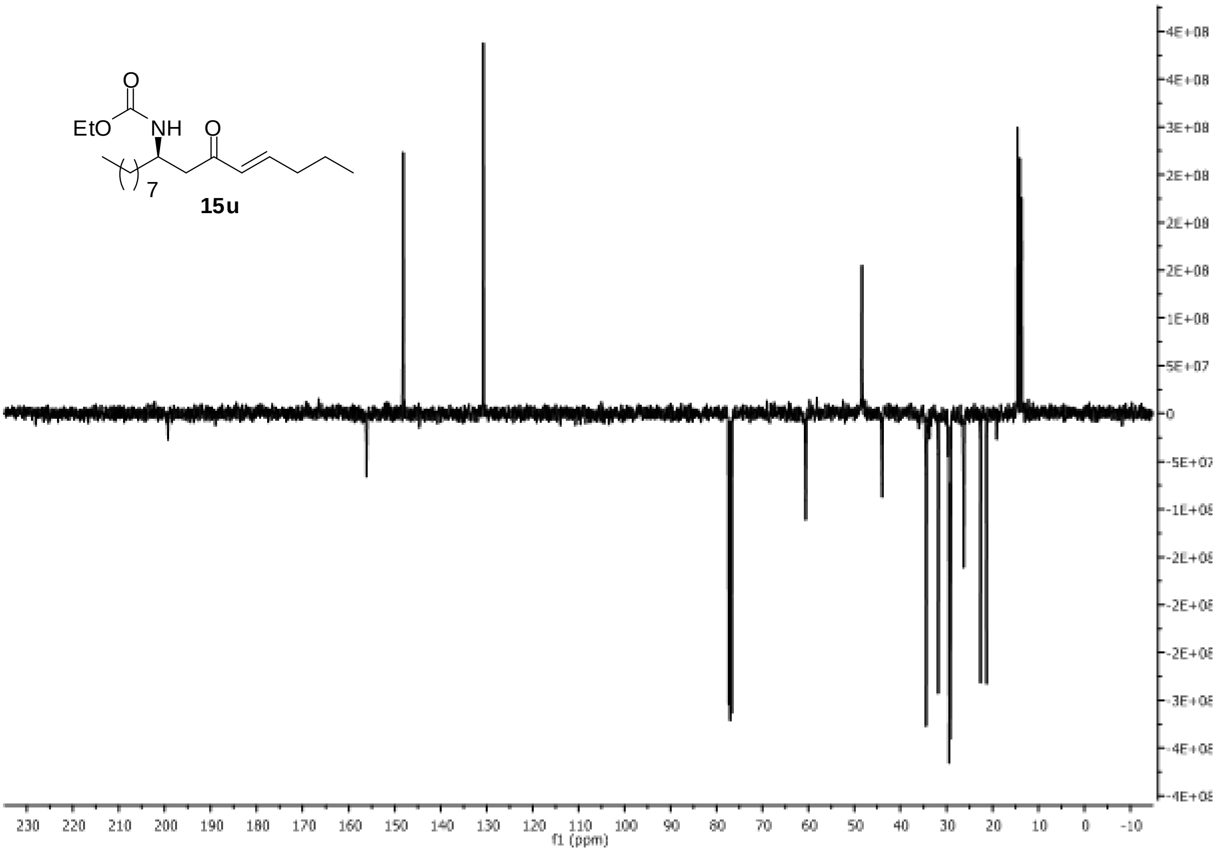

EtO
NH
O
O
7
15u
f1 (ppm)

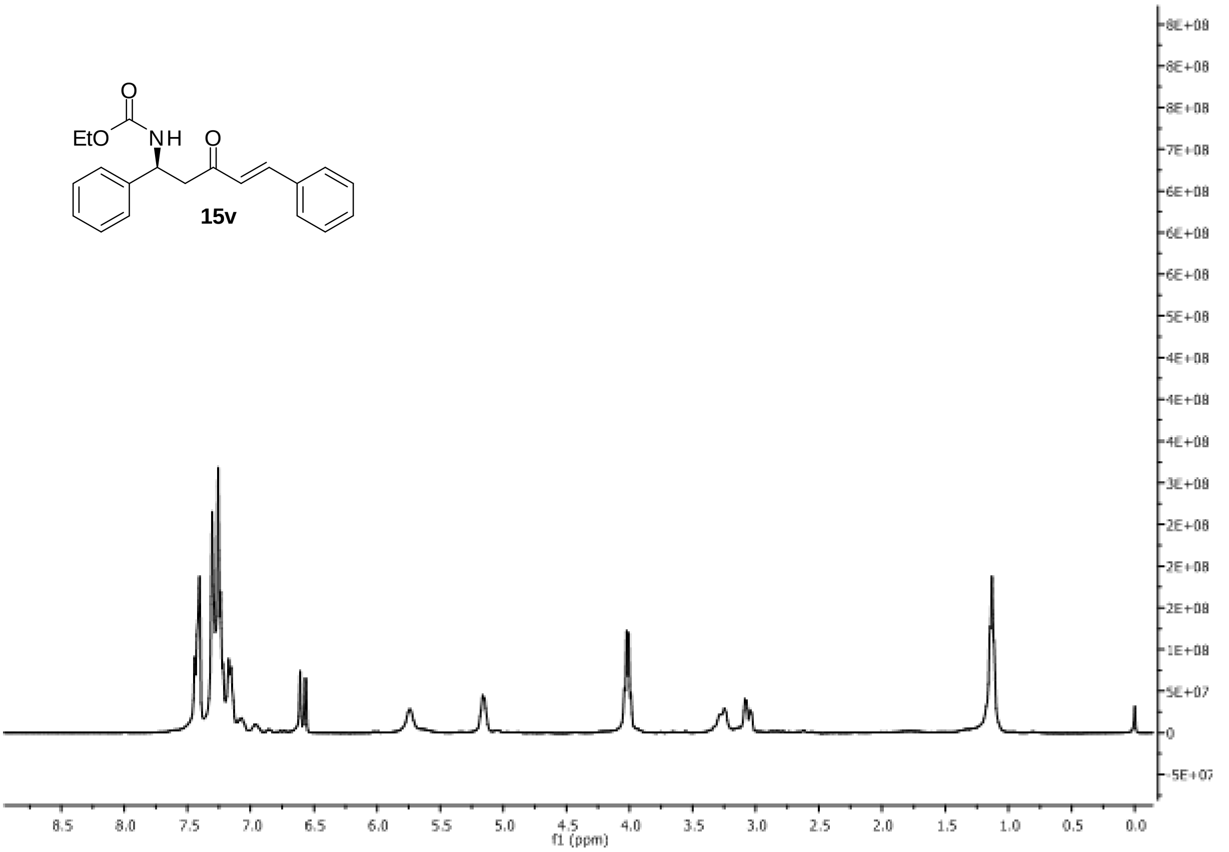
EtO
NH
O
O
15v
8.5 8.0 7.5 7.0 6.5 6.0 5.5 5.0 4.5 4.0 3.5 3.0 2.5 2.0 1.5 1.0 0.5 0.0
f1 (ppm)

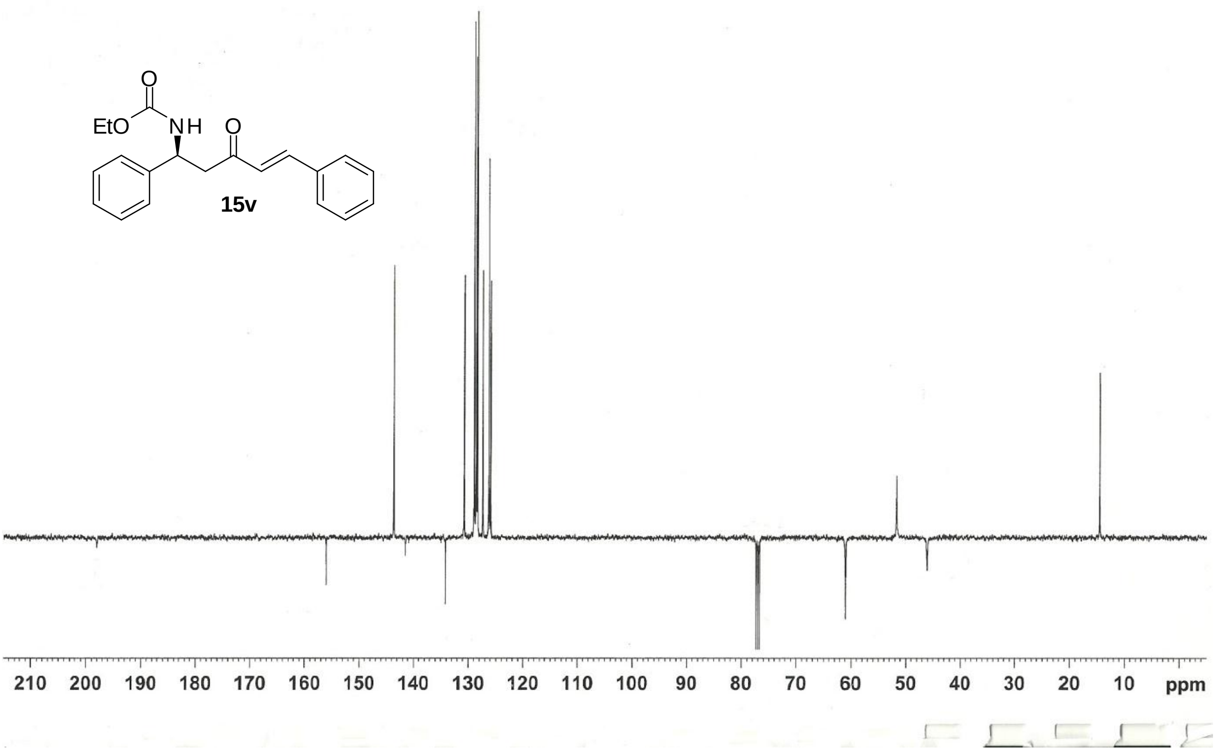
EtO
NH
O
O
15v
210 200 190 180 170 160 150 140 130 120 110 100 90 80 70 60 50 40 30 20 10 ppm

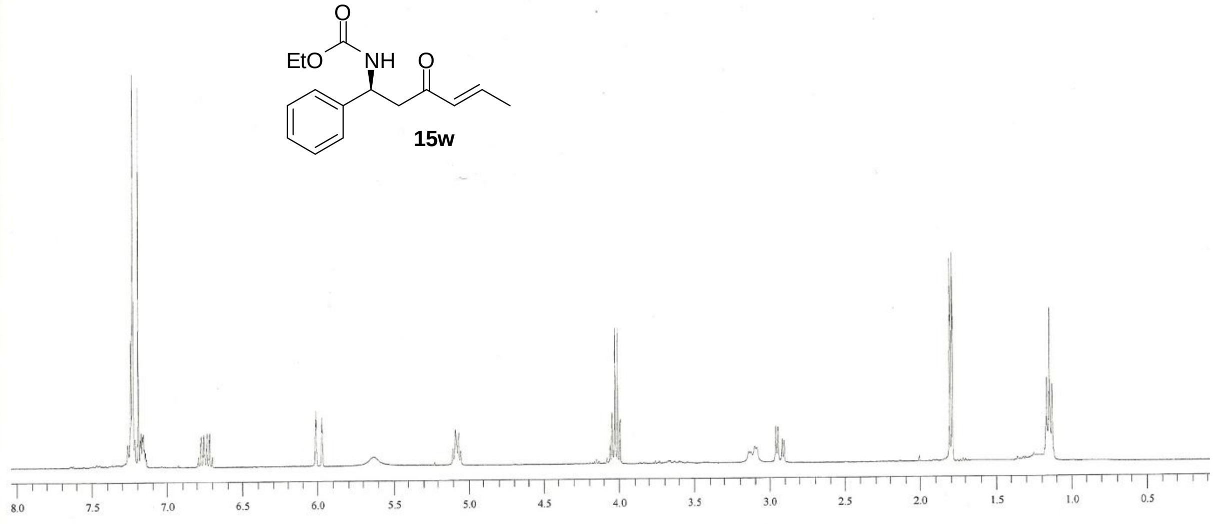
EtO
NH
O
O
15w
8.0
7.5
7.0
6.5
6.0
5.5
5.0
4.5
4.0
3.5
3.0
2.5
2.0
1.5
1.0
0.5

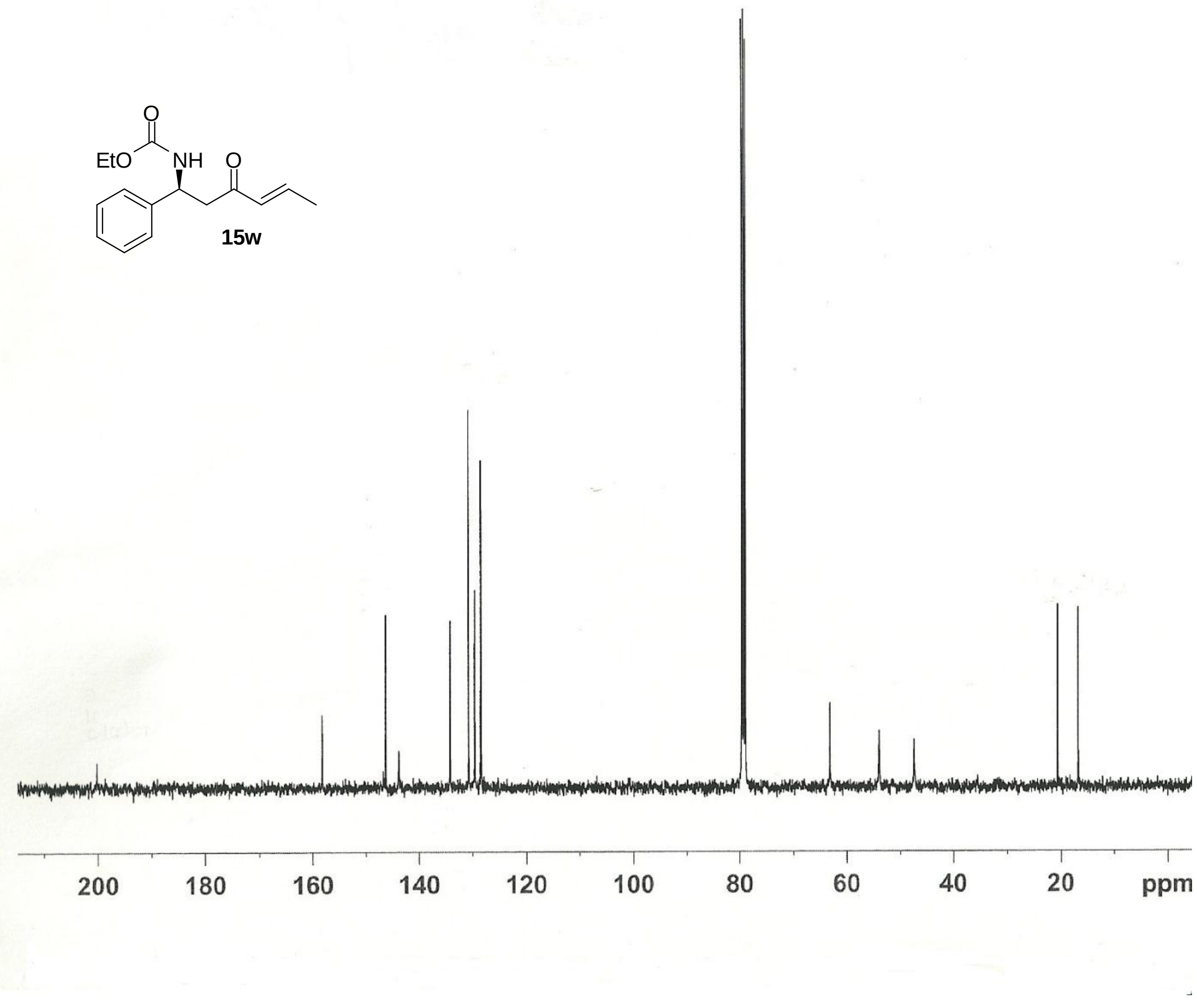
EtO
NH
O
O
15w
200
180
160
140
120
100
80
60
40
20
ppm

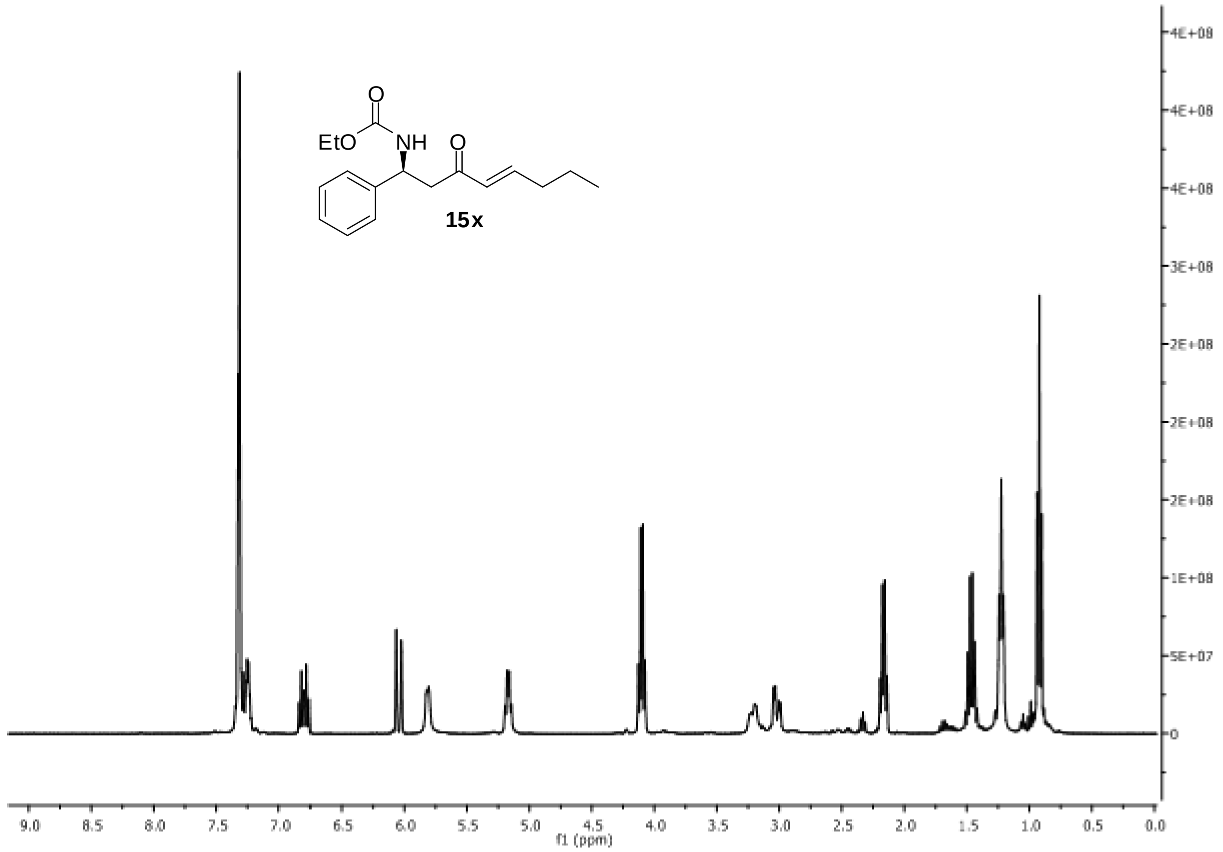
EtO
NH
O
O
15x
f1 (ppm)

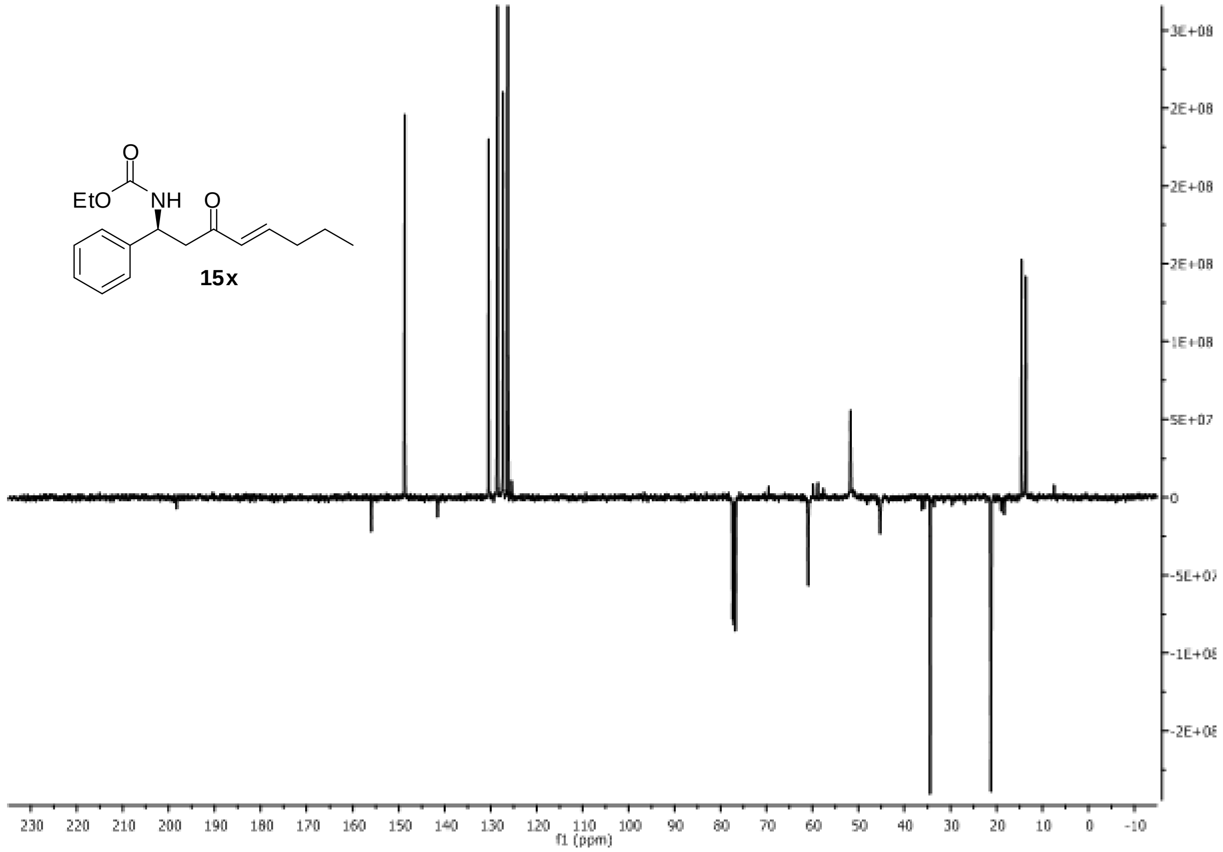
EtO
NH
O
O
15x
f1 (ppm)

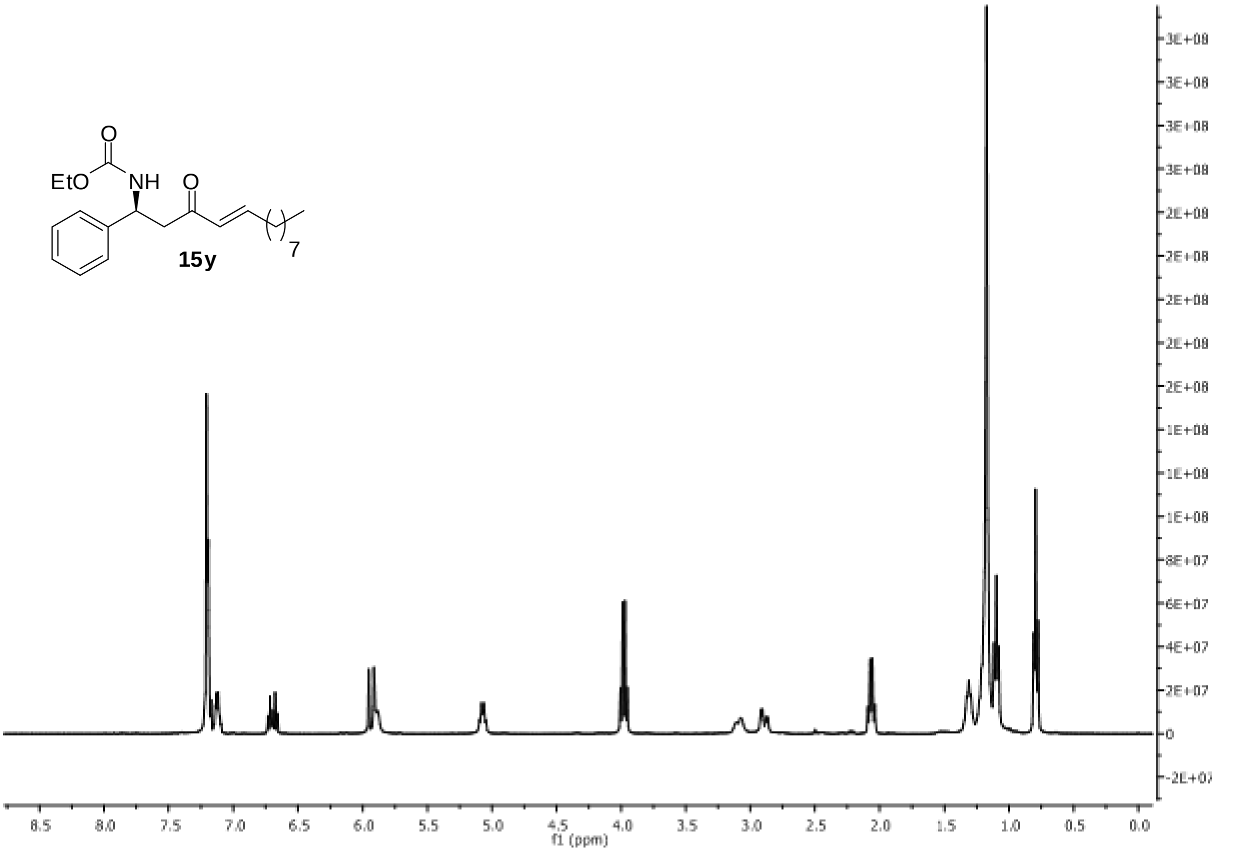
O
EtO
NH
O
7
15y
f1 (ppm)

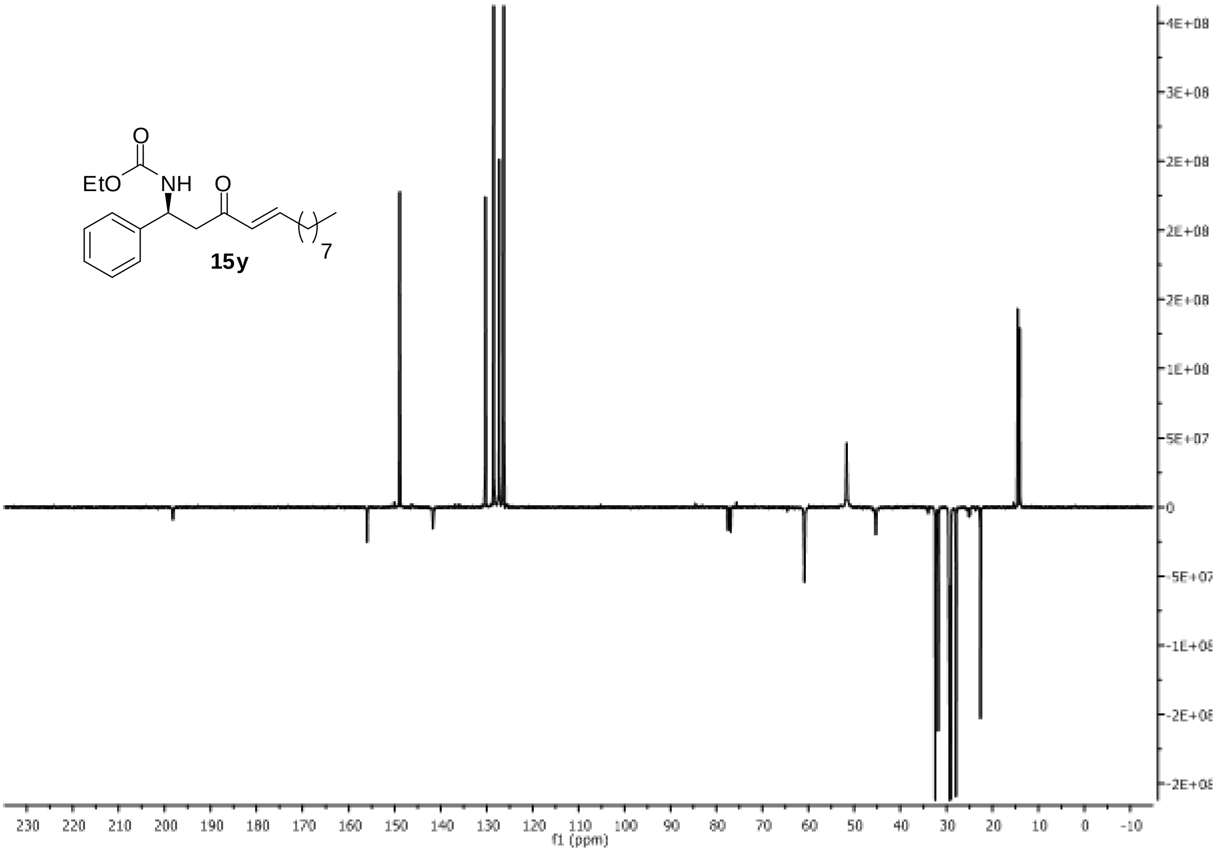
O
EtO
NH
O
7
15y
f1 (ppm)

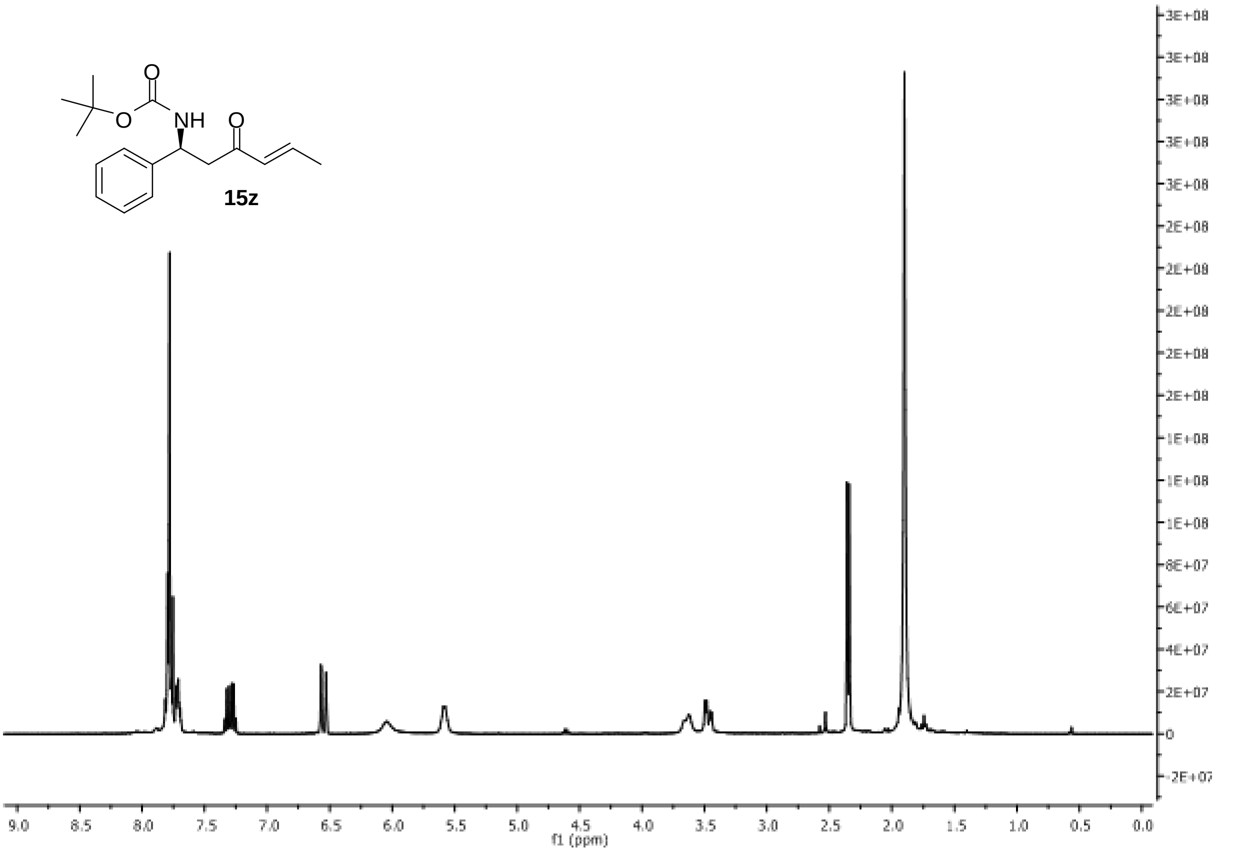

15z
f1 (ppm)

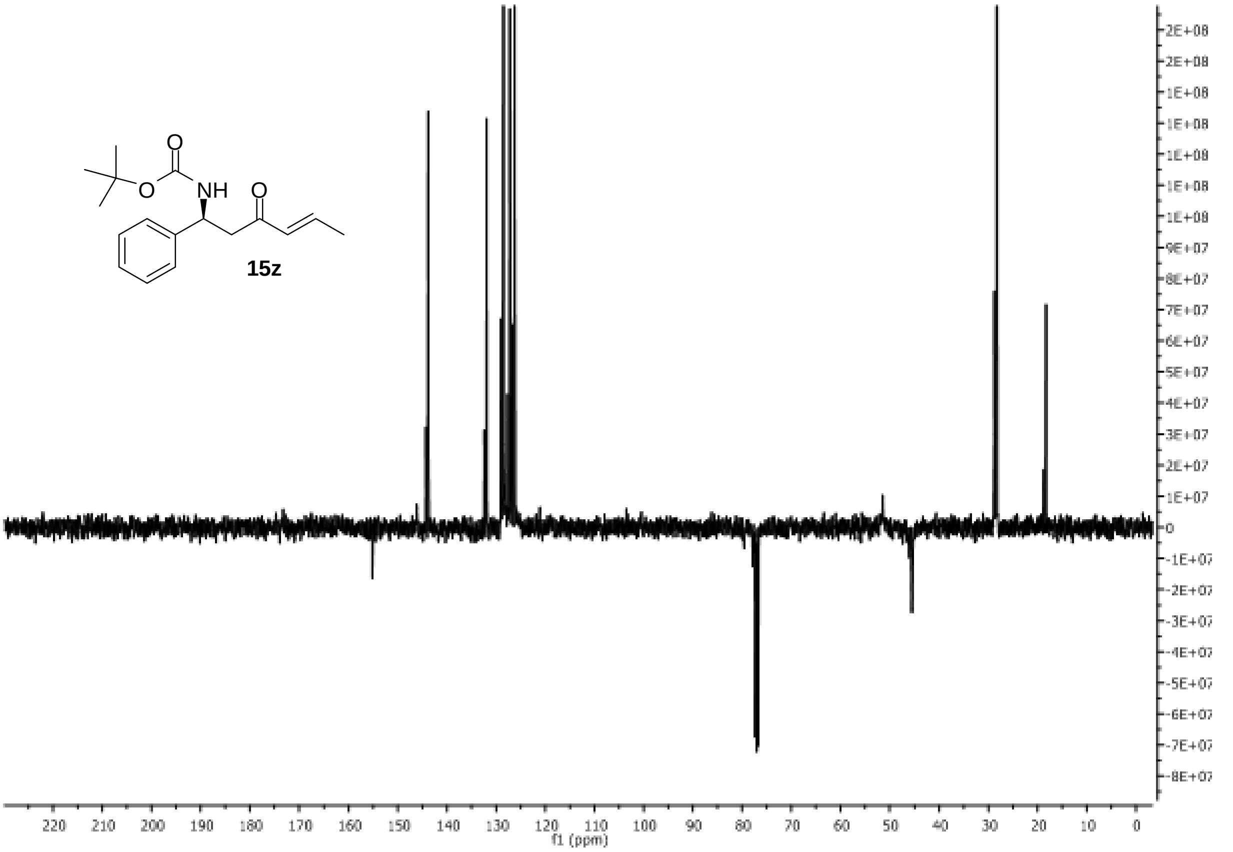

15z
f1 (ppm)

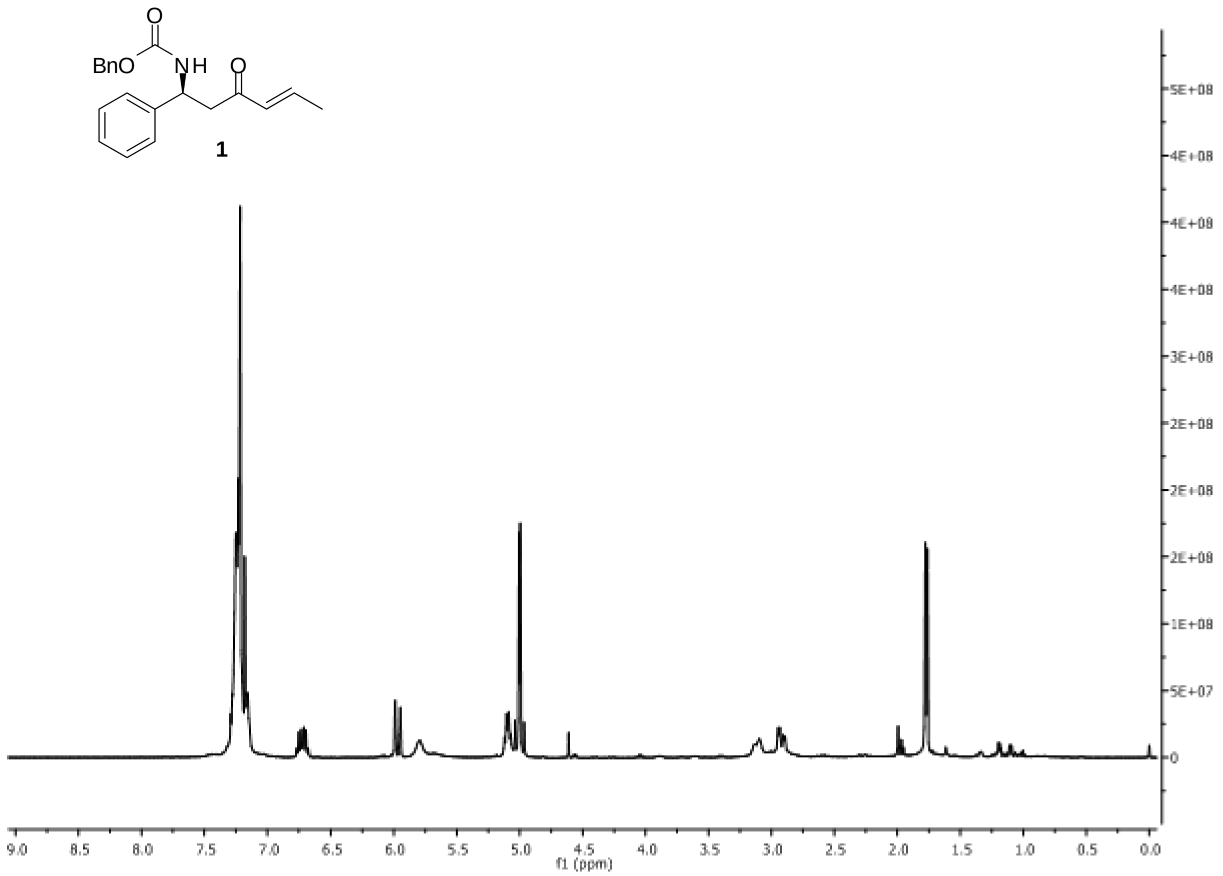
BnO
NH
O
1
f1 (ppm)

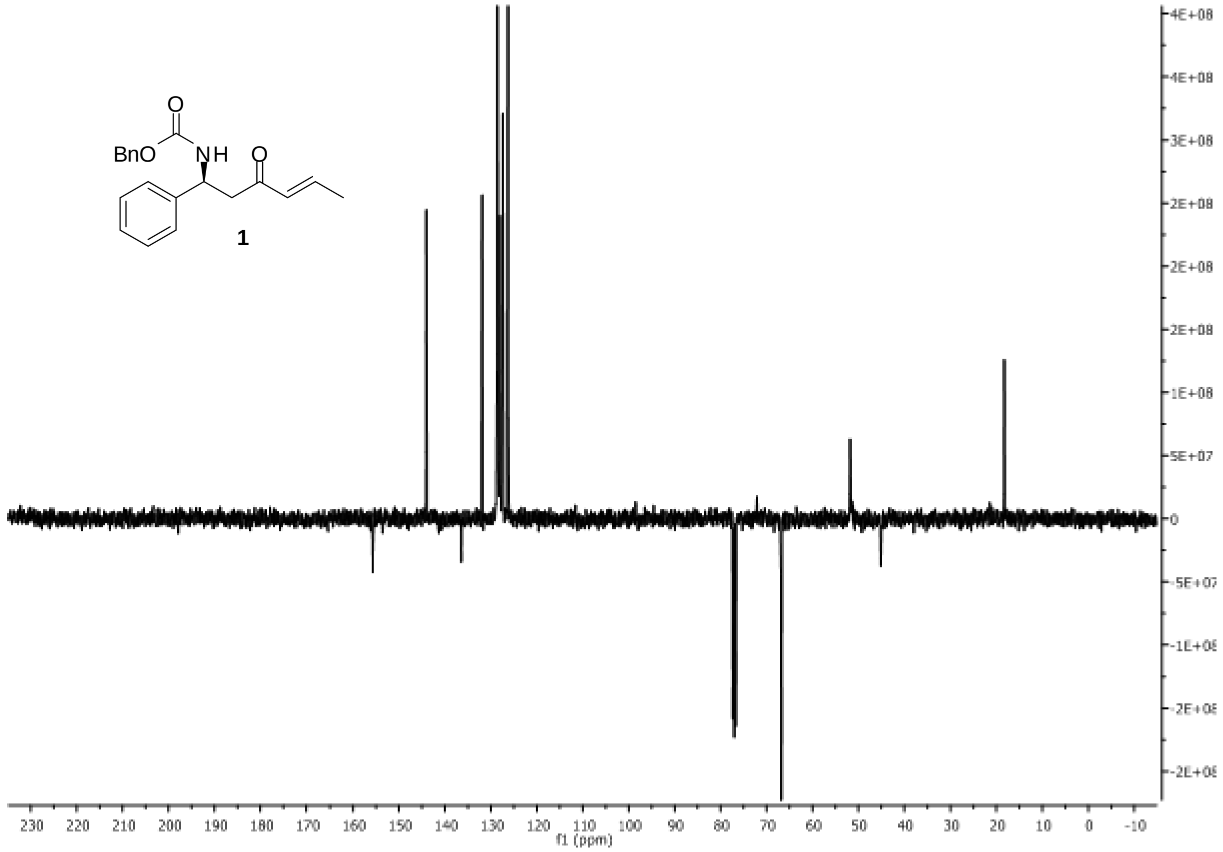
BnO
NH
O
1
f1 (ppm)
